# Covalent Organic Framework and Carbon Nitride Composite for Scalable Solar Reforming

**DOI:** 10.1002/adma.202513457

**Published:** 2025-09-26

**Authors:** Suvendu Karak, Yongpeng Liu, Ariffin Bin Mohamad Annuar, Erwin Reisner

**Affiliations:** ^1^ Yusuf Hamied Department of Chemistry University of Cambridge Cambridge CB2 1EW UK

**Keywords:** carbon nitride, covalent organic framework, plastic valorization, solar reforming, Z‐scheme photocatalyst

## Abstract

Solar reforming uses sunlight to valorize waste and offers a promising route to circular chemistry, but most current systems operate under corrosive conditions, lack scalability and processability, or suffer from poor selectivity in oxidative waste valorization. Here, a modular composite photocatalyst, comprising a crystalline covalent organic framework (COF), cyanamide‐functionalized carbon nitride (CN_x_), indium tin oxide (ITO), and a nickel‐based molecular catalyst (NiME), capable of driving selective waste valorization and H_2_ generation using visible light under pH neutral aqueous conditions is reported. The porous structure of the COF provides nanoconfinement, thereby supporting the targeted oxidation of polyols and sugars into formate (HCOO^−^), while carbon nitride in the presence of the molecular catalyst facilitates H_2_ evolution. ITO functions as a solid‐state electron mediator, enabling coupled oxidative and reductive processes for the concurrent solar‐driven production of H_2_ and HCOO^−^. The high processability of the COF|ITO|CN_x_|NiME composite allows for the construction of a standalone photoleaf and photopanel, which have been integrated into a single‐window and a scalable multi‐window reactor, respectively. Continuous operability over seven days, along with testing under outdoor conditions with pretreated plastic under direct sunlight, offers a pathway toward practical and scalable solar reforming using structurally tunable organic semiconductor composites.

## Graphical abstract

A modular composite, containing a covalent organic framework, indium tin oxide, carbon nitride and nickel based molecular catalyst, enables solar reforming under neutral aqueous conditions, converting polyols and sugars into formate while generating H_2_. The composite forms scalable photoleaf and photopanel devices, achieving continuous solar‐driven waste valorization and hydrogen production outdoors.

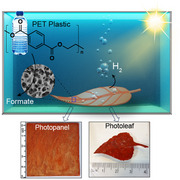

## 1 Introduction

Artificial photosynthesis has long been considered as a potential solution to provide chemical fuel from the direct conversion of sunlight, water, and carbon dioxide. Yet, the practical realization remains a formidable challenge, primarily due to the thermodynamically demanding and kinetically sluggish oxygen evolution reaction (H_2_O → H_2_ + 1/2O_2_;   ΔG^0^ ≈ 237 kJ mol^−1^ at 25 °C).^[^ ^1^ ^]^ As a promising alternative, solar reforming couples the reductive conversion of protons or CO_2_ (2H^+^ → H_2_ or CO_2_ → products) with the oxidative valorization of organic waste, offering thermodynamically favorable pathways (C_x_H_y_O_z_ + (2x − z)H_2_O → (2x − z + y/2)H_2_ + xCO_2_;   ΔG^0^ ≈ 0 kJ mol^−1^at 25 °C).^[^ ^2^ ^]^ This approach also mitigates waste while generating value‐added products, positioning solar reforming as a dual‐benefit strategy for solar fuel generation and circular carbon utilization.

Recently, various metal oxides and polymeric semiconductors have been explored for photoreforming under UV‐visible light irradiation. In particular, TiO_2_, ZnS, CdS, ZnCdS, carbon dots, graphene oxide dots, covalent triazine frameworks, and carbon nitride‐based systems, coupled with various cocatalysts, have been investigated to photoreform alcohols, sugars, pretreated food waste, and plastics.^[^ ^3^ ^]^ However, these systems need specific solvents such as highly alkaline solutions or buffered media for a pH‐controlled environment to maintain the optimal performance. They often require noble metal cocatalysts, exhibit limited product selectivity and structural stability and typically operate in powdered form. To address the latter, metal oxides and their various composite powders have been used for the construction of supported panels for sunlight driven H_2_ evolution and CO_2_ reduction coupled with water oxidation.^[^ ^4^ ^]^ Although, carbon nitride‐based glass‐supported photopanels have been recently realized for photoreforming,^[^ ^5^ ^]^ developing a generalized strategy for standalone panel fabrication (without any support) would be desirable.

In this context, a polymer‐based, processable synthetic methodology that can integrate structurally and functionally diverse catalytic components offers a promising route to address the existing limitations in photoreforming systems. In particular, the construction of Z‐scheme‐inspired architectures—where two redox‐complementary semiconductors are coupled through a mediator—can be fundamentally advantageous in harvesting solar energy.^[^ ^6^ ^]^ Z‐scheme designs also allow independent optimization of the oxidation and reduction sites, thereby allowing improvements to reaction selectivity and flexibility in material selection.^[^ ^7^ ^]^

**Figure 1:**
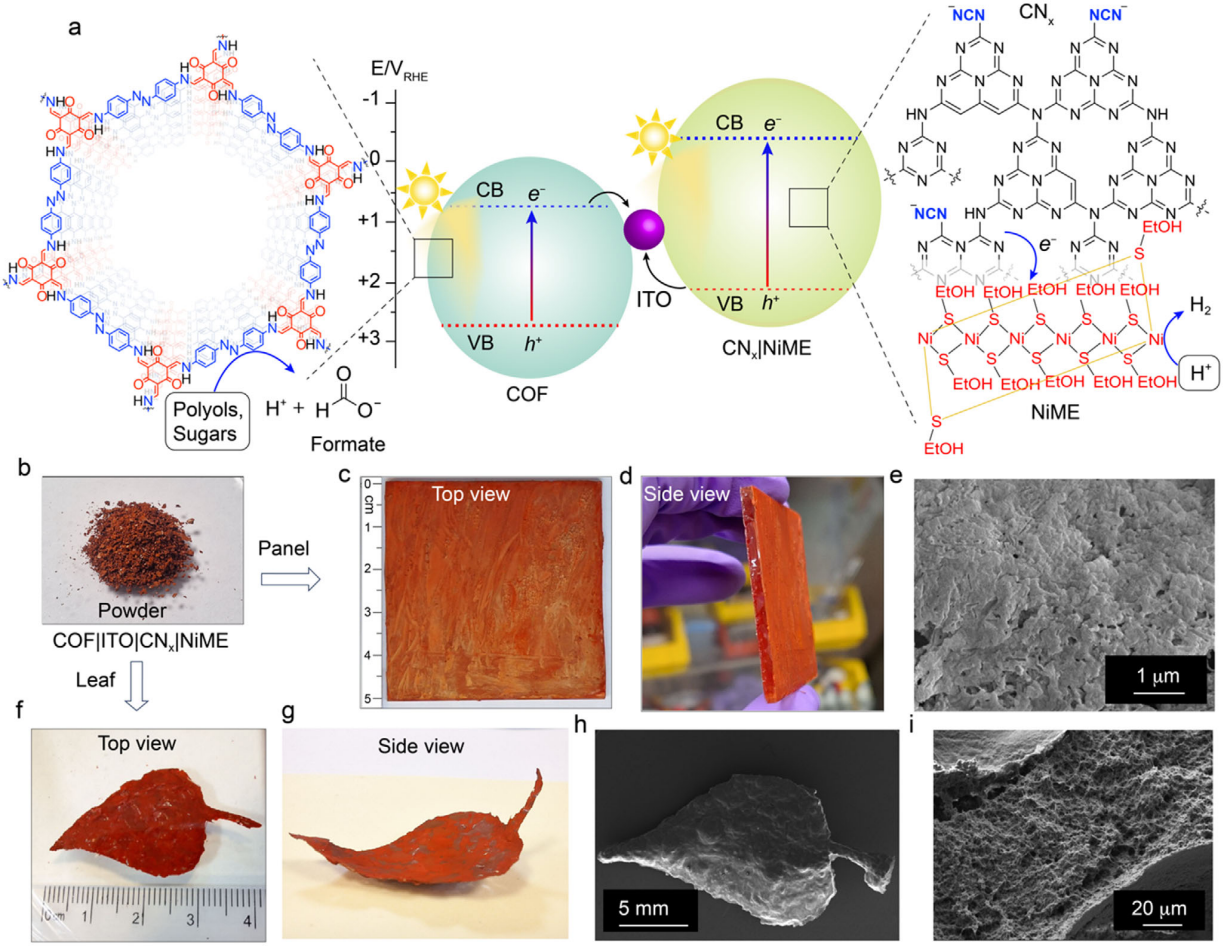
Overview of solar reforming, photopanel and photoleaf. a) Energy diagram and schematic representation of solar reforming, where H_2_ evolution has been coupled with the oxidation of polyols (ethylene glycol and glycerol) and sugars (glucose, fructose, galactose, arabinose). Oxidation of organics to HCOO^−^ occurs on the COF surface, whereas the reduction reaction (H_2_ evolution) occurs on the NiME cocatalyst which is integrated within the composite. The CB and VB potentials for CN_x_ are positioned at −0.49 and +2.15 V vs the reversible hydrogen electrode (RHE) at pH 7.0, respectively; whereas the CB and VB potentials for COF are located at +0.83 and +2.78 V vs RHE at pH 7.0, respectively. b) Powder form of the COF|ITO|CN_x_|NiME composite. c,d) Scalable (25 cm^2^) demonstration of glass‐supported COF|ITO|CN_x_ photopanel. e) Scanning electron microscopy image of the surface of the COF|ITO|CN_x_ photopanel. f,g) Digital photographs of a standalone photoleaf with the composition of COF|ITO|CN_x_. h and i) Scanning electron microscopy images of the photoleaf's top and side, respectively.

Here we present a crystalline, hierarchically porous Z‐scheme composite for scalable solar reforming in water yielding H_2_ and selective formate (HCOO^−^) from polyols and sugars (**Figure** **1**a). The system is rationally assembled by integrating (i) cyanamide‐functionalized carbon nitride (CN_x_) coupled to a nickel‐based molecular cocatalyst (NiME) for light‐driven proton reduction, (ii) indium tin oxide (ITO) nanoparticles serving as a solid‐state electron mediator to bridge the redox interfaces, and (iii) a *β*‐ketoenamine‐linked covalent organic framework (COF) as a redox‐active, nanoconfined oxidation platform for selective conversion of organics. The modularity of this platform allows its fabrication into free‐standing photoleaf (≈4 cm in length) and supported photopanel (25 cm^2^), preserving long‐range order and porosity (Figure 1b–i). A multi‐window reactor design (≈55 cm^2^ photoactive area) achieves areal H_2_ and HCOO^−^ production rates of 272.1 ± 23.8 and 137.2 ± 12.4 µmol m^−2^ h^−1^, respectively. The composite exhibits continuous operability for 7 days with high H_2_ evolution activity (213.8 ± 10.6 $\mu \mathrm{mol}\;g_{\mathrm{cat}}^{-1}\;h^{-1}$) with >90% selectivity toward HCOO^−^ demonstrating its long‐term durability. A photopanel has also been tested with pretreated polyethylene terephthalate (PET) plastic for H_2_ and HCOO^−^ generation under natural sunlight, highlighting its potential for decentralized plastic upcycling. Our results demonstrate a highly processable and durable organic Z‐scheme system that leverages polymeric material and molecular interface design to address key challenges in sustainable solar fuel generation and chemical recycling.

## 2 Results and Discussion

### 2.1 Design of Composite Photocatalyst

In order to construct a solar reforming system for H_2_ production coupled with selective oxidation of waste, we prepared a Z‐scheme composite, COF|ITO|CN_x_|NiME. The construction was made by the integration of COF, ITO nanoparticles, CN_x_, and NiME. In the proposed Z‐scheme configuration, we can leverage the selective oxidation on the COF surface along with the benefits from its hierarchical porosity and processability enabled by a unique synthesis protocol. The conduction band electrons of CN_x_ facilitate proton reduction through the NiME cocatalyst. The robustness of NiME and its operability at pH 7 enable the execution of solar reforming in pH neutral water without the use of precious metal co‐catalysts.

CN_x_ is a well‐established semiconducting light absorber known for facilitating the hydrogen evolution reaction (HER) in the presence of various molecular catalysts.^[^ ^8^ ^]^ Meanwhile, ITO nanoparticles serve as solid‐state electron mediators, relaying charges between the COF and CN_x_ while exhibiting high optical transparency.^[^ ^9^ ^]^ The composite also incorporates a known 3d transition metal HER co‐catalyst, hexanuclear Ni‐based complex (NiME), where each Ni center is coordinated to two neighboring Ni atoms and four S atoms from the *2*‐mercaptoethanol (ME) ligands (Figure S1, Supporting Information).^[^ ^10^ ^]^ This cocatalyst is driven by photoexcited CN_x_ and operates under pH neutral aqueous conditions (pH 7). The NiME's robust molecular architecture imparts long‐term chemical durability to the Z‐scheme composite under photocatalytic reaction conditions.

**Figure 2:**
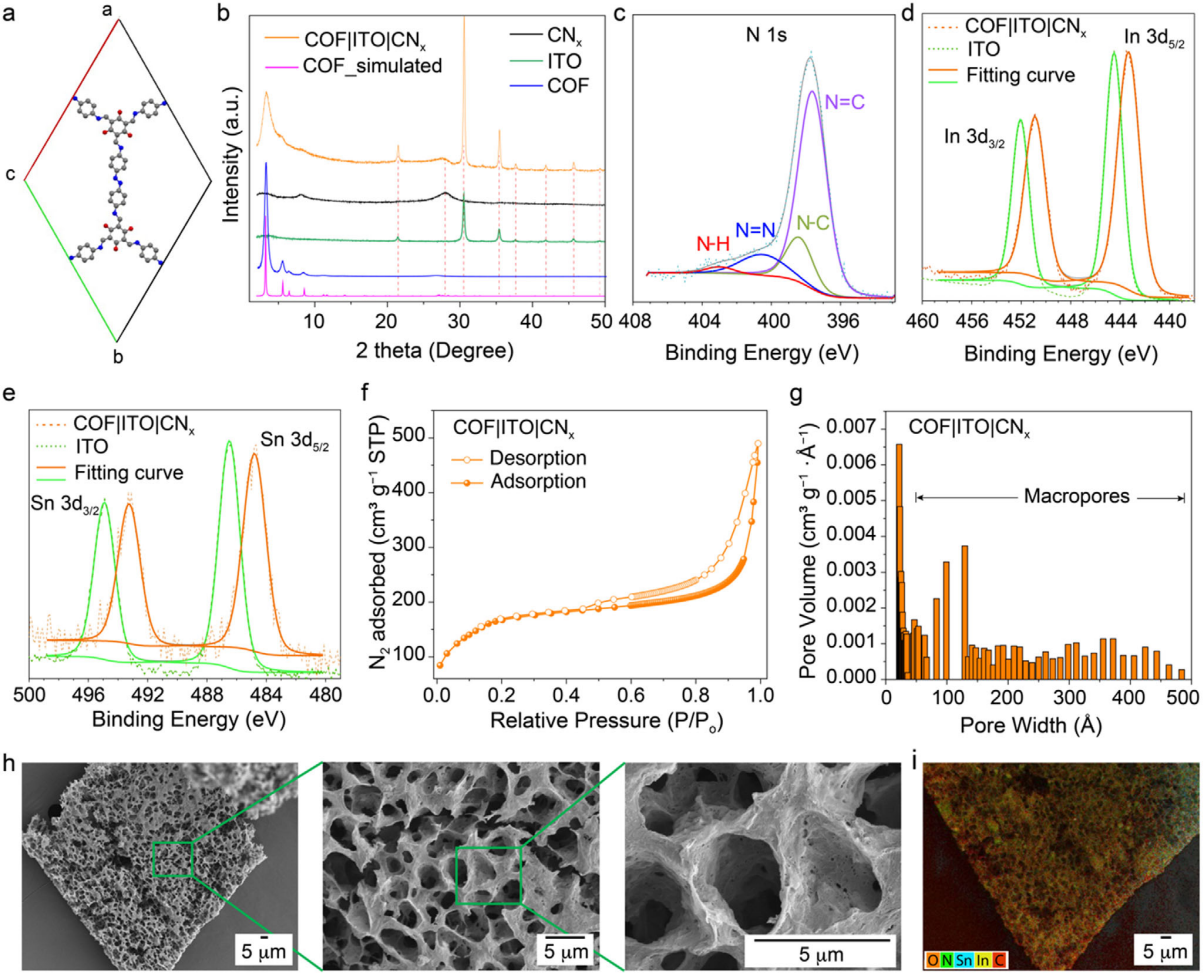
Characterization of COF|ITO|CN_x_ composite. a) Unit cell structure of the COF highlighting the secondary building unit. b) Comparison of powder X‐ray diffraction patterns of various components of the COF|ITO|CN_x_ composite. c–e) X‐ray photoelectron spectra of N 1s, In 3d_3/2 and 5/2_ Sn 3d_3/2 and 5/2_ edges for the COF|ITO|CN_x_. N 1s confirms different types of bonds within the composite. Shifts in binding energy of In 3d_3/2 and 5/2_ Sn 3d_3/2 and 5/2_ ascertain the impregnation of the ITO nanoparticles within. f) N_2_ adsorption isotherm at 77.5 K of the composite highlights the combination of type II and IV isotherms. g) Pore size distribution of the composite demonstrating various nanopores resulting hierarchically porous structure. h) Top‐view SEM images of COF|ITO|CN_x_ exhibiting the macropores. i) SEM–EDX elemental mapping image highlights the composition and uniform distribution of C, N, O, In, and Sn throughout the composite.

Complementarily, COFs are constructed through the precise integration of organic building blocks and offer ample tunability in pore functionalities that directly influence their catalytic behavior (Figure 1a).^[^ ^11^ ^]^ The azo‐functionalized (─N═N─) COF has been selected for its excellent light‐harvesting capabilities, absorbing across a broad spectral range (350–700 nm) from ultraviolet to visible light (**Figure** **2**a; S2, Discussion S1, Supporting Information).^[^ ^12^ ^]^ In contrast to conventional imine‐linked frameworks, the *β*‐keto‐enamine linkage present in the selected COF imparts exceptional chemical stability which is a critical factor under photocatalytic conditions (Figure S3, Supporting Information). The COF's nanoporous architecture facilitates adsorption and conversion of the substrates and promotes selective product formation via nanoconfinement.^[^ ^13^ ^]^ The high surface area and hierarchical porosity could enhance catalytic performance by providing abundant active sites and improving mass transport.^[^ ^14^ ^]^ The valence band edge of the COF is suitably positioned to drive oxidation reactions of organics. Additionally, the unique synthesis process of the COF enables processability into practical composite forms such as photopanels and photoleaves.

### 2.2 Synthesis and Characterization

CN_x_ was synthesized following a previous literature protocol (see Experimental Section).^[^ ^15^ ^]^ The COF|ITO|CN_x_ composite was synthesized via solid‐state synthesis (see Experimental Section).^[^ ^16^ ^]^ Initially, *p*‐toluene sulphonic acid (250 mg, 1.3 mmol) and *4,4´*‐azo dianiline (48 mg, 0.23 mmol) were mixed thoroughly with a mortar and pestle, followed by the addition of *2,4,6*‐triformylphloroglucinol (31.5 mg, 0.15 mmol) to the mixture. The grinding resulted in an orange powder. Subsequent dropwise addition of 30 µL water transformed the powder into dark orange paste. Then 72.4 mg of CN_x_ (to give ≈1:1 weight ratio of COF and CN_x_ in final composition) was added and the mixture was further ground with occasional additions of water (30 µL each time, 2–3 times) until a homogeneous paste was formed (see Table S1; Discussion S2, Supporting Information). Next, 36.2 mg ITO nanoparticle was added (for 1:0.5 = COF:ITO weight ratio formation in final composition, see Discussion S2, Supporting Information) and grinding was continued with an additional 60 µL of water. To introduce hierarchical porosity within the composite, the resulting mixture was freeze‐dried (12–14 h) prior to thermal annealing at 90 °C for 20 h (Discussion S3, Supporting Information). The solid was then sequentially washed with water, *N,N*‐dimethyl acetamide (DMAc) and acetone and subsequently dried under vacuum to result in the COF|ITO|CN_x_ composite (COF:ITO:CN_x_ = 1:0.5:1, integrated together unlike a physical mixture) with hierarchical porous surface. The final composite, COF|ITO|CN_x_|NiME, was obtained by integrating NiME through adsorption within the nanoporous COF|ITO|CN_x_ matrix. To achieve this, 1 mL 1.25 µm aqueous NiME solution was treated with 2 mg of COF|ITO|CN_x_ composite followed by ultrasonication for 20 min and stirring for an additional 2 h at 600 rpm. The resulting solid was thoroughly washed with water (3×) and dried under vacuum at 50 °C overnight, resulting in the COF|ITO|CN_x_|NiME composite.

The powder X‐ray diffraction (PXRD) pattern of the composite matches with the patterns of its individual components, thereby confirming their presence with phase purity (Figure 2b; Figure S4a, supporting information). The distinct peak at 3.3° corresponds to the COF's (100) facet whereas the broad peak at 27.5° is due to the two‐dimensional CN_x_ (Figure 2b: black vs orange trace). Further analysis showcases that the CN_x_ is intercalated between two layers of COF or sandwiched between multiple COF layers (Figures S4b,S5, Discussion S4, Supporting Information). Other characteristic peaks of the composite are visible at 5.5 and 8.3° that correspond to COF's (2−10) and (200) facets, respectively. The characteristic peaks at 21.5, 30.6, 35.4, 37.7, 41.8, 45.6, and 49.3° confirm the presence of ITO nanoparticles.^[^ ^17^ ^]^ The comparison of PXRD patterns (before and after the integration within composite) confirm that ITO nanoparticles’ original phase purity remains intact (Figure 2b: green vs orange trace).

High‐resolution transmission electron microscopy (HRTEM) images show that the COF|ITO|CN_x_ composite has moderate crystallinity with certain periodicities (Figure S6, Supporting Information). The lattice fringe distance of 0.45 nm corresponds to the interlayer spacing, which is larger than that of pristine COF (Figure S6a,b, Supporting Information). This increase is attributed to the intercalation of the CN_x_ layer between COF layers, resulting in expanded interlayer spacing, as further confirmed by PXRD. The lattice fringe distance of 2.6 nm corresponds well to the interplane distance between the (2−10) planes, corroborating the pore diameter of the COF (Figure S6c–e, Supporting Information).

The comparative analysis of the attenuated total reflection infrared (ATR‐IR) spectra for the individual components confirms their successful integration within the composite (Figure S7a,b, Supporting Information). Characteristic vibration bands at 1618 (─C═O), 1570 (─C═C─), and 1277 cm^−1^ (─C─N─) are indicative of the *β*‐keto‐enamine backbone, confirming COF network formation.^[^ ^16^ ^]^ For CN_x_, signature peaks are observed at 2167 cm^−1^ (C═N stretch, cyanamide functionality), 1223 and 1311 cm^−1^ (secondary amine ─C─N bending), and at 808 cm^−1^ (heptazine core).^[^ ^8a^ ^]^ The high‐resolution N 1s X‐ray photoelectron spectra (XPS) of the composite represent all the chemical bonds (N−) present therein and they match well with the literature (Figure 2c).^[^ ^8a^ ^]^ Slight shifts in In 3d_3/2_, In 3d_5/2_, Sn 3d_3/2_ and Sn 3d_5/2_ peak positions confirm the chemical integration of ITO nanoparticles within the composite unlike a physical mixture (Figure 2d,e).

To further confirm atomic‐level interactions between COF and CN_x_, XPS analysis was performed comparing the N 1s spectra of COF, CN_x_, and the COF|CN_x_ composite. The N 1s spectrum of COF|CN_x_ exhibits two peaks at 397.5 and 397.1 eV, shifted from 397.8 eV in COF and 396.7 eV in CN_x_, respectively (Figure S7c, Supporting Information). This spectral shift supports the strong electronic coupling between COF and CN_x_ layers rather than simple physical adsorption or phase separation.

Thermogravimetric analysis (TGA) confirms the COF|ITO|CN_x_ composite's high thermal stability up to 450 °C (Figure S8, Supporting Information). Elemental analysis (CHN) of the COF|ITO|CN_x_ composite (COF:ITO:CN = 1:0.5:1 by weight) gave C 37.49%, H 2.73%, and N 21.92% (Table S2, Supporting Information). These values are close to those expected from the component CHN values (C 36.48%, H 2.04%, N 23.42%), with the small deviations likely arising from residual *N,N*‐dimethylacetamide retained within the pores and minor loss of ITO during washing.

The nitrogen adsorption isotherm of the COF|ITO|CN_x_ composite at 77.5 K indicates combination of type II and IV isotherms (Figure 2f). Type II nature confirms the macroporous structure, whereas Type IV is characteristic of a mesoporous material. The presence of a hysteresis loop within the isotherm also supports the mesoporous nature of the material. However, the absence of hysteresis at the low‐pressure region hints at the composite's uniform microporosity. The composite showcases high Brunauer–Emmett–Teller (BET) surface area of 555 m^2^ g^−1^ and pore volume of 0.75 cm^3^ g^−1^, respectively (Table S3, Supporting Information). The decrease in overall surface area as compared to the pristine COF could be due to the pore blockage by the ITO nanoparticles (Figures S9 and S10, Supporting Information). The pore size measured with non‐linear density function theory (NLDFT) suggests various sizes of pores ranging from 2.2 to 48 nm confirming the presence mesopores (Figure 2g).

Diverse pores were generated due to the predesigned COF structure, integration of two‐dimensional CN_x_ and applying freeze drying.^[^ ^18^ ^]^ The T‐plot report provides compelling evidence of hierarchical porosity and surface area (Figure S11, Table S4, and Discussion S5, Supporting Information). The COF|ITO|CN_x_ possesses microporous volume and an area of 0.023 cm^3^ g^−1^ and 17.5 m^2^ g^−1^, respectively. The high external surface area of 537.5 m^2^ g^−1^ is due to the mesopores and macropores, that further indicates a hierarchical pore structure. The macroporosity of the composite was ascertained by the scanning electron microscopy (SEM) (Figure 2h; Figure S12, Supporting Information). This macroporous surface morphology is crucial for continuous mass flow within the system, which maximizes solar driven H_2_ production (*see next section*). The top‐down energy‐dispersive X‐ray spectroscopy (EDX) confirms the presence of all the elements; C, N, O, In, and Sn and their uniform distribution throughout the COF|ITO|CN_x_ composite (Figure 2i; Figure S13, Supporting Information). NiME has a diameter of 14.59 Å, which makes it suitable to fit inside the mesopores of the composite resulting in the COF|ITO|CN_x_|NiME composite (Figure S1, Supporting Information). This immobilization could be attributed to the EtO–H···O hydrogen bonding between the *2*‐mercaptoethanol and the keto‐functional group (─C═O) of the COF backbone.^[^ ^13a^ ^]^ The particle size of the COF|ITO|CN_x_|NiME composite powder employed during catalysis ranged from 5 to 30 µm (Figure S14a–c, Supporting Information). However, the detection of Ni content within the COF|ITO|CN_x_|NiME composite via XPS was hindered due to the low Ni loading (0.43 wt.%, see below) and limited surface exposure (Figure S14d–f, Supporting Information).

The successful integration of CN_x_ and COF within the COF|ITO|CN_x_ composite enables absorption across a broad spectral range, from the UV region to visible light (up to ≈650 nm) (Figure S15a, Supporting Information). To validate the possibility of a Z‐scheme electron transfer pathway, the band structures of both semiconductors were determined using UV‐vis and ultraviolet photoelectron spectroscopy (UPS) (Figure S15b–i, Supporting Information). COF and CN_x_ exhibited the band gaps of 1.95 and 2.65 eV, respectively, in agreement with previous literature reports.^[^ ^8^, ^12^ ^]^ The conduction band minima (CBM) for CN_x_ and COF were measured to be at −0.49 and +0.83  V (vs RHE, pH 7.0), respectively, while their valence band maxima (VBM) were at +2.15 and +2.78 V (vs RHE, pH 7.0), respectively (Figures 1a; Table S5, Supporting Information).

### 2.3 Solar Reforming Activity

Under standard solar reforming condition (unless stated otherwise), the COF|ITO|CN_x_|NiME composite (2 mg mL^−1^, COF:ITO:CN_x_:NiME = 25.4:12.7:25.4:1 weight ratio and NiME loading = 24.2 nmol) was employed in water (pH≈7, 25 °C) with a substrate concentration of 0.1 m under simulated solar irradiation (AM 1.5G, 100 mW cm^−2^) and constant stirring at 600 rpm. The pH of the solution remained close to 7 after the catalysis (24 h reaction and even long‐duration reaction of 7 days).

Considering ethylene glycol (EG) as the key monomeric product from PET plastic depolymerization, EG was selected as the model substrate to optimize the solar reforming conditions (Discussion S6, Supporting Information). The macroporous surface structure of the composite proved advantageous during the catalysis as multifold enhancement (>2.5 times) of H_2_ evolution activity was observed when compared with the unmodified surface where macropores were absent (Figure S16 and Table S6, Supporting Information).

Various amounts of NiME were screened for the optimization of cocatalyst's concentration. Pre‐treatment of 2 mg COF|ITO|CN_x_ composite with 1.25 µm NiME and 0.1 m EG in a total of 1 mL aqueous solution yielded the highest catalytic activity (Figure S17 and Table S7, Supporting Information). The NiME loading under this condition corresponds to 12.1 nmol per mg of COF|ITO|CN_x_ composite (COF:ITO:CN_x_:NiME = 25.4:12.7:25.4:1, weight ratio) which was measured via inductively coupled plasma optical emission spectrometry (ICP‐OES) (Table S8, Supporting Information). The reduced performance at lower NiME concentrations was attributed to insufficient active centers for electron extraction, whereas excessive NiME loading led to significant performance losses, likely due to surface coverage that hinders substrate access and increases parasitic light absorption by the cocatalyst (Figures S18–S20, Supporting Information).

**Figure 3:**
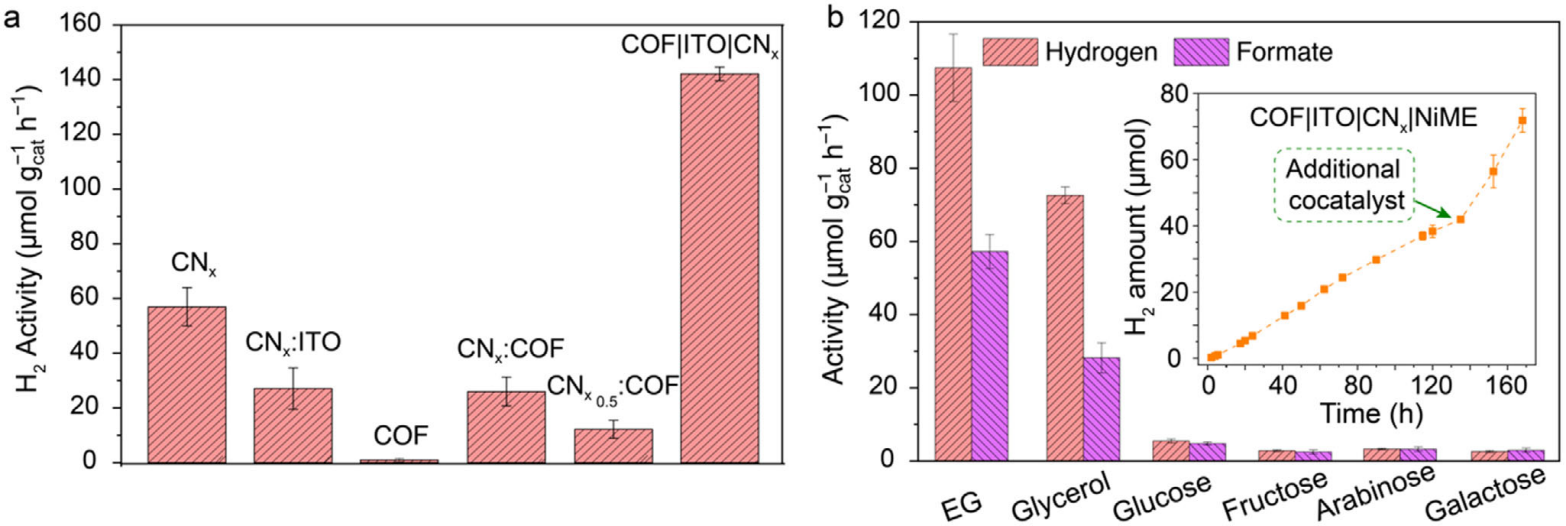
Solar reforming activity. a) Control experiments during photocatalytic H_2_ evolution varying components and ratios (conditions: NiME = 1.25 µm, H_2_O as solvent, 0.2 m EG for 21 h). b) Bar plots showing the H_2_ and HCOO^−^ activity during solar reforming with COF|ITO|CN_x_|NiME using 0.1 m polyols and sugars; inset: long‐term solar reforming of EG (7 days). Activity of H_2_ production was measured at different time intervals with reaction 0.5 m EG. Additional NiME cocatalyst was added at 132^nd^ h of the reaction (Discussion S7, Supporting Information). All the measurements were executed with 2 mg mL^−1^ of photocatalyst, 600 rpm stirring and irradiation (AM 1.5 G, 100 mW cm^−2^, 25 °C).

In order to understand the role of each component, further control experiments were performed. 2 mg of different types of photocatalysts (for mixtures, based on the total mass) were first treated with with 1.25 µm NiME and 0.1 m EG in a total of 1 mL aqueous solution. The bare COF is ineffective toward H_2_ evolution, whereas CN_x_ exhibits an activity of 56.8 ± 6.9 µmol H_2_ g_cat_^−1^ h^−1^ during EG oxidation (**Figure** **3**a; Table S9, Supporting Information). A 1:1 mixture of ITO and CN_x_ showcased a substantially lower activity (27 ± 7.5 µmol H_2_ g_cat_^−1^ h^−1^), approximately half that of pristine CN_x_. A similar trend was observed for the COF:CN_x_ (1:1) mixture, where catalytic activity arose solely from the CN_x_ component (25.9 ± 5.3 µmol H_2_ g_cat_^−1^ h^−1^). Decreasing the CN_x_ content (COF:CN_x_ = 2:1) led to a further decline in performance (12.2 ± 3.3 µmol H_2_ g_cat_^−1^ h^−1^).

In contrast, integration of ITO to construct the COF|ITO|CN_x_ composite resulted in a substantial enhancement (≈2.5‐fold) in H_2_ evolution activity (141.7 ± 2.5 µmol H_2_ g_cat_^−1^ h^−1^) compared to the COF:CN_x_ (1:1) mixture, highlighting the pivotal role of ITO in facilitating the solid‐state electron relay process. Multi‐walled carbon nanotube (MWCNT) has also been tested as a solid‐state electron mediator to construct the Z‐scheme system.^[^ ^19^ ^]^ Although COF|MWCNT|CN_x_|NiME composite (COF:MWCNT:CN_x_ = 1:0.2:1) exhibited solar‐driven H_2_ generation, a significant reduction in efficiency (61.8 µmol H_2_ g_cat_^−1^ h^−1^) is likely due to the black coloration of MWCNT that decreases the overall light absorption of the COF|MWCNT|CN_x_|NiME composite (Table S10: entry 1, Supporting Information). The ITO content within COF|ITO|CN_x_ composite was also systematically optimized where either increasing or decreasing the ITO content from optimal COF to ITO of 1:0.5 led to the decrease in performance (Table S11: entries 1 and 2, Supporting Information).

Increasing the content of either COF or CN_x_ beyond the optimized level of COF:CN_x_ = 1:1 ratio within COF|ITO|CN_x_ composite led to reduced H_2_ evolution (Table S11: entries 3 and 4, Supporting Information). Exclusion control experiments confirmed that no detectable H_2_ was produced in the absence of any one of the following: substrate (EG), photocatalyst (COF|ITO|CN_x_), cocatalyst (NiME) and light (Table S10: entries 2–6 Supporting Information). The external quantum efficiency (EQE) for HER with monochromatic light (λ = 400 nm and intensity of 6.3 mW cm^−2^) was calculated to be 0.03% and 0.1% for CN_x_|NiME and COF|ITO|CN_x_|NiME, respectively (Figure S21 and Table S12, Supporting Information). This further ascertained the active participation of COF during solar reforming.

The time‐dependent H_2_ evolution with concomitant EG oxidation using COF|ITO|CN_x_|NiME composite confirms high activity (169.2 ± 3.5 µmol g_cat_^−1^ h^−1^, 24.3 ± 0.1 µmol H_2_) after 72 h of solar reforming (Figure 3b inset; Table S13, Supporting Information). Although the bare NiME cocatalyst decomposes in water after 72 h of irradiation, confinement of the NiME complex inside the nanopores of the composite COF|ITO|CN_x_ is suggested to stabilize the NiME from fragmentation (Figures S22 and S23, Supporting Information). However, the possibility of formation of nickel oxides (NiO_x_) cannot be completely ruled out by partial degradation of NiME, which could further participate in the oxidation and or reduction process. Addition of fresh NiME after 5 days of reaction resulted in significant enhancement of H_2_ production (0.897 µmol h^−1^), which may be due to additional activity of the fresh and initial co‐catalyst (Discussion S8, Supporting Information). After 7 days of solar reforming reaction, a maximum H_2_ activity of 213.8 ± 10.6 µmol g_cat_^−1^ h^−1^ (71.8 ± 3.5 µmol H_2_) was obtained (Figure 3b inset; Table S13, Supporting Information). This showcases the long‐term durability of the composite that can continuously produce H_2_ (71.8 ± 3.5 µmol) and HCOO^−^ (34.8 ± 4.8 µmol) (Tables S13 and S14, Supporting Information).

Sugars (glucose, fructose, arabinose, and galactose) and glycerol which are part of different waste streams have also been used as reductants. A considerable amount of H_2_ was observed during this process along with HCOO^−^ as the main oxidation product with >90% selectivity; glucose: 5.5 ± 0.6 µmol H_2_ g_cat_^−1^ h^−1^ and 4.8 ± 0.4 µmol HCOO^−^ g_cat_^−1^ h^−1^, fructose: 2.9 ± 0.2 µmol H_2_ g_cat_^−1^ h^−1^ and 2.5 ± 0.5 µmol HCOO^−^ g_cat_^−1^ h^−1^, arabinose: 3.3 ± 0.2 µmol H_2_ g_cat_^−1^ h^−1^ and 3.3 ± 0.6 µmol HCOO^−^ g_cat_^−1^ h^−1^, galactose: 2.6 ± 0.3 µmol H_2_ g_cat_^−1^ h^−1^ and 2.9 ± 0.6 µmol HCOO^−^ g_cat_^−1^ h^−1^, and glycerol: 72.6 ± 2.2 µmol H_2_ g_cat_^−1^ h^−1^ and 28.2 ± 4.1 µmol HCOO^−^ g_cat_^−1^ h^−1^ (Figure 3b; Table S15, Supporting Information). The selectivity of the oxidation product was calculated based on the expected HCOO^−^ generation per H_2_. Proton reduction to H_2_ is a 2‐e^−^ transfer process, wheras oxidation of EG to HCOO^−^ involves a 6‐e^−^ transfer and produces 2 HCOO^−^ units. Thus, the expected H_2_ to HCOO^−^ molar ratio is 3:2. The large size of the polymeric sugars compared to the polyols hinders effective mass transfer process during the oxidation reaction, thereby affecting the overall H_2_ production and their ratio of H_2_ to HCOO^−^ (selectivity) during the solar reforming process.

The composite powder has also been recycled for 6 cycles using EG as substrate without compromising the H_2_ production activity (Figure S24, Supporting Information). The composite's chemical and physical structure remain unaltered after the catalysis. The PXRD pattern after the reaction clearly shows that the long‐range order of the composite is intact (Figure S25a, Supporting Information). XPS also confirms that the surface properties and bond connectivity of the composite remain unchanged even after recycling the composite for multiple cycles (Figure S25b–f, Supporting Information).

### 2.4 Mechanism of Solar Reforming

**Figure 4:**
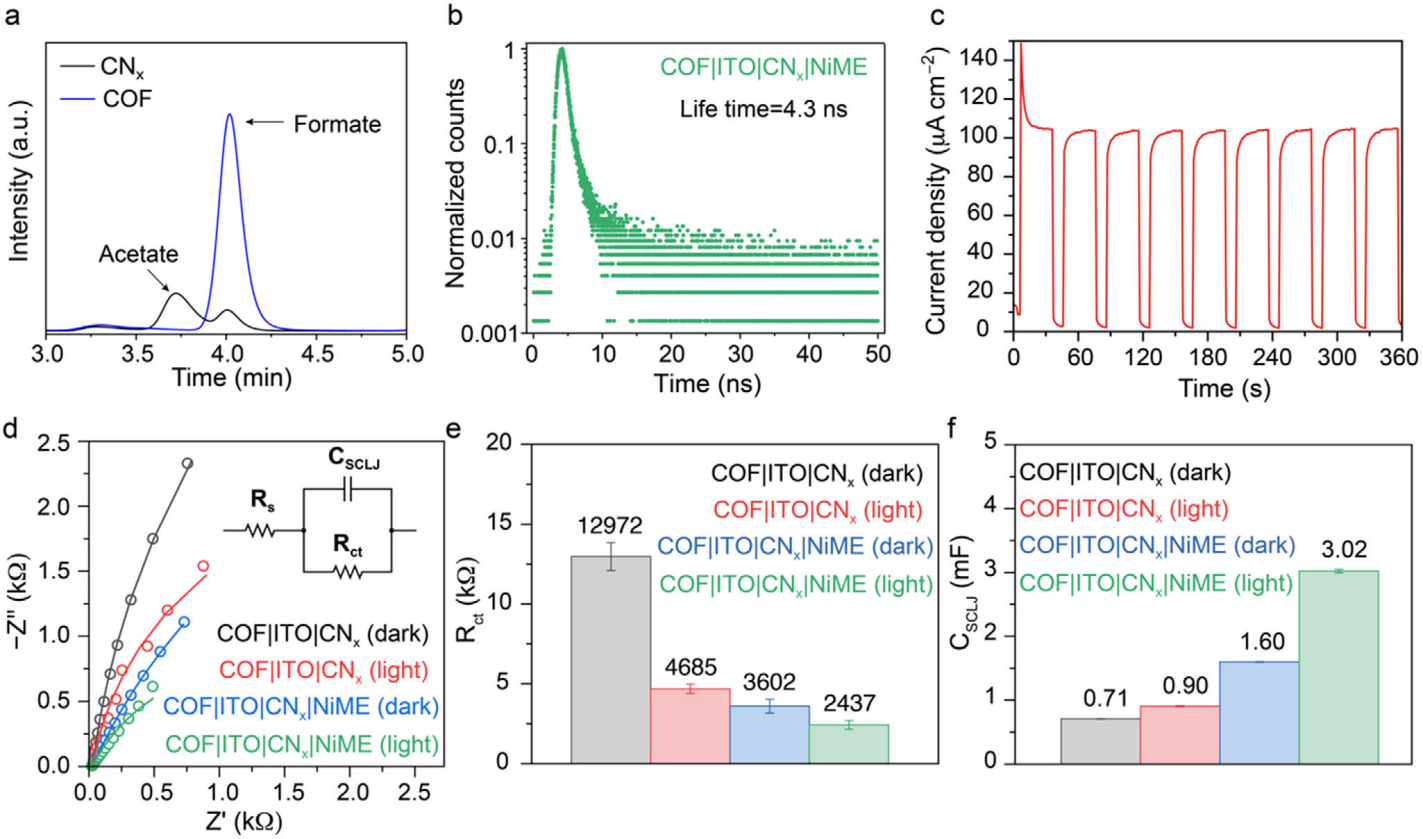
Mechanistic investigation of solar reforming. a) Comparison of oxidation product selectivity with CN_x_ and COF using AgNO_3_ as electron acceptor during photocatalysis analyzed by ion chromatography. Conditions: photocatalyst (2 mg mL^−1^), H_2_O as solvent, 0.1 m EG, 17.7 mm AgNO_3_, 600 rpm stirring and irradiation (21 h, AM 1.5 G, 100 mW cm^−2^, 25 °C). b) Excited state lifetime measurement through time‐correlated single photon counting of COF|ITO|CN_x_|NiME composite. c) Chronoamperometry (CA) of the COF|ITO|CN_x_ photoelectrode at 1.0 V vs RHE. d) Nyquist plot of impedance response recorded under reducing conditions at −0.1 V vs RHE (open circles) with corresponding fitting curves (solid lines); inset: equivalent circuit for fitting. Conditions: water (pH 7) containing 0.1 m EG, Ag/AgCl (sat. NaCl) reference electrode, Pt mesh counter electrode in 0.5 m Na_2_SO_4_ (pH 7), AM 1.5G irradiation, 25 °C. e,f) Quantitative analysis on impedance response: R_ct_ and C_SCLJ_ refer to charge transfer resistance and semiconductor–liquid junction capacitance, respectively. Error bars originate from standard error of nonlinear regression.

To elucidate the working mechanism of the composite, we conducted experiments of the oxidative half‐reaction using AgNO_3_ as a sacrificial electron acceptor, with COF serving as both the light absorber and catalyst during light‐driven EG oxidation. Remarkably, the result revealed that EG was converted to HCOO^−^ with ≈99% selectivity as no other product was observed during this control half‐reaction. In contrast, CN_x_ with AgNO_3_ resulted in acetate (CH_3_COO^−^) and HCOO^−^ as products during solar reforming at neutral pH (**Figure** **4**a). The high selectivity of the COF could be due to its unique porous structure, where oxidation reaction can proceed within the nanoconfined cavity offered by the COF. This selective oxidation behavior of the COF was further extended to solar reforming, where oxidation product analysis demonstrated that the COF|ITO|CN_x_|NiME composite produced HCOO^−^ with > 90% selectivity (Figure 3b). In contrast, CN_x_ typically yields a broader distribution of byproducts, including glycolaldehyde, glyoxal, glycolate, glyoxylate, formate, and acetate (Figure S26, Supporting Information).^[^ ^3^, ^15^ ^]^ These findings indicate that the oxidation reaction predominantly occurs at the COF surface, while electrons are relayed through ITO to CN_x_ to facilitate the reduction reaction via NiME during solar reforming.

Although chemical intermediates during EG oxidation to HCOO^−^ were not detected, the presence of other oxidation products have been confirmed during glucose solar reforming. The reaction medium during glucose oxidation was analyzed after 6 h of reaction by liquid chromatography–mass spectrometry (LC–MS) (Figures S27 and S28, Discussion S9, Supporting Information). The analysis highlights the formation of intermediates like *5*‐hydroxymethylfurfural and levulinic acid. Their absence at the end of the reaction hints that they were further transformed to HCOO^−^ after 24 h irradiation. This finding also supports that the COF offers unique nanoconfinement, thereby leading to the further oxidation of intermediates toward selective product formation at longer time durations. The position of the CBM rationalizes the COF's inability to independently produce H_2_. Within the COF|ITO|CN_x_|NiME composite, although ITO enhances the electrical conductivity, it mainly acts as a solid‐state electron mediator (Figure S29a,b, Discussion S10, Supporting Information). Overall, ITO enables the synergistic exploitation of COF's selective oxidation capability alongside CN_x_‐driven reduction resulting a Z‐scheme photosystem (Figure S29c, Supporting Information).^[^ ^4^, ^6^ ^]^

Control experiments using optical filters supported this mechanistic proposal. Under a 450 nm cut‐off filter, where only the COF component within the COF|ITO|CN_x_|NiME composite was selectively excited, no H_2_ generation was observed. In contrast, employing a 420 nm cut‐off filter resulted in significantly reduced activity as both COF and CN_x_ have considerable light absorption below 420 nm (Table S10: entries 7 and 8, Supporting Information). The steady‐state fluorescence spectrum confirms strong emission from the composite (Figure S30a, Supporting Information), which may be sensitive to the introduction of cocatalysts. To gain further insight into this charge transfer dynamics, we performed time‐correlated single photon counting (TCSPC) measurements. A decrease in fluorescence lifetime (5.2 → 4.3 ns) for the COF|ITO|CN_x_|NiME composite compared to the COF|ITO|CN_x_ composite indicates accelerated excited‐state electron transfer from CN_x_ to the cocatalyst NiME, providing evidence for the proposed Z‐scheme solar reforming pathway Figure (4b; Figure S30b–d, Supporting Information).

Mott–Schottky (M–S) analysis confirms both COF and CN_x_ as n‐type semiconductors, and the derived band positions further verify the Z‐scheme heterostructure of the composite (Figure S31a, Supporting Information). The photopanel functions as a photoelectrode, demonstrating a stable anodic photocurrent response of 0.1 mA cm^−2^ for EG oxidation at 1.0 V vs RHE (Figure 4c). The COF|ITO|CN_x_ composite‐based photopanel showcases both cathodic and anodic photocurrent under catalytic condition, consistent with the Z‐scheme mechanism of the heterostructure (Figure S31b, Supporting Information).^[^ ^20^ ^]^ Photoelectrochemical impedance spectroscopy (PEIS) provides additional insights into the semiconducting properties of COF|ITO|CN_x_ and highlights the role of NiME as a cocatalyst in enhancing charge transfer efficiency and promoting charge storage for subsequent catalytic HER. PEIS was conducted under *operando* conditions, specifically, under simulated AM1.5G irradiation in presence of EG using the COF|ITO|CN_x_ composite immobilized on a fluorine‐doped tin oxide (FTO)‐coated glass support (see Experimental Section).

The Nyquist plot recorded under reducing conditions at –0.1 V versus RHE (Figure 4d) exhibited a single semicircle, which was fitted using a Randles circuit (Figure 4d inset)^[^ ^21^ ^]^ comprising a series resistor (*R*_S_), a charge transfer resistor (*R*_ct_), and a capacitor to characterize the semiconductor–liquid junction (*C*_SCLJ_), thereby offering quantitative insights into photoconductivity and charge carrier dynamics under reducing conditions where photogenerated electrons flow toward the SCLJ for HER. The constant *R*_S_, ≈27.3 Ω, across four different conditions (Figure S32, Supporting Information) demonstrates the consistency of cell resistance. Variations in *R*_ct_ (Figure 4e) elucidate the photoconductivity of the COF|ITO|CN_x_ and the catalytic role of NiME for HER. Initially, under simulated 1 sun irradiation on bare COF|ITO|CN_x_, *R*_ct_ decreases significantly from 12 972 ± 873 to 4685 ± 287 Ω, indicating robust photoconductivity of COF|ITO|CN_x_ as a light absorber. Subsequently, the introduction of NiME in the COF|ITO|CN_x_|NiME composite effectively facilitates the transfer of photogenerated electrons from COF|ITO|CN_x_ to reduce protons under illumination, leading to a further decrease in *R*_ct_ from 4685 ± 287 to 2437 ± 276 Ω. Such facilitation has been extensively reported in both synthetic cocatalysts and biocatalysts.^[^ ^22^ ^]^ Capacitance analysis (Figure 4f) demonstrated an increase in photogenerated charge carriers upon light irradiation on bare COF|ITO|CN_x_, as evidenced by a rise in *C*_SCLJ_ from 0.71 to 0.90 mF. It is noteworthy that in the dark, the COF|ITO|CN_x_|NiME system exhibits an increase in *C*_SCLJ_ from 0.71 to 1.60 mF compared to bare COF|ITO|CN_x_, indicative of NiME's capacity to effectively retain charges. Upon irradiation, a further rise in *C*_SCLJ_ of COF|ITO|CN_x_|NiME from 1.60 to 3.02 mF was observed, confirming NIME's capability to extract and store charges from COF|ITO|CN_x_ for HER.

### 2.5 Processability and Scalability of Photocatalysts

The unique synthetic methodology offers shapability of the photocatalysts and allows the construction of its standalone form without support structure. The COF|ITO|CN_x_ composite was shaped into a standalone photoleaf (Figure 1f,g; Figure S33, Supporting Information) following a similar protocol to that used for the synthesis of the powder form of the composite. First, a homogeneous paste was prepared by sequentially mixing *p*‐toluenesulfonic acid, *4,4′*‐azodianiline, *2,4,6*‐triformylphloroglucinol, CN_x_, water (100 µL) and ITO nanoparticles (see Experimental Section). This mixture was cast onto a kapton tape templated glass substrate, targeting a composite loading of 2 mg cm^−2^, with the thickness maintained at ≈200 µm. The cast glass was placed in a vacuum desiccator containing 100 µL water to control humidity and prevent cracking during solvent evaporation, and subsequently heated at 60 °C for four days under vacuum.^[^ ^16^ ^]^ The resulting COF|ITO|CN_x_ photoleaf (with COF:ITO:CN_x_ = 1:0.25:1) is then taken off from the glass surface and thoroughly washed with water, DMAc and acetone, followed by drying under vacuum at 50 °C.

PXRD analysis of the COF|ITO|CN_x_ photoleaf confirmed the preservation of long‐range order for both the COF and ITO phases, with CN_x_ homogeneously embedded within the matrix (Figure S34, Supporting Information). The peak positions matched with the powder form of COF|ITO|CN_x_ composite, suggesting a stacked and intercalated layered structure of the photoleaf (Figure S5 and Discussion S4, Supporting Information). Nitrogen sorption measurements revealed a high BET surface area of 315 m^2^ g^−1^, with T‐plot analysis attributing 257 m^2^ g^−1^ to meso‐ and macro‐porosity (Figure S35, Tables S3 and S4, Supporting Information). Pore size distribution analysis by NLDFT further confirmed the presence of hierarchical meso‐ to macro‐pores (Figure S36, Supporting Information). Cross‐sectional SEM imaging determined the photoleaf thickness to be ≈200 µm (Figure S37a, Supporting Information), with macroporous morphology visible in the inner regions (Figure 1h,i; Figure  S37b–d, Supporting Information), and elemental mapping by EDX confirmed uniform composition comparable to the powder material (Figure S38, Supporting Information).

To further demonstrate the scalability of the process, COF|ITO|CN_x_ was cast on a 5 × 5 cm^2^ frosted glass substrate to form a photopanel (Figure 1c,d; Figure S39, Supporting Information). Following a similar fabrication procedure of the standalone photoleaf as mentioned above, the paste was spread with a doctor blade to achieve a targeted COF|ITO|CN_x_ composite loading of 0.5 mg cm^−2^, controlled by pressing with a second non‐frosted glass slide. The assembly was flash‐frozen with liquid nitrogen, freeze‐dried for 12 h, and subsequently heated at 60 °C for three days. The COF|ITO|CN_x_ photopanel was then washed sequentially with water, DMAc, acetone, and water before use. The resulted photopanel retained high crystallinity and uniform elemental composition across the panel (Figures S40–S42, Supporting Information).

### 2.6 Solar Reforming with Processed Composite

The COF|ITO|CN_x_|NiME photoleaf and photopanel (see Experimental Section for NiME loading procedures) were evaluated for solar reforming using EG as electron donor in a top‐down photoreactor and a 3D‐printed single‐window reactor, respectively (Figures S43 and S44, Supporting Information). The photopanel exhibited higher activity (56.1 µmol H_2_ g_cat_^−1^ h^−1^), attributed to its lower composite thickness compared to the standalone photoleaf (34.9 µmol H_2_ g_cat_^−1^ h^−1^), where a higher mass‐to‐surface ratio limits the overall performance (Figures S45 and S46, Table S16, and Discussion S11, Supporting Information). In order to understand the dependence of solar reforming activity on composite's thickness, a series of COF|ITO|CN_x_ composite films was synthesized by controlled drop‐casting. SEM analysis revealed films with thicknesses from 42 ± 2.8 to 301.6 ± 19.7 µm (Figure S47, Supporting Information). As expected, solar reforming tests with COF|ITO|CN_x_|NiME films show that the specific H_2_ evolution activity decreases with increasing film thickness. The thinnest film exhibited the highest specific activity of 88 µmol H_2_ g_cat_^−1^ h^−1^, whereas the thickest film showed only 17.1 µmol H_2_ g_cat_^−1^ h^−1^ (Figure S48, Supporting Information).

**Figure 5:**
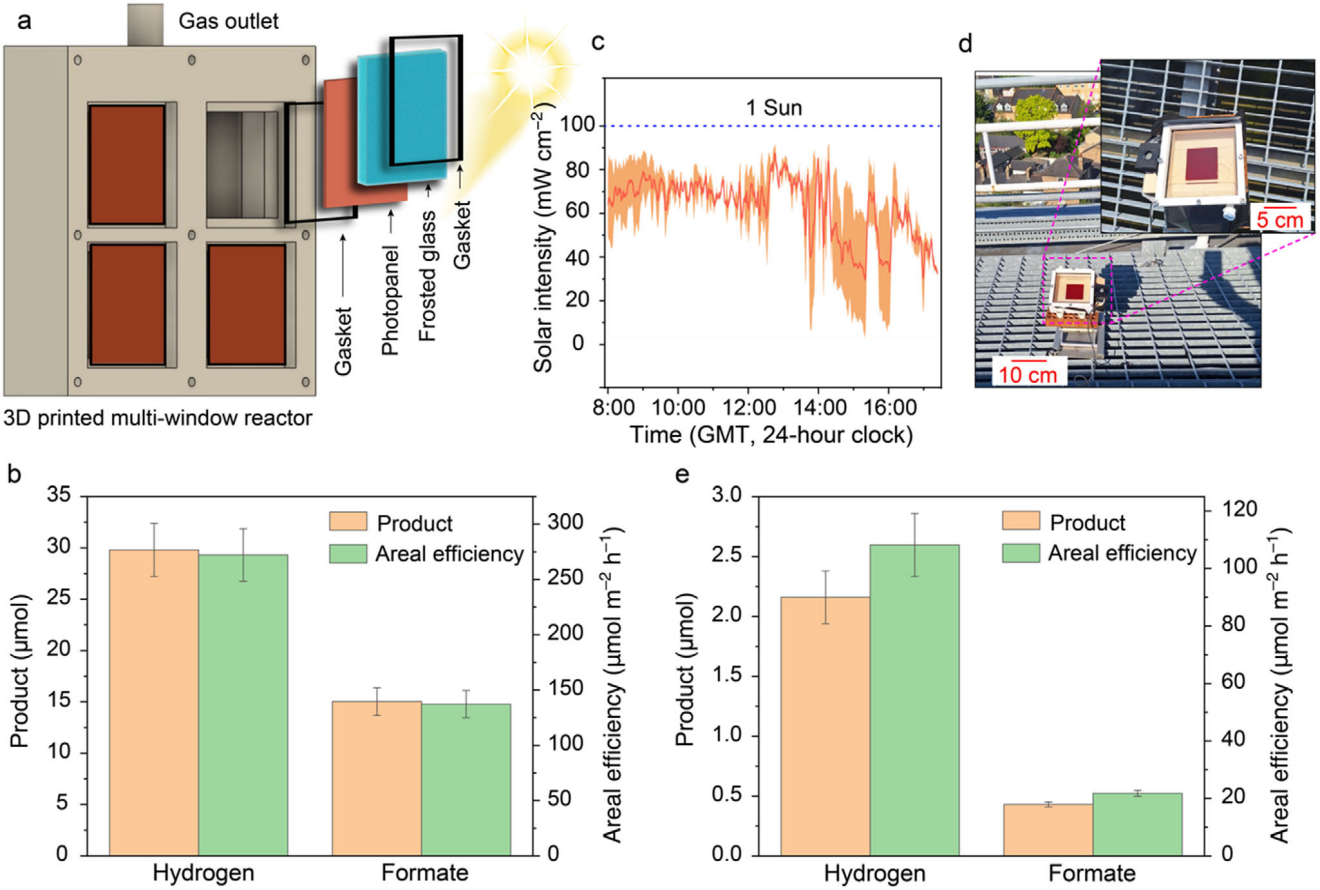
Demonstration of solar reforming: (a,b) multi‐window reactor operated indoors at pH 7 and (c–e) single‐window reactor operated outdoors at pH 14. a) Design of a multi‐window reactor and its components. b) Bar plots showing the H_2_ and HCOO^−^ generation and efficiency during solar reforming using a multi‐window reactor having active surface area of 54.76 cm^2^ (see Figure S50, Supporting Information); conditions: COF|ITO|CN_x_|NiME photopanel, 10% v/v EG, and back irradiation (AM 1.5 G, 100 mW cm^−2^). c) Variation of real sunlight intensity during direct plastic solar reforming. d) Digital image of the reactor during plastic solar reforming under direct sunlight; inset: zoomed image of the reactor. e) Efficiency of a 25 cm^2^ COF|ITO|CN_x_|Pt photopanel during plastic reforming under direct sunlight; conditions: pretreated PET plastic in 1 M KOH, irradiation under direct sunlight as shown in Figure 5c.

To demonstrate modular scalability, the photopanel was integrated into a 3D‐printed multi‐window reactor (**Figure** **5**a; Figures S49 and S50, Supporting Information), utilizing a total active area of ≈55 cm^2^. An areal activity of 272.1 ± 23.8 µmol H_2_ m^−2^ h^−1^ and 137.2 ± 12.4 µmol HCOO^−^ m^−2^ h^−1^ was achieved (Figure 5b; Table S17, Supporting Information). The high areal efficiency under back‐illumination highlights the potential for practical application, where light penetration through turbid media critically impacts semiconductor performance (Discussion S11, Supporting Information).

### 2.7 Plastic Solar Reforming Under Direct Outdoor Sunlight

Finally, the 5 × 5 cm^2^ glass‐supported photopanel was deployed under direct sunlight for plastic upcycling, using a pretreated PET plastic waste stream (Figure  5c,d). A commercial PET bottle was first cut into small pieces, shredded, and ground into microplastics, followed by alkaline pretreatment (50 mg plastic per mL KOH) in 1 m KOH at 80 °C for 7 days to yield EG and terephthalate as major depolymerized products (Figures S51 and S52, Supporting Information). Considering the limited stability of Ni‐based catalysts and the superior stability of Pt under harsh alkaline conditions (pH ≥ 10)^[^ ^3e^ ^]^ (Figure S53, Supporting Information), the COF|ITO|CN_x_|Pt photopanel was employed for this process (Discussion S12, Supporting Information). Certain degradation of the panel was also observed under prolonged exposure to the highly basic medium that resulted in formation of different oligomers along with the building units *4,4´*‐azo dianiline and *2,4,6*‐triformylphloroglucinol (Figures S54 and S55, Supporting Information). However, control experiments confirmed that the building blocks (diamine and trialdehyde) did not contribute to solar reforming under these conditions (Table S18 and Discussion S13, Supporting Information). Despite the relatively low sunlight intensity during the outdoor experiments in the UK (25^th^ and 26^th^ June, 2024, Cambridge, UK) (Figure 5c,d), the photopanel generated 2.1 ± 0.2 µmol of H_2_ and 0.4 ± 0.1 µmol of HCOO^−^ within 8 h of direct solar exposure (Figure 5e; Figure S56 and Table S19, Supporting Information). Notably, an areal activity of 108.2 ± 10 µmol H_2_ m^−2^ h^−1^ was achieved for plastic valorization under natural sunlight.

The overall solar driven H_2_ activity of the COF|ITO|CN_x_|NiME composite remains modest compared to some state‐of‐the‐art photopanels for hydrogen production using noble‐metal‐based photocatalysts designed for overall water splitting.^[^ ^23^ ^]^ However, our system demonstrates a meaningful advancement within the domain of organic semiconductors employing noble‐metal‐free cocatalysts toward solar reforming generating valuable chemical feedstocks like HCOO^−^ along with H_2_ (Table S20, Supporting Information).^[^ ^24^ ^]^ To date, there is very limited progress reported on direct plastic photoreforming with concurrent hydrogen generation using scalable organic semiconductor‐based photopanel architectures.^[^ ^5^ ^]^ Against this backdrop, our composite achieves a notable improvement in areal hydrogen production (108.2 ± 10 µmol H_2_ m^−2^ h^−1^) compared to prior CN_x_‐based systems (52 µmol H_2_ m^−2^ h^−1^) and offers high selectivity toward oxidative formate production. Beyond activity, the COF|ITO|CN_x_|NiME platform offers additional advantages including facile integration into glass‐supported panels or standalone photoleaves, and extensive synthetic tunability through modular tailoring of the COF pore interior. While there remains scope for improving the overall activity with improved cocatalyst(s), the demonstrated scalability, product selectivity, and structural adaptability establish this system as a promising platform for decentralized plastic valorization under direct sunlight.

## 3 Conclusion

We have introduced a modular COF|ITO|CN_x_ platform capable of scalable solar‐driven plastic upcycling, operating both under simulated and natural sunlight. By engineering macroscopic architectures, from standalone leaves to glass‐supported photopanels, we enable robust and scalable photoreforming with high areal efficiencies, even under low solar flux. The porous photocatalyst system with intrinsic nanoconfinement promotes selective product generation, addressing a central challenge in heterogeneous solar catalysis. The strategy of constructing thin, porous, and processable photocatalytic films addresses a key limitation in scaling solar chemical technologies, particularly the constraints imposed by light penetration, mass transport, and mechanical robustness. Through controlled photopanel fabrication and reactor modularity, we provide a generalizable blueprint for designing light‐driven chemical panels that can be readily adapted to different substrates, waste streams, and catalytic chemistries. The use of other polymeric organic photocatalysts with different functionalization offers flexibility in tuning light absorption, charge transport and catalytic performance. This will allow the extension of this modular photoreforming platform to CO_2_ valorization and exploring other chemical reactions in the future.

## 4 Experimental Section

### Synthesis of COF^[^ ^16^ ^]^

In a mixture of 2.5 mmol (475.5 mg) of *p*‐toluene sulphonic acid and 0.45 mmol (95.5 mg) *4,4´*‐azo dianiline, 0.3 mmol (63 mg) *2,4,6*‐triformylphloroglucinol was added followed by grinding and addition of measured amount of water (100 µL). After mixing them uniformly, they were transferred to a sealed vial and left for 12 h at 90 °C. The solid was sequentially washed with water, DMAc and acetone; then dried under vacuum to result in a deep reddish powder with 74.2 ± 0.8 yield.

### Synthesis of CN_x_^[^ ^15^ ^]^

Melamine (5 g) was heated at 550 °C for three hours (ramping rate = 1 °C min^−1^) under air. The resulted yellow CN powder (50% yield) was ground to fine powder using mortar and a pestle. The as‐synthesized CN powder was then mixed with potassium thiocyanate (weight ratio 1:2) and then heated sequentially at 400 °C for an hour followed by at 500 °C for 30 min (ramping rate 30 °C min^−1^) under Ar. Then the powder was washed thoroughly with distilled water and dried initially at room temperature then under vacuum at 80 °C. 1 g of the CN_x_ was taken in a stainless‐steel ball milling container. 3 mm 25 steel balls were placed inside the container as well. Then ball milling was performed for 20 min followed by 5 min halt and then for another 20 min at 25 Hz resulting in CN_x_ nanoflakes. The sample was collected from the container and then dried again under vacuum before further use.

### Synthesis of COF|CN_x_

In a mixture of 2.5 mmol (475.5 mg) of *p*‐toluene sulphonic acid and 0.45 mmol (95.5 mg) *4,4´*‐azo dianiline, 0.3 mmol (63 mg) *2,4,6*‐triformylphloroglucinol was added followed by grinding and addition of measured amount of water (30 µL). After uniform mixing, 147.3 mg of CN_x_ nanoflakes were added and mixing was continued. Then, additional 70 µL water was added in 3–4 batches. After every batch of water addition, the mixture was thoroughly ground to paste. The viscous paste was then transferred to a vial and left for freeze drying for 12 h. The solid was then placed inside of a preheated oven (at 90 °C) for 20 h. The resulted composite was then sequentially washed with water, DMAc and acetone and dried under vacuum to result in COF|CN_x_ composite.

### Synthesis of COF|ITO|CN_x_ Powder

1.3 mmol (250 mg) of *p*‐toluene sulphonic acid and 0.23 mmol (48 mg) *4,4´*‐azo dianiline was ground thoroughly and then 0.15 mmol (31.5 mg) *2,4,6*‐triformylphloroglucinol was added to the mixture and mixed properly. 30 µL of water was added to the mixture and then 72.4 mg (1 equivalent with respect to COF) of CN_x_ was added and grinding was continued with occasional addition of water (30 µL) until a uniform paste is formed. Then 36.2 mg ITO nanoparticle was added and the mixing was continued with addition of water (60 µL). Then the viscous paste was transferred and freeze dried overnight. The solid powder was transferred to sealed vial and left for 20 h at 90 °C. The solid was sequentially washed with water, DMAc and acetone and dried under vacuum to result in COF|ITO|CN_x_ composite with mesoporous surface (more information in Discussion S2 and S3, Supporting Information). In case of unmodified surface structure, the only step of freeze drying was omitted. For various compositions of COF|ITO|CN_x_ contents, the same process has been followed, only varying the amount of the building units.

### Synthesis of COF|ITO|CN_x_ Standalone Photoleaf and Photopanel

A standalone photoleaf from COF|ITO|CN_x_ was fabricated following the similar protocol as powder (described above). Initially, a homogeneous paste was prepared by mixing *p*‐toluenesulfonic acid (0.65 mmol, 125 mg), *4,4′*‐azodianiline (0.12 mmol, 24 mg), *2,4,6*‐triformylphloroglucinol (0.8 mmol, 16 mg), CN_x_ (36.2 mg, corresponding to the COF formation ratio; see Discussion S2, Supporting Information) sequentially with the addition of water (100 µL). Then, ITO nanoparticles (18.1 mg, 0.25 equivalent relative to COF formation) were incorporated, followed by further grinding with incremental water additions (25 µL × 4) to achieve a viscous and uniform paste. Then, the paste was casted on a glass slide with photoleaf template. The template was produced using kapton tape. The tape was removed as soon as casting was completed. Then the glass slide was placed inside a vacuum glass desiccator with 100 µL water inside. The desiccator was then placed inside a preheated oven at 60 °C for 4 days. The resulted photoleaf was cooled to room temperature and washed with water, DMAc and acetone and dried under vacuum at 60 °C. One of the key steps is to maintain the right viscous nature of the paste. The atmospheric humidity has an impact on this as the paste may lose moisture thereby deviating from the right proportion of viscosity. Thus, amounts of water may vary. A COF|ITO|CN_x_ photopanel was fabricated following the similar protocol as standalone photoleaf. After the addition of ITO, the paste was casted on a frosted glass with a doctor blade. Then the glass slide was treated with liquid nitrogen followed by freeze drying for 12 h. The glass plate was placed inside a preheated oven at 60 °C for 3 days. The resulted photopanel was sequentially washed with water, DMAc, acetone and water before further use.

### Synthesis of COF|ITO|CN_x_|NiME Composite

NiME has been synthesized following literature protocol.^[^ ^10^ ^]^ For a standard measurement, 2 mg of CN_x_|ITO|COF composite has been placed in 1 mL 1.25 µm NiME solution prepared in water. Then the dispersed solution was ultrasonicated for 20 min followed by stirring for 2 h at 600 rpm. Then, the precipitate was collected by centrifuge and washed with water for 3 times before overnight drying in vacuum at 50 °C. For the loading of NiME, the photopanel and photoleaf were submerged in the NiME solution (1 mL 0.63 µM NiME solution for 1 mg composite) for 12 h and then washed multiple times with water before further usage.

### Photocatalytic Generation of H_2_

In a 7.91 mL (internal volume) pyrex glass photoreactor vial, 2 mg of COF|ITO|CN_x_|NiME was taken in 1 mL water. Then 100–500 mm substrate (EG, glycerol and sugars) was added and the vial was capped with a rubber septum. First, the sample was vortexed followed by bath sonication. Then, N_2_ (containing 2% CH_4_) was purged at ambient pressure for 20 mins. The sample was placed under simulated sunlight (solar light simulator, Newport Oriel), calibrated to 1 Sun (100 mW cm^−2^) with a Newport optical power meter. The solar simulator is equipped with an air mass 1.5 global filter (AM 1.5 G) and a water filter for removing infrared radiation. During the experiment, the sample was stirred at 600 rpm and temperature at 25 °C throughout the reaction timeline. Solar reformed H_2_ was quantified by analyzing the reactor headspace gas (50 µL) using a gastight syringe (Hamilton) by a Shimadzu GC‐2010 Plus gas chromatography (GC) with a barrier discharge ionization detector (BID). The generated HCOO^−^ during solar reforming was analysed by ion chromatography (IC) using a Metrohm 882 Compact IC plus system with a solution of 3.2 mm sodium carbonate and 1 mm sodium bicarbonate as the eluent. ^1^H NMR spectroscopy was performed on a Bruker 400 MHz NMR spectrometer using D_2_O. Tetramethylsilane (TMS) and maleic acid have been used as internal standard.

### Photocatalytic Generation of H_2_ with a Photoleaf and Photopanel

COF|ITO|CN_x_|NiME photoleaf was placed inside a top‐down photoreactor (with an optical window at the top and total volume of 50 mL; Figure S41, Supporting Information) containing 5 mL water and 500 mm EG. For the multi‐window reactor, the total active area is 54.76 cm^2^ with a total internal volume of 218 mL. A specially designed reactor has been printed that possesses diameter and length = 12.5 cm, height = 3 cm, reservoir length and width = 10.5 cm, height = 2.17 cm, each solar window = 3.7 cm, frosted glass length and width = 4.5 cm, thickness = 3 mm, gasket thickness = 2.8 mm. The reactors were purged with N_2_ (containing 2% CH_4_) at ambient pressure for 30 min and then placed under solar simulator (LOT‐Quantum Design, 100 mW cm^−2^) with AM 1.5G global filter at room temperature. H_2_ content was measured from the reactor head space gas and HCOO^−^ was quantified from the solution. Current design allows the illumination from top in case of top‐down photo reactor and single window reactor. It was also possible to maintain the temperature at 25 °C during experiment. The multi‐window reactor was placed vertically and illumination was from side. The temperature was not controlled in case of back illumination.

### Solar Reforming with Simultaneous Plastic Valorization Under Real Sunlight

Solar reforming was studied outdoors with real‐world PET plastic. First, plastic bottles were shredded to very small pieces. Then, 25 g of shredded plastics were taken in 500 mL of 1 m KOH. The mixture was stirred at 80 °C for 7 days. The solution was cooled to room temperature and used directly thereafter. The formation of EG and terephthalate was confirmed by ^1^H NMR spectroscopy. Measured amount (49.58 and 49.54 mL) of the PET plastic treated KOH solution was taken in 280 mL single window reactor (over all reactor length and width = 12.5 cm, height = 3.5 cm, reservoir length and width = 11.2 cm, reservoir height = 2.57 cm and solar window = 10.5 cm: Figure S41, Supporting Information). From a 1000 ppm Pt nanoparticle solution, measured amount (420 and 460 µL for two batches) was added to make the Pt content 2 wt.% of the photocatalyst (21 and 23 mg). The total volume of the solution in the reactor was made to 50 mL. Then the reactor was placed on the roof top tilting toward the sunlight. The reactor was rotated manually to keep aligned with the sunlight direction. The intensity of sunlight was continuously measured with an optical power meter placed next to the reactor. The temperature of the reactor was measured at different time interval. Finally, H_2_ was quantified from the headspace and reaction solution was used for quantifying HCOO^−^.

### External Quantum Efficiency (EQE) Measurements

EQE was determined using a LOT MSH300 monochromator and a LOT LSH302 light source with a 300 W Xe lamp. Experiments were performed over 24 h, using monochromatic light at a wavelength of 400 nm, with a full width at half‐maximum (FWHM) of 15 nm. The light intensity of ≈6.3 mW cm^−2^ was determined using a Thorlabs PM100D power meter with a Thorlabs S302C thermal power sensor head. photocatalyst suspensions (4 mg mL^−1^) were stirred in a quartz vial, which was irradiated through a 1 cm^2^ mask. The evolved H_2_ amounts were determined via a Shimadzu GC‐2010 Plus instrument. Accordingly, the EQE values were determined using the formula below ($n_{H_{2}}$ – amount of H_2_, N_A_ – Avogadro's constant, h – Planck's constant, c – speed of light, t – duration of experiment, λ – wavelength, I – light intensity, A – irradiated area).

(1) $$EQE=\frac{2n_{H_{2}}N_{A}hc}{t\lambda IA}\times 100\%$$

### Photoelectrochemistry

Linear sweep voltammetry (LSV), chronoamperometry (CA) and PEIS experiments were carried out in an electrochemical cell with a 3‐electrode configuration: a COF|ITO|CN_x_ working electrode, a Pt mesh counter electrode, and an Ag/AgCl (saturated NaCl) reference electrode. The 12 mL electrolyte contains 0.5 m Na_2_SO_4_ (pH 7), and 100 mm EG. The COF|ITO|CN_x_ composite was deposited on an FTO glass. 14 mg of composite was taken in 300 µL of ethanol and sonicated for an hour to prepare the ink. Then a total of 20 µL (10 µL × 2) of ink was drop cast within an area of 0.25 cm^2^ on FTO glass. The sample was dried at room temperature for 30 min followed by annealing for 3 h at 150 °C under argon atmosphere. The COF|ITO|CN_x_ coated FTO was dipped in 1 mL 0.63 µm solution of NiME for 12 h followed by washing in water to construct the COF|ITO|CN_x_|NiME on FTO. A 500 W xenon arc lamp (Newport 67005) was calibrated to AM 1.5G and used as light source. Impedance response was recorded with a potentiostat (IviumStat) with frequency ranges from 2 kHz to 0.1 Hz and a 25 mV sinusoidal amplitude. Impedance data was fitted with equivalent circuits using modeling software ZView2 (Scribner Associates).

## Conflict of Interest

The authors declare no conflict of interest.

## Supporting information



Supporting Information

## Data Availability

The raw data supporting the findings of this study are available from the University of Cambridge data repository: https://doi.org/10.17863/CAM.121168.
