# Supplementary material for: Covalent Organic Framework and Carbon Nitride Composite for Scalable Solar Reforming
Source: Adv Mater. 2025 Sep 26;38(1):e13457. doi: 10.1002/adma.202513457 (PMC12759194; doi:10.1002/adma.202513457)
Supplement: Supplementary file 1 — Supporting Information [file ADMA-38-e13457-s001.pdf]

ADVANCED MATERIALS

Supporting Information

for *Adv. Mater.*, DOI 10.1002/adma.202513457

Covalent Organic Framework and Carbon Nitride Composite for Scalable Solar Reforming

*Suvendu Karak, Yongpeng Liu, Ariffin Bin Mohamad Annuar and Erwin Reisner**

Supporting Information

Covalent Organic Framework and Carbon Nitride Composite for Scalable Solar Reforming

Suvendu Karak, Yongpeng Liu, Ariffin Bin Mohamad Annuar, and Erwin Reisner*

Yusuf Hamied Department of Chemistry, University of Cambridge, Lensfield Road, Cambridge CB2 1EW, United Kingdom

E-mail: reisner@ch.cam.ac.uk

Table of Contents

Discussions S1–S13	Page S3 to S12
Tables S1–S20	Page S13 to S23
Figure S1–S57	Page S24 to S57
References	Page S58 to S59

Discussion S1. Selection of azo-functionalized COF.

The chosen Azo-COF exhibits high crystallinity and possesses ordered intrinsic mesopores with a diameter of approximately 2.7 nm. This high degree of crystallinity reflects long-range atomic precision within the framework, enabling better understanding and precise integration of components within the composite. In contrast, poorly crystalline COFs would likely form amorphous composites upon integration with ITO and CN_x, limiting structural insight and potentially compromising photocatalytic performance.

The large surface area and ordered mesoporous channels of the Azo-COF facilitate accelerated photocatalytic reactions by providing abundant substrate docking sites, accessible active sites, and improved mass transfer. The nanoconfinement effect, well-documented in porous materials, enhances selective substrate conversion within the confined pores. Specifically, the intrinsic mesopores of the Azo-COF enable effective adsorption of ethylene glycol (EG) and selective oxidation to formate during solar reforming.

The β-ketoenamine linkages in the COF backbone are renowned for their exceptional chemical stability, proven under acidic, and basic conditions.[1] This stability is critical for long-term photocatalytic durability. Such linkages have been rigorously tested and shown to perform well in diverse photocatalytic reactions, including hydrogen generation, water oxidation, and pollutant degradation.[2] Recently, β-ketoenamine-linked azo-based COFs have been utilized as porous pigments for organic transformations.[3] Notably, azo-functional COFs demonstrated significantly higher photocatalytic activity compared to other photoactive cores such as *6*-phenylphenanthridine, *2,2'*-bipyridine, and *1,3,5*-tris(phenyl)benzene in metal-free C–B bond formation reactions.

From an optical perspective, the broad absorbance range of the Azo-COF (350–700 nm) and its relatively low band gap (1.95 eV) facilitate facile electron excitation, which is essential for efficient charge relay in the Z-scheme photocatalytic mechanism. Correspondingly, the Azo-linked COF suppresses the recombination of excited-state electron–hole pairs and perform better compared to its imine-based counterpart.[4] Moreover, the unique band positions of the Azo-COF enable the construction of the Z-scheme, unlike many typical COFs where the conduction band lies significantly above the proton reduction potential. Additionally, thin films of residual crystallized Azo-COF exhibited higher electrical conductivity (3.7×10^{-2} mS cm^{-1}) compared to other COFs, further supporting effective charge transport.[5]

Discussion S2. Composition of COF|ITO|CN_x.

During the synthesis, the resulted yield of COF formation is 74.2±0.8 %. Then, COF was synthesized in presence of measured amount of CN_x where the yield was found to be 92.9±0.2 within the COF|CN_x composite. Then yield of COF was measured to be 91.9±0.8 % within COF|ITO|CN_x composite (Table S1, Supporting Information). As both CN_x and ITO are insoluble in the reaction medium even during purification method, the additional increase in mass is only due to the higher yield of COF. Based on this knowledge, we have considered the

amount of CN_x to be added during synthesis of composite so that the COF and CN_x ratio becomes 1:1 and ratio of COF to CN_x to ITO becomes 1:1:0.5. The amount of ITO nanoparticles has considerable impact on the processability. Due to their highly crystalline nature, ITO nanoparticles make the processed objects/nanostructures fragile in nature. Thus, as-synthesized composite peeled off from the glass surface after drying when there is high ITO (0.5 ratio) content in the composite. In order to avoid this, we have decreased the ITO amount so that COF:CN_x:ITO becomes 1:1:0.25 in the final composite for standalone photoleaf and photopanel. The detailed synthetic process and amounts of individual reactants have been discussed in the 'Methods' section of the manuscript. Similarly, in order to construct COF|ITO|CN_x composites with various ITO, CN_x and COF content, amount of ITO and CN_x has been varied.

Discussion S3. Effect of hierarchical pores.

The hierarchical porous structures are well-known for rapid pollutant removal and catalysis. Generally, most of this pore remains inter-connected.[6] As a result, substrates can easily diffuse through the material and reach to the catalytic centers. However, there is a need of intricate balancing between porosity and crystallinity. During the solid-state synthesis of the composite measured amount of water is added. The added water is extracted rapidly from the structure during freeze-drying process and inverse opal-like structure is created along with hierarchical porosity. It was found that the increase in water content changes the overall morphology as well as crystallinity (long-range order) of the composite. Another important aspect is the time duration. We have found that freeze-drying for 3 days significantly decreases the crystallinity of the material leading to more amorphous content within the composite. It is well known from the mechanism of COF formation that the reversibility during imine bond formation is the key factor for such structure (Figure S3) and water plays a key role there.[7] Longer time in freeze-drying does not leave any water in the reaction medium resulting poor crystallinity of the composite.

Discussion S4. Stacking modes of COF and CN_x in the composite.

For various applications, COFs have been made into composites. Varying their synthetic methodology, they have been turned into foams, aerogels and other 3D macrostructures.[8] In the salt-mediated crystallization approach, composites with different COFs[9] and graphene[10] have been realized. During their structural investigation, it has been observed that the COF crystallites grow on the surface of other support like graphene. Even graphene nano-sheets intercalated between the layers of the COF, resulting the broadening/shifting of the PXRD peak from (001) plane. Thus, in such composites, all the components are present quite uniformly throughout the material rather than aggregating in certain areas. Generally, phase segregation often limits in actuating the full potential in each component. Evenly distribution of all components helps in improving catalysis and other capturing process, as microenvironments

are thoroughly maintained. Before the synthesis of the composite, CN_x was ball-milled for 30 mins to turn them into smaller nano-flakes (details in method section). It helps them in intercalating between the COF layers.

Now from Bragg's law,

$$n\lambda = 2d \sin\theta$$

where,

n is the order of diffraction
λ is the wavelength of the X-ray radiation
d is the interplanar spacing (distance between layers)
θ is the angle of incidence of the X-ray beam

Any change in the θ value corresponds to the interlayer spacing. In specific, the decrease in the theta value refers to the larger interlayer spacing and vice versa. The high intensity diffraction pattern from the ITO nanoparticle hinders the close inspection of the diffraction pattern of the COF|ITO|CN_x composite. Thus, to get more insights of the order of the composite, we have analyzed the PXRD pattern of the COF|CN_x composite (Figure S4). The analysis clearly shows that the peak arises due to the interlayer spacing stretches between 20.2-28.2 degree. The intensity maxima for CN_x appears at $2\theta = 28$ degree whereas for pure COF the peak from (001) plane is at $2\theta = 26.6$ degree. From, Bragg's equation, the inter layer spacing between two COF layers is determined as 3.4 Å (Fig. 2a, blue trace and Figure S5a,b). However, from the PXRD pattern of the COF|CN_x composite, it is observed that the interlayer spacing increases significantly (where d = 4.3 Å) as 2θ value decreases. This is due to the intercalation of the CN_x layer between the COF layers (Figure S5bd). It is quite unlikely to have such intercalation due to π– π stacking between CN_x and COF layers as the distance between two layers is ~ 2.2 Å (Figure S5c). Instead, such structures will be stabilized *via* van der Waals force and hydrogen bonding.[10] It is also possible that CN_x layers have been sandwiched between multiple COF layers (Figure S5e). Based on their layer sizes, interaction between CN_x layers and COF layers varies. As a result, the distance between a CN_x layer and COF layer changes, thereby broadening the peak.

Discussion S5. T-Plot report calculation.

T-plot analysis provides crucial additional information from N_2 adsorption isotherm. It provides key insights into the pore structure of materials, particularly differentiating between microporous and macroporous regions. Although t-plot report doesn't provide direct information of pore size distributions, it quantifies the amount of micropores and macropores. Herein, thickness has been measured following Harkins-Jura equation:

$$\mathbf{t = [13.99/(0.034 - \log(P/P_o))]^{\wedge}0.5}$$

where,

t: Statistical thickness of the adsorbed layer (in angstroms)
13.99 and 0.034: constants specific to nitrogen adsorption at 77 K
P: Pressure of the adsorbate gas

P_o: Saturation pressure of the adsorbate gas at experimental temperature

From the slope and Y-intercept of thickness Vs. Quantity adsorbed curve, we get the external and microporous surface area, respectively. The slope at low t-values (thicknesses) corresponds to microporous region (pore smaller than 2 nm) whereas slope at higher t-values represents mesopores and macropores. Here, thickness has been measured in Å and adsorbed N_2 amount in $cm^3\ g^{-1}$. If the Y-intercept is A $cm^3\ g^{-1}$ STP and slope is B $cm^3\ g^{-1}\cdot Å^{-1}$ STP, surface areas have been calculated as,

Micropore Surface Area = A $cm^3\ g^{-1}$ STP * 0.0015511 g cm^{-3} * 6.022 × 10^{23} molecules/mol / (16.2 $Å^2$) * 10^{-20} $m^2Å^{-2}$

External Surface Area = B $cm^3\ g^{-1}\cdot Å^{-1}$ STP * 0.0015511 g cm^{-3} * 6.022 × 10^{23} molecules/mol / (16.2 $Å^2$) * 10^{-20} $m^2Å^{-2}$

For N_2 gas, the density conversion factor is 0.0015511 g/cm^3 that converts the adsorbed amount from grams to cubic centimeters. 6.022 × 10^{23} molecules/mol is the Avogadro's number and 16.2 $Å^2$ is the cross-sectional area of a single nitrogen molecule. 10^{-20} $m^2/Å^2$ is the conversion factor for converting square angstroms ($Å^2$) to square meters (m^2). For the appropriate measurement, the T-plot analysis was performed within a thickness range of 3-5 Å.

Discussion S6. Selection of substrates for oxidation reaction.

Solar reforming provides an important avenue for simultaneous mitigation of pollutants both through oxidation and reduction reactions. Here we have considered of generating H_2 as green fuel at reduction site using molecular cocatalyst. In both cases, EG has been oxidized to generate value-added product like formate. One of the main reasons of selecting EG as reductant during solar reforming, is that it is the byproduct of PET plastic pretreatment along with terephthalate (base-treatment). This allows direct waste mitigation and valorization of plastic in a sustainable pathway during oxidation reaction. EG has been generated by treating PET plastic with 1 (M) KOH at 80 ºC for 7 days (50 mg plastic per mL KOH). However, other than the realistic demonstration, pure low concentration of EG has been used. Pure EG can be collected from the pretreated PET plastic medium by distillation or organic solvent extraction process.[11]

The solar reforming efficiency (for H_2 generation) is still not the highest, as some of the reported panel systems uses highly active Pt nanoparticles along with triethanol amine and ethanol oxidation (Table S19, Supporting Information). The usage of such highly water soluble and easily oxidisable sacrificial electron donors would lead to the higher efficiency of the system. However, the waste reforming provides more sustainable opportunity in the solar reforming system where sugars can be extracted from food wastes and EG is the product of plastic pretreatment.

Discussion S7. Addition of cocatalyst during durability test.

The NiME cocatalyst has shown considerable stability over 24 h, as mentioned in the literature. The UV-vis spectrum showcases that the water solution of the bare complex is not stable under direct light for 72 h (Figure S22). It is well known in literature that metal complexes and their highly reactive species could be confined inside the COF nanopores and catalysis are performed within the pores.[12] It is even possible to stabilize the water unstable reactive species inside the nanopore and perform catalysis.[13] So, it is expected that the stability of this hexameric structure would be enhanced upon encapsulation inside the nanopores of the COF|ITO|CN_x composite. That is why although the NiME is not stable for 3 days in solution phase, we still find photocatalytic activity in heterogeneous phase. The presence of the complex inside the pore after 3 days of catalysis has been confirmed from the mass spectrum (Figure S23). However, we have found out from the activity curve of hydrogen evolution that the activity starts decaying just after 72 h. This would be due to the degradation of some portions of cocatalyst only as photocatalyst is highly stable under this reaction condition as confirmed by recyclability test (Figure S24 and S25, Supporting Information). The reaction was continued for another 2 days before we added cocatalyst externally to the reaction medium (132nd h of reaction). The cocatalyst was solubilized in water and then purged with 2% methane in N_2 gas for 15 mins. Then, 50 µL of the 1.25 µM NiME solution was injected in the reaction medium. The increase in total solution volume was considered for future calculation of the head space gas. H_2 evolution did not improve immediately (135th h). However, there is a sharp increase in performance after 6 h of addition. This is due to the time delay for the cocatalyst immobilization on the photocatalyst.

Discussion S8. Hydrogen evolution kinetics.

The reaction system requires time to reach optimal activation, where the interplay between carbon nitride, COF, and ITO is fully established. The dynamic interaction between porous COF|ITO|CN_x|NiME composite enhances over time, leading to higher activity. Early-stage limitations in mass transport and charge carrier movement diminish as the reaction proceeds. As light irradiation continues, the generation, separation, and transfer of photogenerated carriers (electrons and holes) improve. It has also been found that the rate of H_2 evolution increases 1.5 times (0.358 µmol h^{-1} vs 0.229 µmol h^{-1}) after 15 h of reaction (Figure S57). This could be due to the diffusion limitations of the substrates at the beginning of the reaction.

- The COF’s porous structure facilitates efficient diffusion of ethylene glycol to the active sites. At the start, reactant diffusion may be slow, limiting the reaction rate.
- As intermediates and products accumulate within the pores, a self-enhancing effect may occur. The presence of intermediates could activate more catalytic sites or improve reactant accessibility. The porosity enables continuous transport of ethylene glycol, reducing concentration polarization effects.
- As ITO serves as an efficient electron mediator, transferring electrons from COF to carbon nitride, there may be a lag in charge carrier transfer at the early phase of the reaction due to initial recombination of photogenerated carriers and slow establishment

of a steady-state electron flow. Over time, the charge transfer pathway becomes more efficient as the system equilibrates, enhancing the rate of hydrogen evolution.

- Ethylene glycol oxidation on the COF can also play a crucial role as the oxidation products may alter the local reaction environment (e.g., pH or surface energy) to favor hydrogen evolution. As ethylene glycol oxidation proceeds, the reaction dynamics may self-accelerate due to the improved hole scavenging efficiency, which indirectly increases the rate of hydrogen evolution.
- Under continuous light irradiation, photon flux absorption by the photocatalysts may increase as dynamic rearrangements of the catalyst surface (e.g., desorption of byproducts) could expose more active sites.

Over time, the system reaches a state of enhanced activity, contributing to an exponential increase in the rate.

Let's consider that R(t) is the amount of hydrogen produced per unit time per amount (gm). Consider no mass change was observed during the catalysis and the rate exponentially increases with time. Then,

$$R(t) = R_0 e^{kt}$$

where:
R_0: Initial rate of hydrogen evolution at t = 0 (unit: μmol/s).
k: Growth constant (unit: s^{-1}), determining the rate of exponential increase. A small k indicates slow exponential growth.
t: Time (unit: second)

If H(t) is the cumulative hydrogen production i.e., the total amount of hydrogen produced up to time t, then,

$$H(t) = \int_o^t R(t)\, dt$$

$$H(t) = \int_o^t R_0 e^{kt}\, dt = \frac{R_0}{k}\, e^{kt} + C$$

where:
C is the constant of integration. If H(0) = 0 (i.e., no hydrogen is produced at t=0), then

$$C = -R_0/k \text{ and}$$

$$H(t) = \frac{R_0}{k}\, (e^{kt} - 1)$$

For slow exponential growth ($k \ll 1$), the exponential term e^{kt} can be approximated using a Taylor expansion:

$$e^{kt} \approx 1 + kt\ (for\ small\ k\ and\ t)$$

Substituting this approximate into H(t):

$$H(t) \approx \frac{R_0}{k}\, \big((1 + kt) - 1\big) = R_0 t$$

This shows that the cumulative hydrogen increases **linearly** with time for small k, where $H(t) \propto t$.

Discussion S9. Nano confinement and catalysis.

The hierarchically porous structures offer confined nanoscale environments that has significant influence on the catalysis process specially impacting the behavior of reactants and intermediates.[14] The rigid and well-defined pore architecture of COF enables selective diffusion of the reactants inside the pores and stabilizes intermediates, thereby enhancing the selective product formation.[13] There are several factors involved in the nanoconfinement:

a) It is due to the spatial confinement; the mobility of the reactants and products are restricted. As a result, multi-step catalysis could undergo within the pores preventing undesired side reactions and providing preferred orientations of the reactant sites.
b) The close proximity of the reactants to the catalytic sites impacts the electronic property of the catalyst. For an example, molecular docking of substrate on the photocatalyst surface can have impact on light absorption, electron transfer process etc.
c) The hierarchically porous structures have significant role on the catalysis that has already been mentioned in Discussion S3 (Supporting Information). The interconnected micro-, meso- and macro-pores would help in faster diffusion of the substrates to the reaction site. Similarly, the product can be diffused to the reaction medium thereby enhancing the rate of catalysis. Thus, mass transfer process will be significantly impacted during the catalysis inside the nano-pores.
d) The adsorption and desorption of substrate could be dynamic through the nanopores of COF|ITO|CN_x|NiME composite. The adsorption capacity of the different composites has been measured and this justifies the conversion of EG inside the pores (Figure S19 and S20, Supporting Information).

Here during solar reforming of EG, there are possibilities of various product formation such as glycolaldehyde, glycolaldehyde dimer, glycolic acid, formate, acetate etc (Figure S26). However, in the case of hierarchically porous COF|ITO|CN_x|NiME composite, the oxidation of EG occurs within the pores facilitating the selective conversion to formate. Similar observation has been found for other sugars as well. During this conversion, the composite was filtered and washed with organic solvent (dichloromethane and acetonitrile) and characterized with Liquid chromatography–mass spectrometry (LC–MS) (Figure S28). We were able to find various intermediates that leads to the formation of formate. This suggests that step-wise conversion (6 e^- transfer process occurs) occurs within the nanopores.

Discussion S10. Mechanism of Z-scheme solar reforming.

Based on the absolute band edge positions, the COF (VBM =−7.64 eV, CBM = −5.64 eV), CN_x (VBM = −7.01 eV, CBM = −4.37 eV), and ITO (Fermi level = −4.7 eV) construct a Z-scheme heterojunction. Upon visible-light illumination, both COF and CN_x generate electron–hole pairs. The photogenerated electrons in the CB of COF (−5.64 eV) recombine with holes in the VB of CN_x (−7.01 eV) through ITO, which is a degenerate semiconductor and possesses metal-like electrical conductivity. Thus, ITO is well suited to act as an efficient electron mediator.

The presence of ITO significantly accelerates charge transfer and reduces interfacial resistance, resulting in more efficient carrier migration across the heterojunction.[15] ITO is also known to possess defect-rich sub-bandgap states, which serve as trapping and transfer sites for photogenerated carriers. This will facilitate the recombination of photogenerated electrons and holes at the heterojunction interfaces while suppressing the bulk careers from recombination in both COF and CN_x.[16] Consequently, the remaining holes in the COF VB (−7.64 eV) perform oxidation reactions, while the electrons in the CN_x CB (−4.37 eV) participate in reduction reactions with the help of NiME cocatalyst.

To investigate the intrinsic conductivity, we conducted a liquid-metal contact test using a eutectic Ga–In (75.5:24.5) droplet as a soft top contact on CN_x films. This setup was connected in series with a multimeter to measure resistance. The CN_x film showed no measurable response, effectively behaving as an open circuit. In contrast, the 1:4 (w/w) ITO|CN_x composite film exhibited a clear electrical response corresponding to a resistance of ~1.1 kΩ (Figure S29a, Supporting Information). This result confirms that the inclusion of ITO nanoparticles substantially improves the overall conductivity of the composite film.[17]

To probe the role of ITO during photocatalysis, we compared the photocatalytic H_2 evolution (half-reaction experiment) with CN_x and ITO|CN_x composite (keeping CN_x content same). The CN_x produced 0.43 μmol H_2 in 9.5 h, whereas the ITO|CN_x composite exhibited H_2 evolution of 0.26 μmol (Figure S29b). This decrease suggests that despite improved conductivity from ITO addition, the presence of defect-rich sub-bandgap states in ITO facilitates increased charge recombination in absence of heterojunction, thereby lowering the half-reaction efficiency. However, in composite, ITO acts as mediator between COF and CN_x and increases overall activity. These factors collectively ensure that photogenerated carriers are efficiently separated and preserved at the appropriate bands for driving redox reactions, establishing ITO as a robust electron mediator for the Z-scheme pathway.

Discussion S11. Photopanel and photoleaf for solar reforming.

The photopanel has been constructed on frosted glass with knife casting technique (see Methods section in the manuscript for details). Thus, thickness of the COF|ITO|CN_x composite on the glass is hard to control. The photopanel has been tested through front illumination as well as back illumination. It is very difficult to make a straight forward comparison between them as there are various factors involved during catalysis.

1) Due to the light scattering by the uneven surface of the frosted glass, the transmittance at $\lambda < 450$ nm is constrained significantly.[18]
2) Back irradiation refers to the system where light is illuminated through the back side of the glass on which the photocatalyst is deposited. This offers certain benefit for practical solar reforming and photo reforming demonstration. In this set up, the depth and turbidity of the reaction solution due to substrates does not impact on the light absorption of the photocatalyst.
3) As the loading of catalyst varies from batch to batch, comparing areal activity (μmol m^{-2} h^{-1}) of photopanel in different states (as mentioned earlier) is challenging. During the

synthesis of photocatalyst on glass panel, variation of loading has certain effect on crystallinity and porosity of the material. As loading changes, number of active sites changes within a certain area thereby affecting the overall catalytic properties.

4) The glass is generally covered by a continuous photocatalyst layer. Generally, the targeted loading is 0.5 mg cm^{-2}. Additional loading increases the layer thickness. Although it would enhance the light absorption to some extent, but evolved H_2 amount won't change significantly. As a result, there will be a drop on amount of H_2 production per gm of photocatalyst per h. Again, as the photocatalyst is not transparent, the catalyst deposited at the far end from the illumination direction will have insignificant role in catalysis.
5) In order to make a stand-alone photoleaf, the thickness is as high as ~200 μm (Figure S37a). Thus, surface to mass ratio drops significantly. As a result, activity per area of the photocatalyst is lower in case of photoleaf as compared to the photopanel. On the other hand, the diffusion of substrates and products is limited to one side only in the case of photopanel whereas photoleaf is more porous and diffusion direction is not restricted. Thus, areal activity is higher in the case of photoleaf.

Discussion S12. Choice of cocatalyst during the usage of PET plastic-derived EG as reductant.

The efficiency is generally enhanced during solar reforming of various polymers and biomass in basic medium due to their improved solubility in the medium.[19] Similarly, for real wastes like plastics, highly basic condition will help in their degradation followed by upcycling during solar reforming.[20] However, for a highly efficient system, one of the key components is the cocatalyst. It has been proven that the Ni-based catalysts are often converted to $Ni(OH)_2$ and NiO nanoparticles that participate in the catalysis.[20-21] Thus, in order to perform the solar reforming of PET plastic-derived EG in basic medium, we have replaced the NiME with more robust Pt nano particle. As a proof-of-concept, COF|ITO|CN_x|Pt photopanel has been demonstrated for direct plastic upcycling.

Discussion S13. Stability of the composite during catalysis.

COFs are well-known for their thermal and chemical stability. It is due to the keto-enamine bonding present in this framework, they are highly stable both in acidic and basic medium (Figure S3 and S7b, Supporting Information).[1, 22] The stability of the COF|ITO|CN_x composite has been confirmed by characterizing it after the catalysis (Figure S25). However, a trace amount of CO has been found during solar reforming when reaction bath temperature is not controlled. During solar reforming in a top down and single/multi widow reactor, the temperature of the reaction bath reaches between 45-60 °C and photodecomposition of CN_x occurs that leads to CO generation. However, such observation has not been found during the reaction with controlled temperature at 25 °C.
The *β*-ketoenamine linkages in the COF backbone provide exceptional chemical stability, proven under acidic and basic conditions.[1] However, decomposition of the composite has been

observed with highly basic condition. Before performing the plastic solar reforming, the photopanel was submerged in 1 (M) KOH with PET plastic and then the panel was exposed to simulated sunlight for 24 h without any temperature control. The reaction medium started turning pale yellow after 8 h of reaction which can be seen through naked eyes. The ^{1}H NMR confirms the presence of building blocks (both aldehyde and amine) along with other oligomers (Figure S54 and S55, Supporting Information).

The intramolecular charge transfer promotes tautomerization to form imine bands which under highly basic condition (1 M KOH) breaks down leading to fragmentation of the COF backbone to its building units (*4,4′*-azo dianiline and *2,4,6*-triformylphloroglucinol) and other monomeric units. The NMR reported in the supporting information (Figure S54 and S55, Supporting Information) reveals that the decomposed fragments contain free aldehyde and amine functionalities hinting their source from the COF backbone.

There is further stability issue of the NiME catalyst in highly basic medium as mentioned in the above discussion. As the building units are not active photocatalysts (Table S17, Supporting Information), we tested the plastic solar reforming in presence of Pt nanoparticles without any adjustment of pH.

Table S1. COF yields during synthesis with various composites. During the synthesis of different compositions, *p*-toluene sulphonic acid, *4,4′*-azo dianiline and *2,4,6*-triformylphloroglucinol have been used. Ball-milled CN_x nanoflakes have been considered for CN_x-based compositions. Reaction conditions have been mentioned in detail in the 'Method' section of the manuscript.

Material	COF Yield (%)
COF	74.2 ± 0.8
COF|CN_x	92.9 ± 0.2
COF|ITO|CN_x	91.1 ± 0.8
COF|ITO|CN_x photoleaf	87 ± 0.5
COF|ITO|CN_x photopanel	88.4 ± 0.9

Table S2. Comparison of elemental (CHN) analysis of the individual components and the COF|ITO|CN_x composite. The overall C+H+N sum is essentially identical (62.14% measured vs 61.94% expected), confirming the organic fraction is consistent with the formulation. The slight increase in C and H is attributed to residual *N,N*-dimethylacetamide within the pores, while the marginally lower N content may be due to minor loss of ITO during purification.

Sample	Weight taken (mg)	%C	%H	%N
COF	2.2508	65.06	3.60	16.27
CN_x	2.3453	26.13	1.51	42.27
COF|ITO|CN_x	1.3909	37.49	2.73	21.92

Table S3. Comparison of N_2 adsorption between various components of COF|ITO|CN_x. Pristine COF showcases very high BET surface area whereas incorporation of various components decreases the overall surface area. However, the composite still remains highly porous with considerably high surface area. The samples are preactivated at 120 ºC for 10 h under vacuum before N_2 adsorption measurement.

Material	BET surface area (m^2 g^{-1})	Micro and meso pore volume at P/P_0=0.99
COF	2603	2.19 cm^3 gm^{-1}
CN_x	22	0.08 cm^3 gm^{-1}
COF|ITO|CN_x	555	0.75 cm^3 gm^{-1}
COF|ITO|CN_x photoleaf	315	0.68 cm^3 gm^{-1}

Table S4. T-plot report and comparison of microporous and external surface area (thickness range: 3.5000 Å to 5.0000 Å and thickness equation: Harkins and Jura). (See Discussion S5 for detail information)

	COF	CN_x	CN_x|ITO|COF	CN_x|ITO|COF photoleaf
Slope (cm^3 g^{-1}·Å^{-1} STP)	275.654 ± 24.613	1.266 ± 0.012	34.650 ± 3.307	16.595 ± 1.122
Y-Intercept (cm^3 g^{-1} STP)	-528.829 ± 100.279	0.798 ± 0.052	14.603 ± 13.463	21.975 ± 4.561
Micropore area (m^2 g^{-1})	NA	2.69	17.53	57.64
Micropore volume (cm^3 g^{-1})	-0.8196	0.0012	0.0227	0.0341
External surface area (m^2 g^{-1})	4272.42	19.64	537.45	257.4
Total BET surface area (m^2 g^{-1})	2602.9	22.33	554.98	315

Table S5. Band structure analysis from ultraviolet photoelectron spectroscopy (UPS).

**Photocatalysts**	**Binding energy cut off (eV)**	**Work function (eV vs Vacuum)**	**VBM below Fermi level**	**VBM (eV vs Vacuum)**	**Optical band gap**	**CBM (eV vs Vacuum)**
CN_x	16.39	–4.83	2.18	–7.01	2.64	–4.37
COF	16.09	–5.13	2.51	–7.64	1.95	–5.69

Table S6. Comparison of H_2 evolution between different surfaces of COF|ITO|CN_x|NiME.[a]

**Nature of the surface**	**H_2 activity (μmol g_{cat}^{-1} h^{-1})**
Unmodified	49
Modified (macroporous)	133.9

[a]Reaction condition: COF|ITO|CN_x|NiME (2 mg mL^{-1}), 0.2 M EG in 1 mL water, 600 rpm stirring and irradiation (21 h, AM 1.5 G, 100 mW cm^{-2}, 25 °C).

Table S7. Control experiment varying NiME concentration during solar reforming of EG with COF|ITO|CN_x.[b]

**NiME (nmol mL^{-1})**	**H_2 activity (μmol g_{cat}^{-1} h^{-1})**
10	35.78
7.5	55.29
5	75.44
2.5	82.06
1.25	117.99
0.625	47.16
0.25	27.10

[b]Reaction condition: COF|ITO|CN_x (2 mg), 0.1 M EG in 1 mL water, 600 rpm stirring and irradiation (21 h, AM 1.5 G, 100 mW cm^{-2}, 25 °C). Various amount of NiME solution (in water) was added to the reaction medium and sonicated for 20 minutes followed by stirring at 600 rpm for 2 h before purging with N_2 having 2% methane.

Table S8. Inductively coupled plasma optical emission spectrometry (ICP-OES) quantification of Ni content within COF|ITO|CN_x|NiME composite. The composite was dissolved in nitric acid for the measurement. The loaded weight percentage of Ni is 0.43.

Sample	Raw Average	Ext. Cal. Average
	Y (cps)	Y (ppm)
Blank 2% HNO_3	0.827	0
0.01 PPM	51.055	0.007
0.05 PPM	354.466	0.050
0.1 PPM	709.577	0.101
0.5 PPM	3515.976	0.501
1 PPM	6942.826	0.990
2.5 PPM	17618.511	2.512
5 PPM	35159.882	5.014
7.5 PPM	52458.214	7.481
10 PPM	70165.632	10.006
COF|ITO|CN_x|NiME	297.809	0.042

Table S9. Control experiment with different components of COF|ITO|CN_x composite.[c] The high activity of the composite COF|ITO|CN_x|NiME clearly hints the active participation of individual components during catalysis.

Substrate	H_2 (µmol)	H_2 activity (µmol g_{cat}^{-1} h^{-1})	Error (µmol g_{cat}^{-1} h^{-1})
CN_x|NiME	2.27	56.8	6.9
ITO:CN_x (1:1)|NiME	1.08	27	7.5
COF|NiME	0.05	0.9	0.6
COF:CN_x (1:1)|NiME	1.25	25.9	5.3
COF:CN_x (2:1)|NiME	0.58	12.2	3.3
COF|ITO|CN_x|NiME	6.80	141.7	2.5

[c]Reaction condition: photocatalyst (2 mg mL^{-1}), H_2O as solvent, 0.2 M EG, 600 rpm stirring and irradiation (21 h, AM 1.5 G, 100 mW cm^{-2}, 25 °C). Photocatalysts were pretreated with 1 mL 1.25 µM solution of NiME following standard immobilization method mentioned in the manuscript.

Table S10. Control experiment to investigate the role of various factors during solar reforming of EG using COF|ITO|CN_x composite.[d]

Batch	Catalyst	Cocatalyst	Simulated Light	Substrate	H_2 activity (μmol g_{cat}^{-1} h^{-1})
1	[d']COF|MWCNT|CN_x	NiME	1 sun	EG	61.8
2	COF|ITO|CN_x	NiME	1 sun	—	no
3	COF|ITO|CN_x	NiME	—	EG	no
4	COF|ITO|CN_x	—	1 sun	EG	no
5	—	NiME	1 sun	EG	no
6	COF|ITO|CN_x	NiME	1 sun	EG	133.7
7	COF|ITO|CN_x	NiME	1 sun (420 nm cut off filter)	EG	3.3
8	COF|ITO|CN_x	NiME	1 sun (450 nm cut off filter)	EG	no

[d]Reaction condition: composite (2 mg mL^{-1}), 0.1 M EG in 1 mL water, 600 rpm stirring and irradiation (20 h, AM 1.5 G, 100 mW cm^{-2}, 25 °C). For batch 4, we have used 1.25 nmol of NiME in 1 mL water for the catalysis. Photocatalysts were pretreated with 1 mL 1.25 μM solution of NiME following standard immobilization method mentioned in the manuscript.
[d']COF|MWCNT|CN_x was synthesized following the similar synthetic protocol of COF|ITO|CN_x. A homogeneous paste was prepared by sequentially mixing *p*-toluenesulfonic acid (0.65 mmol, 125 mg), *4,4'*-azodianiline (0.12 mmol, 24 mg), *2,4,6*-triformylphloroglucinol (0.8 mmol, 16 mg), CN_x (36.2 mg, corresponding to the COF formation ratio; see Discussion S2, Supporting Information), and water (100 μL) (see methods in the manuscript). MWCNT nanoparticles (14.4 mg, 0.2 equivalent relative to COF formation) were then incorporated, followed by further grinding with incremental water additions (25 μL×2) to achieve a viscous and uniform paste. The paste was freeze dried for 12 h followed by

Table S11. Control experiment with different ratio of COF, ITO and CN_x within the composite.[e]

Batch	Substrate	H_2 activity (μmol g_{cat}^{-1} h^{-1})
1	COF|$ITO_{0.25}$|CN_x	103.7
2	COF|$ITO_{1.0}$|CN_x	68.2
3	$COF_{(1.5)}$|ITO|$CN_{x(0.5)}$	53.9
4	$COF_{(0.5)}$|ITO|$CN_{x(1.5)}$	96.2

[e]Reaction condition: photocatalyst (2 mg mL^{-1}), H_2O as solvent, 0.2 M EG, 600 rpm stirring and irradiation (4 h, AM 1.5 G, 100 mW cm^{-2}, 25 °C). The standard composite COF|ITO|CN_x actually refers to COF|$ITO_{0.5}$|CN_x. Photocatalysts were pretreated with 1 mL 1.25 μM solution of NiME following standard immobilization method mentioned in the manuscript.

Table S12. Measurement of External quantum efficiency (EQE) using various photocatalyst during solar reforming.[f] The EQE measurement showcases the critical role of ITO as electron mediator. The performance drops when CN_x content in the reaction medium is halved. Significant jump in EQE when composites have been used. This result is also consistent with the control experiment using simulated sunlight (AM 1.5 G, 100 mW cm^{-2}, 25 °C) mentioned in Fig 3a and Table S8.

Catalyst	H_2 (µmol)	% EQE
CN_x|NiME	0.29	0.031
COF:CN_x (1:1)|NiME	0.13	0.014
COF|ITO|CN_x|NiME	0.93	0.102

[f]Reaction condition: photocatalyst (4 mg mL^{-1}), NiME = 1.25 nmol mL^{-1}, H_2O as solvent, 0.1 M EG, 500 rpm stirring and irradiation for 24 h with a monochromatic light at a wavelength of 400 nm having full width at half-maximum (FWHM) of 15 nm. Light intensity is of approximately 6.3 mW cm^{-2}.

Table S13. Long-term durability test of the COF|ITO|CN_x|NiME composite during solar reforming of EG.[g]

Time (h)	H_2 (µmol)	Error (µmol)	H_2 activity (µmol g_{cat}^{-1} h^{-1})	Error (µmol g_{cat}^{-1} h^{-1})
2	0.225	0.041	56.24	10.28
4	0.674	0.049	84.26	6.15
5.5	1.05	0.106	95.42	9.65
17.5	4.478	0.323	127.93	9.23
20	5.359	0.339	133.96	8.47
24 (Day 1)	6.804	0.124	141.76	2.58
41.25	12.929	0.389	156.71	4.71
50	15.883	0.685	158.83	6.84
62.25	20.841	0.818	167.39	6.57
72 (Day 3)	24.365	0.507	169.2	3.52
90	29.731	0.67	165.17	3.72
114.75	36.9	1.255	161.04	5.46
120 (Day 5)	38.3	1.914	159.66	7.97
135[g’]	41.9	0.402	155.26	1.49
152.5	56.4	4.958	184.94	16.25
168 (Day 7)	71.86	3.581	213.86	10.65

[g]Reaction condition: COF|ITO|CN_x|NiME (2 mg mL^{-1}), 0.5 M EG, 600 rpm stirring, and irradiation (AM 1.5 G, 100 mW cm^{-2}, 25 °C). At various time interval, gas from the head space was taken for quantification.

[g’]cocatalyst addition: 1.25 nmol of NiME in 50 µL water was added at the 132nd h of the reaction (Discussion S7).

Table S14. Quantification of formate through ion chromatography after 7 days of reaction using COF|ITO|CN_x|NiME photocatalyst.[h]

Batch	Formate (µmol)	Mean formate content (µmol)	Error in formate quantification (µmol)	Formate activity (µmol g_{cat}^{-1} h^{-1})	Mean formate content (µmol g_{cat}^{-1} h^{-1})	Error in formate activity (µmol g_{cat}^{-1} h^{-1})
1	40.3	34.8	4.8	120.1	103.7	14.4
2	33			98.2		
3	31			92.9		

[h]Reaction condition: COF|ITO|CN_x|NiME (2 mg mL^{-1}), 0.5 M EG, 600 rpm stirring, and irradiation (AM 1.5 G, 100 mW cm^{-2}, 25 °C). Formate was quantified after 7 days of reaction (cocatalyst addition: 1.25 nmol of NiME in 50 µL water was added at the 132nd h of the reaction).

Table S15. Substrate scope during solar reforming using COF|ITO|CN_x|NiME.[i] The composite showcases solar reforming activities by producing H_2 coupled with selective oxidation of various substrates to formate. Relatively lower activity during sugars oxidation could be due to the poor mass transport as compared to the smaller polyols.

Substrate	H_2 activity (µmol g_{cat}^{-1} h^{-1})	Error (µmol g_{cat}^{-1} h^{-1})	Formate activity (µmol g_{cat}^{-1} h^{-1})	Error (µmol g_{cat}^{-1} h^{-1})
EG	107.4	9.1	57.2	4.6
Glycerol	72.6	2.2	28.2	4.1
Glucose	5.5	0.6	4.8	0.4
Fructose	2.9	0.2	2.5	0.5
Arabinose	3.3	0.2	3.3	0.6
Galactose	2.6	0.3	2.9	0.6

[i]Reaction condition: COF|ITO|CN_x|NiME (2 mg mL^{-1}), 0.1 M substrate, 600 rpm stirring, and irradiation (AM 1.5 G, 100 mW cm^{-2}, 25 °C).

Table S16. Comparison of solar reforming performances between a standalone photoleaf and glass supported photopanel.[j]

Processability	Mass to surface ratio (mg cm^{-2})	H_2 (μmol)	Activity (μmol g_{cat}^{-1} h^{-1})	Area (cm^2)	Areal H_2 efficiency (μmol m^{-2} h^{-1})
Standalone photoleaf	2.27	1.4	34.9	0.88	662.9
Glass supported Panel	0.32	10.8	56.2	25	215.6

[j]Reaction condition: NiME|CN_x|ITO|COF photopanel, H_2O as solvent, 0.5 M EG, irradiation (21 h, AM 1.5 G, 100 mW cm^{-2}, 25 °C).

Table S17. Product quantification during solar reforming using a multipanel reactor having active area of 54.76 cm^2.[k]

Product	Amount (μmol)	Error (μmol)	Areal H_2 efficiency (μmol m^{-2} h^{-1})	Error (μmol g_{cat}^{-1} h^{-1})
Hydrogen	29.8	2.6	272.2	23.8
Formate	15	1.4	137.2	12.4

[k]Reaction condition: NiME|CN_x|ITO|COF photopanel, H_2O as solvent, 0.5 M EG, irradiation (20 h, AM 1.5 G, 100 mW cm^{-2}, 25 °C).

Table S18. Control experiment with the building units of COF.[l] Solar reforming was performed with the building units in 1 (M) KOH to check if the decomposition products from panel as any photocatalytic activity.

Photocatalyst	Cocatalyst	Simulated Light	Substrate	H_2 (μmol)
2,4,6-Triformylphloroglucinol	NiME	1 sun	EG	n.d.
4,4′-azo dianiline	NiME	1 sun	EG	n.d.
2,4,6-Triformylphloroglucinol	Pt	1 sun	EG	n.d.
4,4′-azo dianiline	Pt	1 sun	EG	n.d.
2,4,6-Triformylphloroglucinol and *4,4′*-azo dianiline (1:1 wt ratio)	Pt	1 sun	EG	n.d.

[l]Reaction condition: aldehyde and amine (2 mg mL^{-1}), NiME = 1.25 nmol mL^{-1}, 5wt% of Pt nanoparticle, 1 (M) KOH, 0.5 M EG, 600 rpm stirring and irradiation (20 h, AM 1.5 G, 100 mW cm^{-2}, 25 °C).

Table S19. Hydrogen production with a photopanel of 25 cm^2 using direct sun light in real world scenarip.[m]

Product	Amount (μmol)	Error (μmol)	Areal H_2 efficiency (μmol m^{-2} h^{-1})	Error (μmol m^{-2} h^{-1})
Hydrogen	2.16	0.22	108.2	10.9
Formate	0.43	0.02	21.8	1

[m]Reaction condition: Pretreated plastic in 1M KOH, irradiation under direct sunlight (8 h) and 5wt% of K_2PtCl_6 was used for photodeposition.

Table S20. Comparison of photo/solar reforming performance between different CN_x-based panels for H_2 production.[n]

Photocatalyst	Panel size	Substrate	Condition	Areal H_2 efficiency (μmol m^{-2} h^{-1})	Ref.
COF|ITO|CN_x|NiME photoleaf	0.88 cm^2	EG	Under water	662	This work
COF|ITO|CN_x|NiME panel	25 cm^2	EG	front	215	
COF|ITO|CN_x|NiME panel	54.76 cm^2	EG	back	272	
COF|ITO|CN_x|Pt panel	25 cm^2	Pretreated PET plastic	Front, natural sunlight	108.2 ± 10.9	
Mpg-CN_x|Pt	0.756 m^2	TEOA		9028	[23]
MCA-CN_x-Pt	6.7 × 6.7 cm^2	Ethanol	front	584	[24]
CN_x|Ni_2P	25 cm^2	cellulose	back	38	[20]
		MSW	back	130	
		PET	back	52	

[n]Unless stated otherwise in the "condition" column, measurements were performed in pure H_2O under simulated sunlight (100 mW cm^{-2}) at 25 °C and ambient pressure; TEOA=triethanolamine and MSW= municipal solid waste

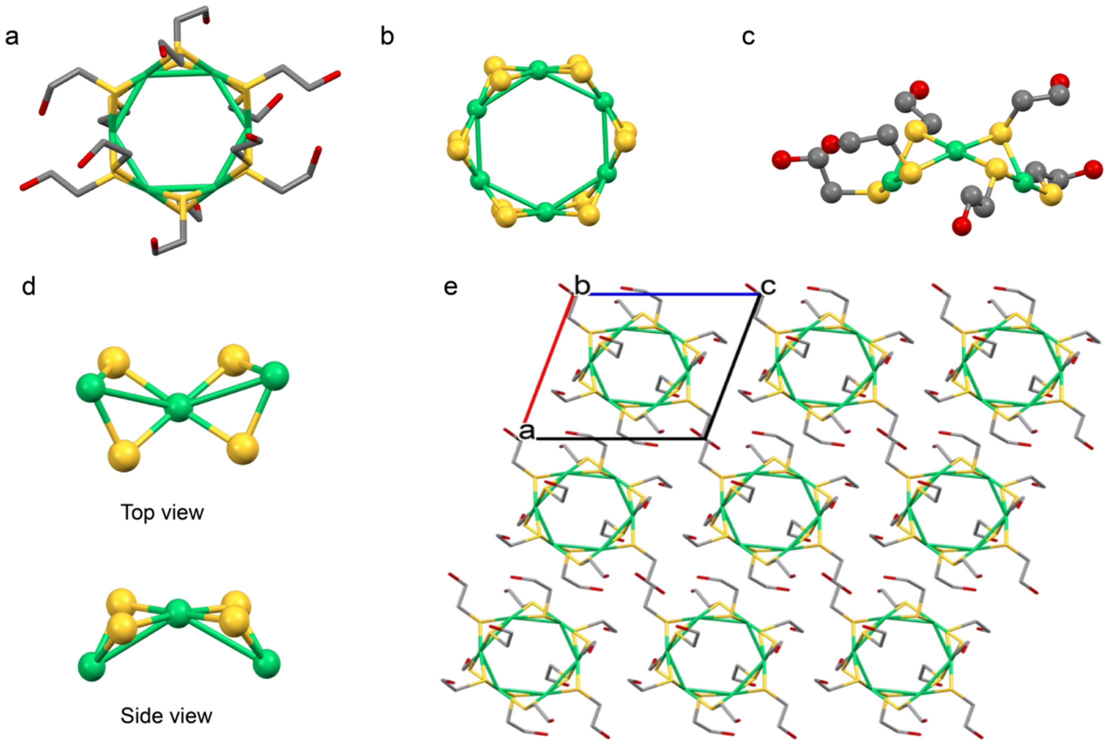

Figure S1. Single crystal X-ray diffraction structure of NiME cocatalyst. a) capped stick model of the complex. H-atoms in the aliphatic chains have been omitted for general representation. b) Ball and stick model of hexanucelar structure. Only Ni center and its surrounded atoms have been considered for simplicity. c) Atomic connectivity of the complex (H-atoms are omitted for simplistic representation purpose). d) Different orientations of the geometry of a Ni center with surrounded S-atoms in the NiME complex. e) Unit cell packing of NiME complex. The packing suggests the *a (alpha)* form of the complex.[25] The crystal has been grown by vapour diffusion method using methanol and water.

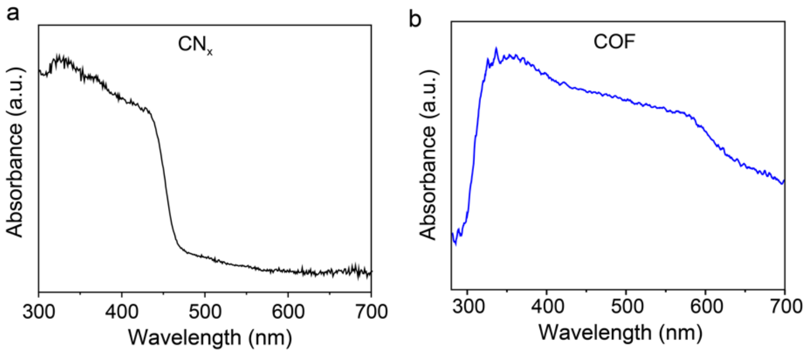

Figure S2. Diffuse reflectance UV–vis spectrum of a) CN_x and b) COF. The spectrum confirms that the COF has light absorption in the full visible region and together they can absorb in the range of UV to visible.

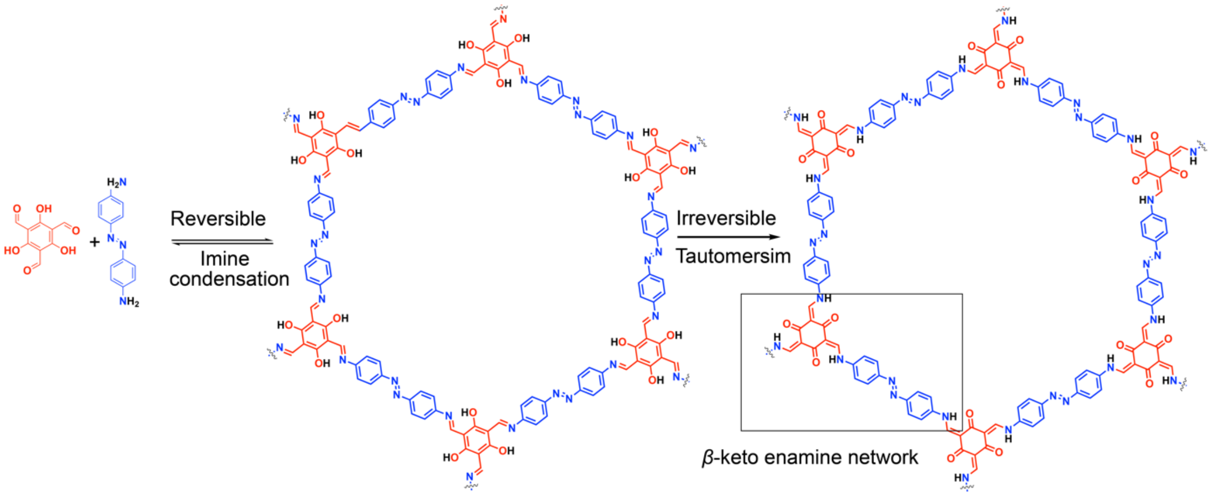

Figure S3. Construction of COF from the building unit. During the solid-state synthesis, imine bonds are first formed *via* Schiff-base reaction. Then, keto–enol tautomerism leads to the chemically stable COF structure. The first step of this COF synthesis reaction, i.e., *s-cis*-imination is a reversible reaction where error correction occurs. Following the dynamic covalent chemistry, the high degree of reversibility imparts high crystallinity to the system. The following irreversible reaction (formation of *β*-keto-enamine bonds) results in high chemical stability of the COF.

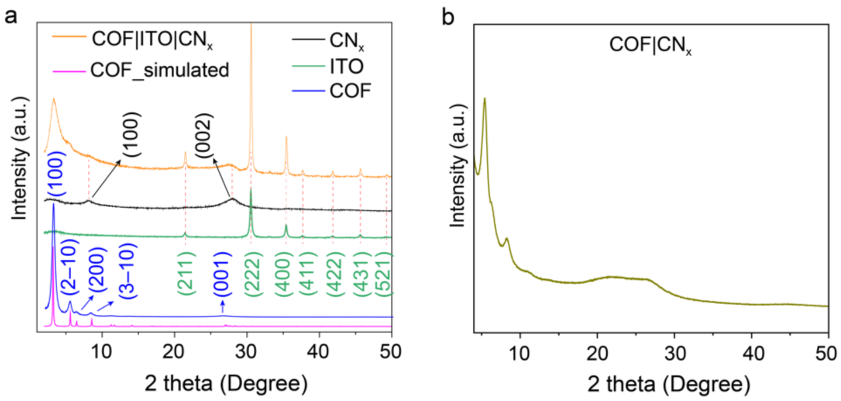

Figure S4. PXRD pattern of different components of COF|ITO|CN_x composite. a) Comparison of diffraction patterns of different components. b) PXRD pattern of COF|CN_x composite. The *d*-spacing between the COF layers is 3.4 Å. There are possibilities of multiple types of stackings: 1. intercalation of the CN_x layer between two COF layers; 2. stacking between individual CN_x layers; 3. stacking of COF layers and 4. stacking between layered COF and layered CN_x. Due to the presence of different types stacking modes in the composite, inter layer distances varies and peak broadening occurs.

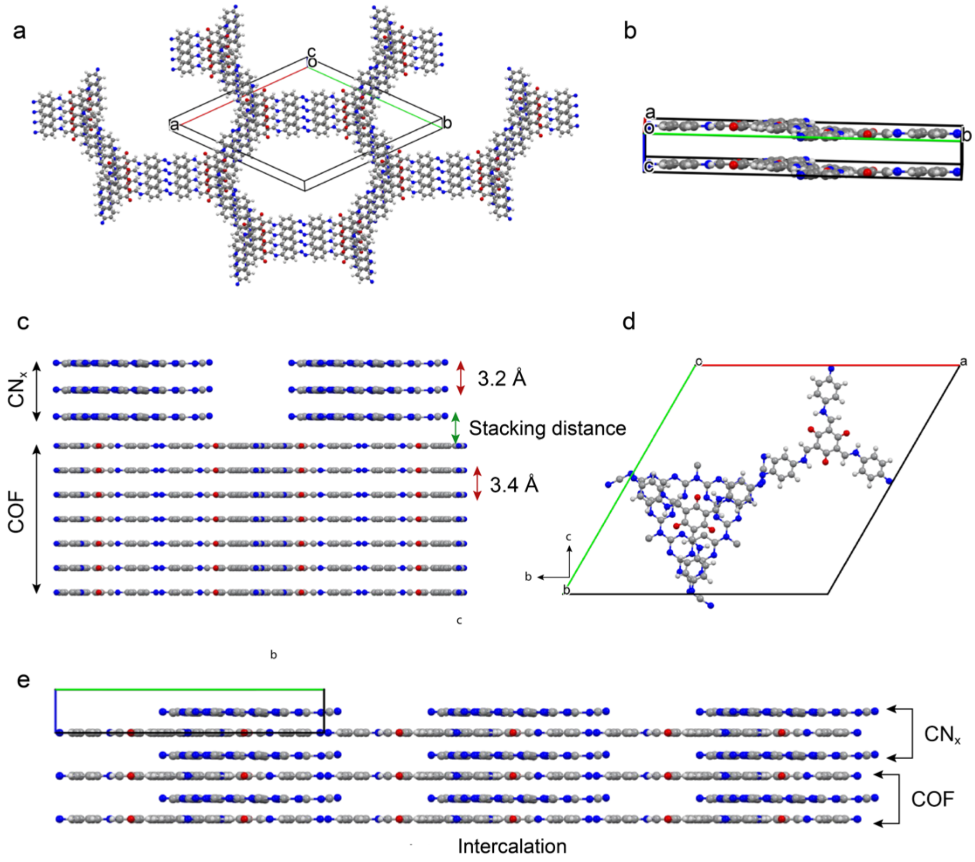

Figure S5. Assembly of CN_x and COF. a) Side view of a stacking mode where COF layer and CN_x layer stacks. Based on the number of COF and CN_x layers, the stacking distance will vary as they are stacked by noncovalent interactions. b) Top view of the stacking of single COF and

CN_x layer and c) intercalation of a CN_x layer between two COF layers. The intercalation distance between a CN_x layer and a COF layer is 2.2 Å as calculated from the PXRD pattern. The structure has been optimized using Material studio software with Density Functional Tight-Binding (DFTB) method.[26]

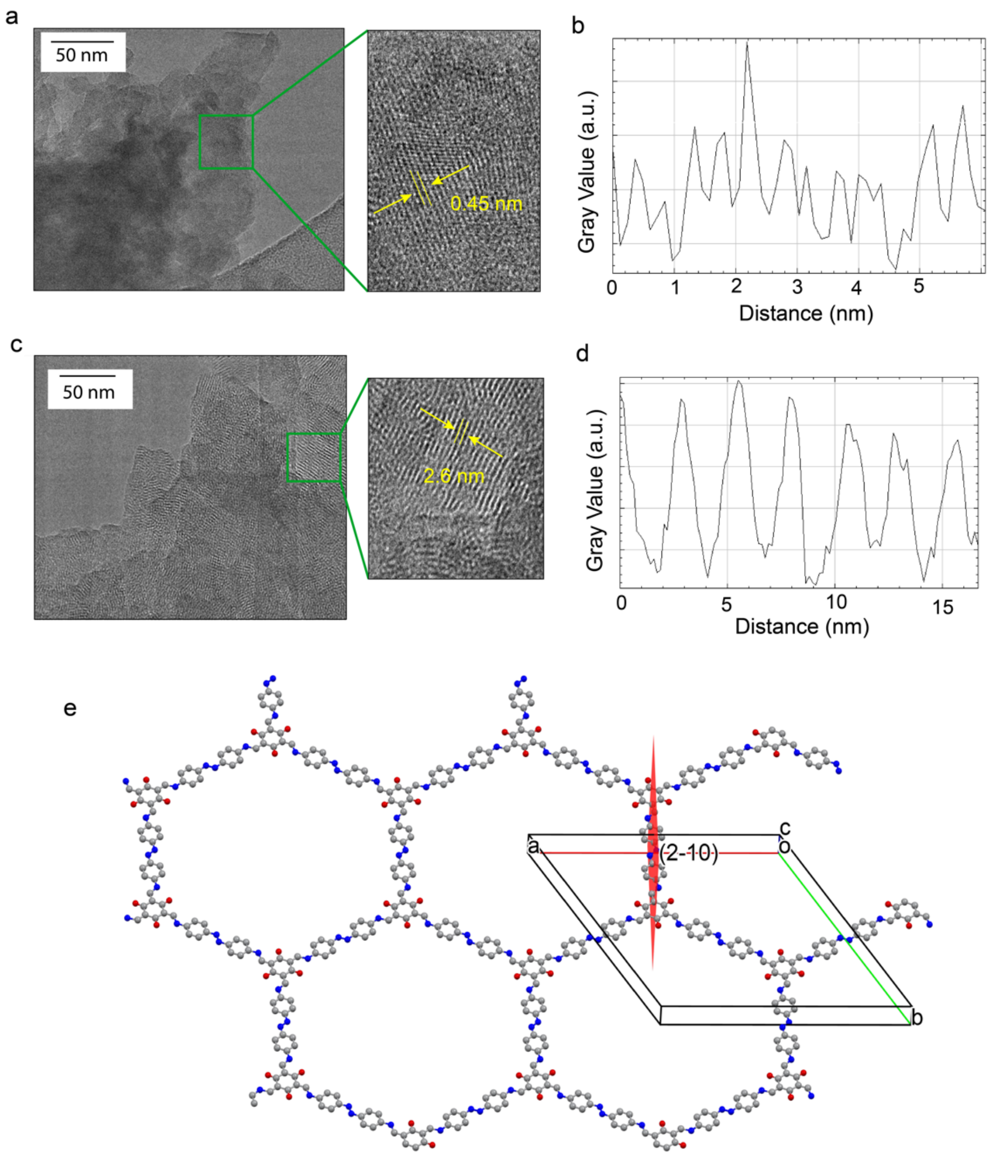

Figure S6. High-resolution transmission electron microscopy imaging of COF|ITO|CN_x composite. a) HRTEM images of COF|ITO|CN_x composite showcasing fringes for (001) planes. b) Plot profile of the selected area of TEM image a. c) HRTEM images of COF|ITO|CN_x composite showcasing fringes for (2–10) planes. d) Plot profile of the selected area of TEM image c. e) Extended ball and stick model of COF highlighting (2−10) plane.

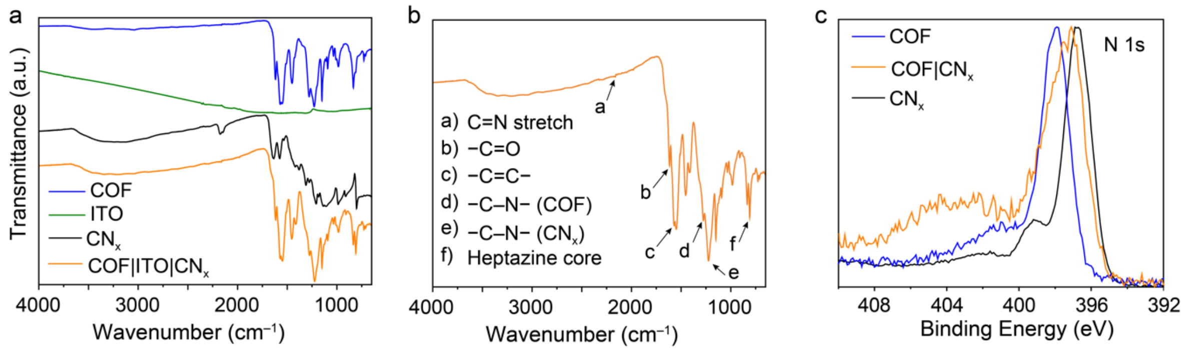

Figure S7. Spectroscopic comparison of the components in COF|ITO|CN_x. a) Comparison of IR spectrum between different components of COF|ITO|CN_x. The spectrum highlights the key peaks referred to the composition of the material. Presence of COF and CN_x has been ascertained. b) IR spectrum of COF|ITO|CN_x highlighting key peaks from each component. The IR spectrum of ITO in the mid-IR range (4000–650 cm^{-1}) is very weak due to low lattice vibration intensity, and its signals are largely masked by those of COF and CN_x. c) X-ray photoelectron spectra of N 1s for the edges for the COF, COF|CN_x and CN_x.

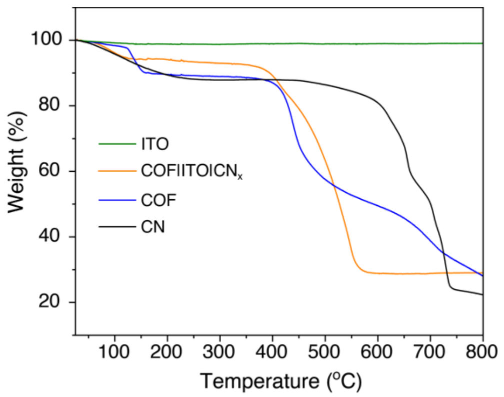

Figure S8. Comparison of thermo gravimetric analysis between different components of COF|ITO|CN_x. The initial weight loss after 100 °C is due to the evaporation of encapsulated solvents used for the purification purposes.

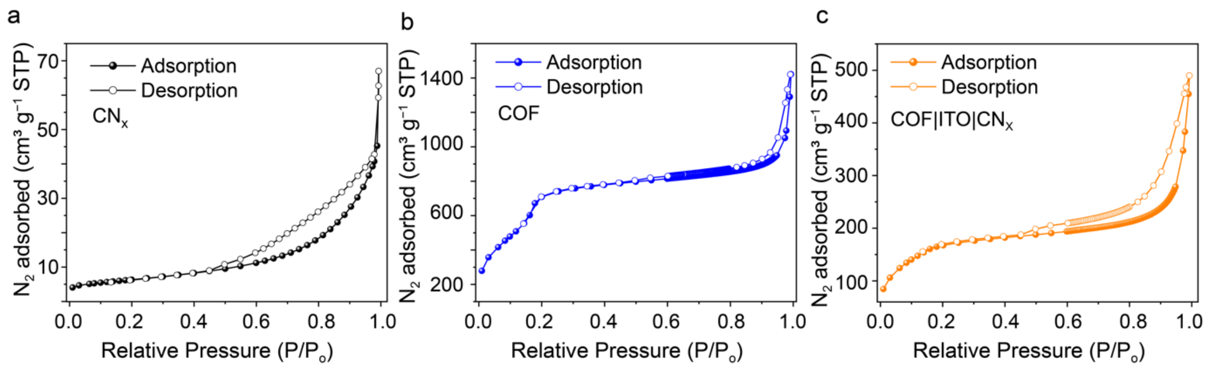

Figure S9. N_2 isotherm of a) CN_x b) COF and c) COF|ITO|CN_x measured at 77.5 K. The BET surface area has been mentioned in Table S2 (Supporting Information). The decrease in overall BET surface area of the COF|ITO|CN_x composite is due to the incorporation of CN_x nanoflakes in to the COF layers. The pore blockage is also due to the presence of ITO nanoparticles in the matrix thereby decreasing the surface area. The hysteresis in the composite isotherm clearly hints the hierarchical porous nature of the composite. The samples are preactivated at 120 ºC for 10 h under vacuum before N_2 adsorption measurement.

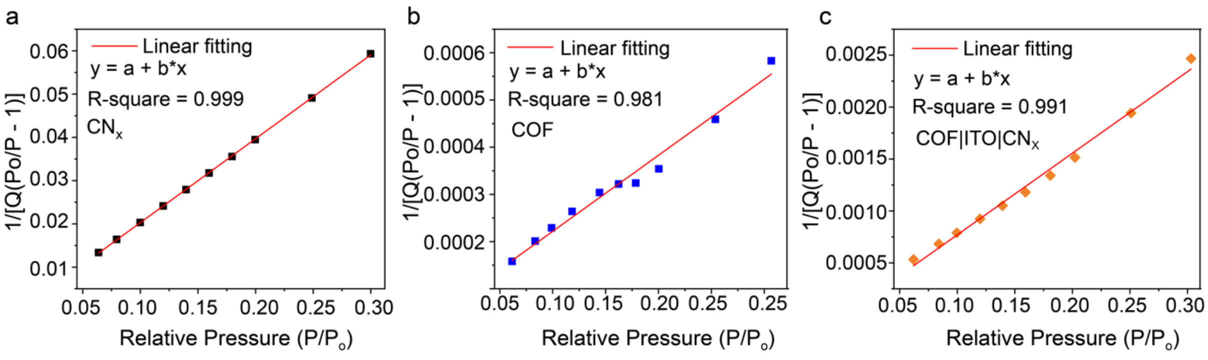

Figure S10. Fitting curve for the BET surface area measurement at low relative pressure (0.05-0.3) measured at 77.5 K. The actual BET surface area has been calculated from this low-pressure region considering the monolayer adsorption of N_2.

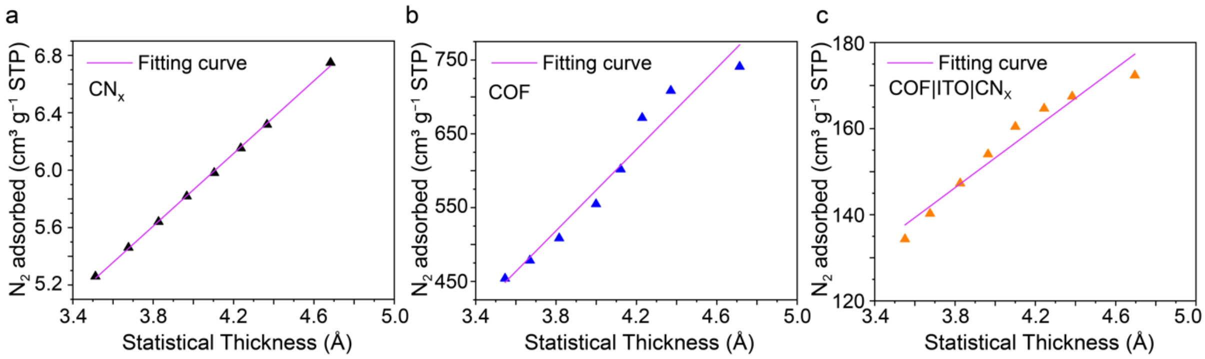

Figure S11. t-Plot for different porous materials a) CN_x b) COF and c) COF|ITO|CN_x measured at low thickness range (3-5 Å) measured at 77.5 K. The t-plot measurement provides the key information of microscopic and external (from meso- and macropores) surface area. The negative slope for COF refers to the absence of any micropores in the structure that is obvious from its mesoporous structure. COF|ITO|CN_x composite's micropores have been generated by the incorporation of CN_x only. Details of surface area and pore volume calculations have been mentioned in Discussion S5.

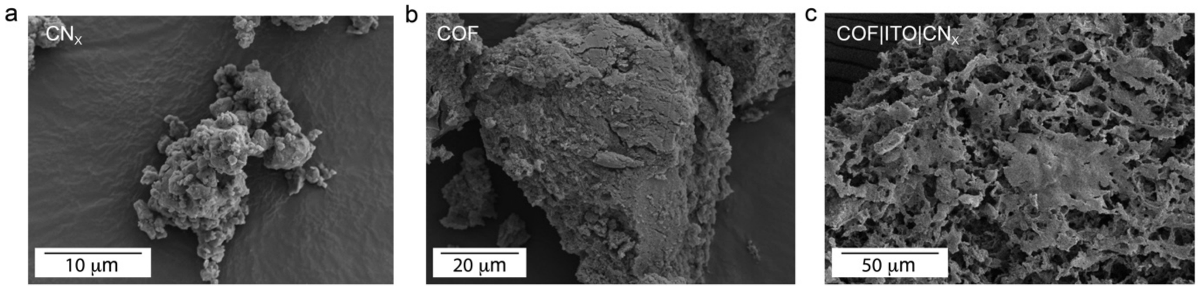

Figure S12. Comparison of scanning electron microscopy images. CN_x does not possess any uniform morphology whereas COF has aggregated structure of two-dimensional sheets. This COF morphology is common when salt-mediated crystallization process is followed. CN_x|ITO|COF composite has unique morphology where macropores have been created on two-dimensional sheets following the freeze drying technique.

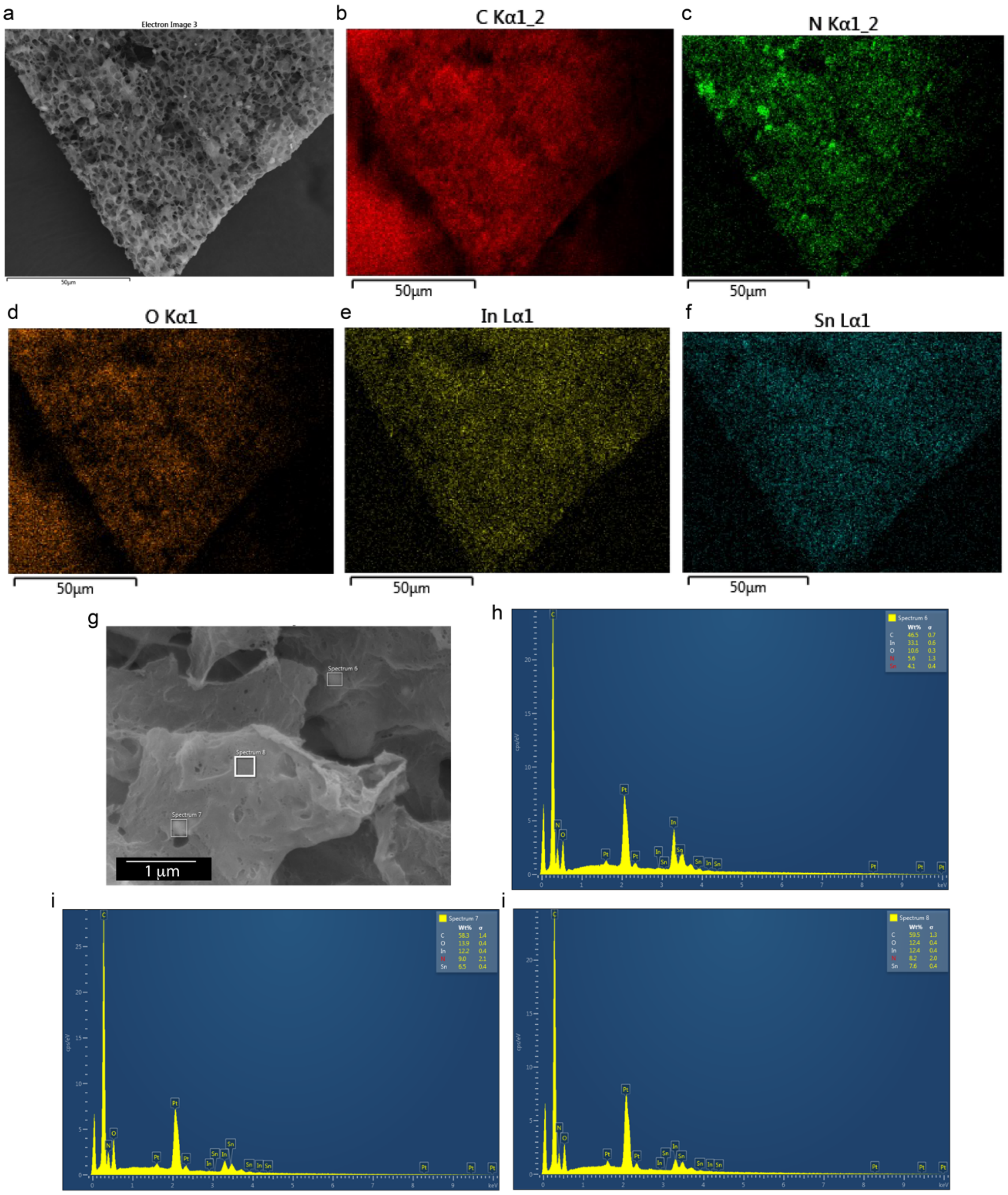

Figure S13. SEM–EDX elemental mapping of COF|ITO|CN_x composite. a–e) EDX elemental mapping image images highlight the composition and uniform distribution of C, N, O, In and Sn within the matrix. g–j) SEM–EDX elemental mapping spectra of COF|ITO|CN_x composite. The measured atomic ratios don't exactly match the bulk composition due to inherent limitations of surface-sensitive EDX analysis and the composite's heterogeneous structure.

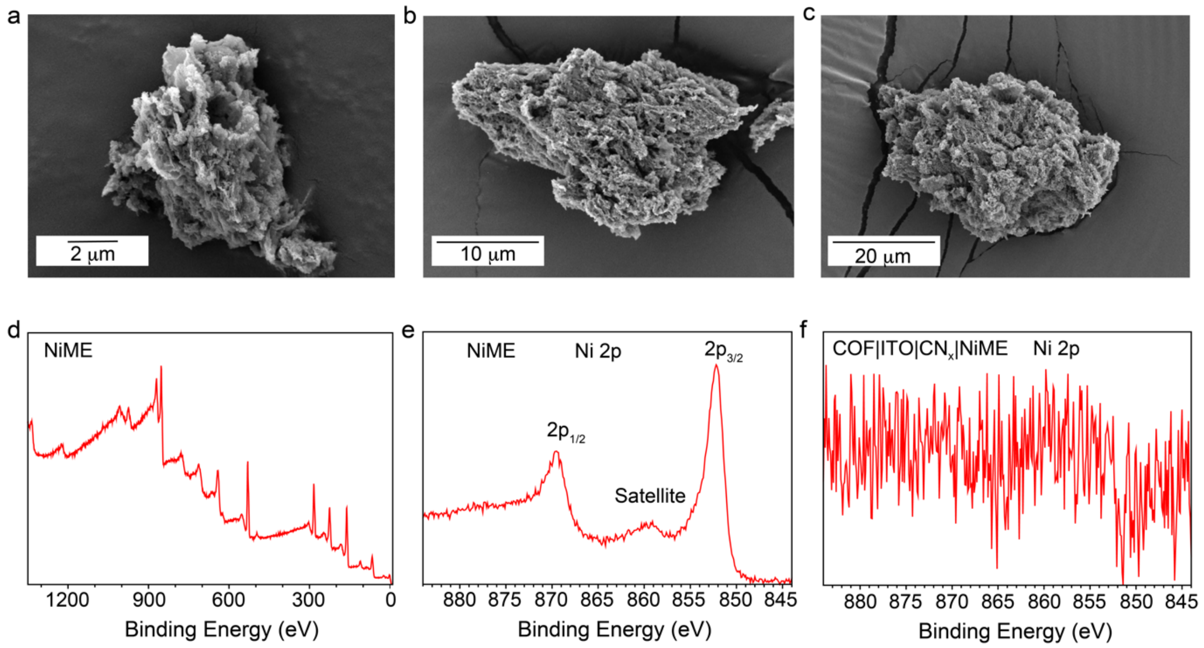

Figure S14. Characterization of NiME cocatalyst. a–c) SEM images of the COF|ITO|CN_x|NiME powder referring their particle sizes. d,e) X-ray photoelectron spectra of NiME. f) XPS of COF|ITO|CN_x|NiME composite at the Ni 2p region.

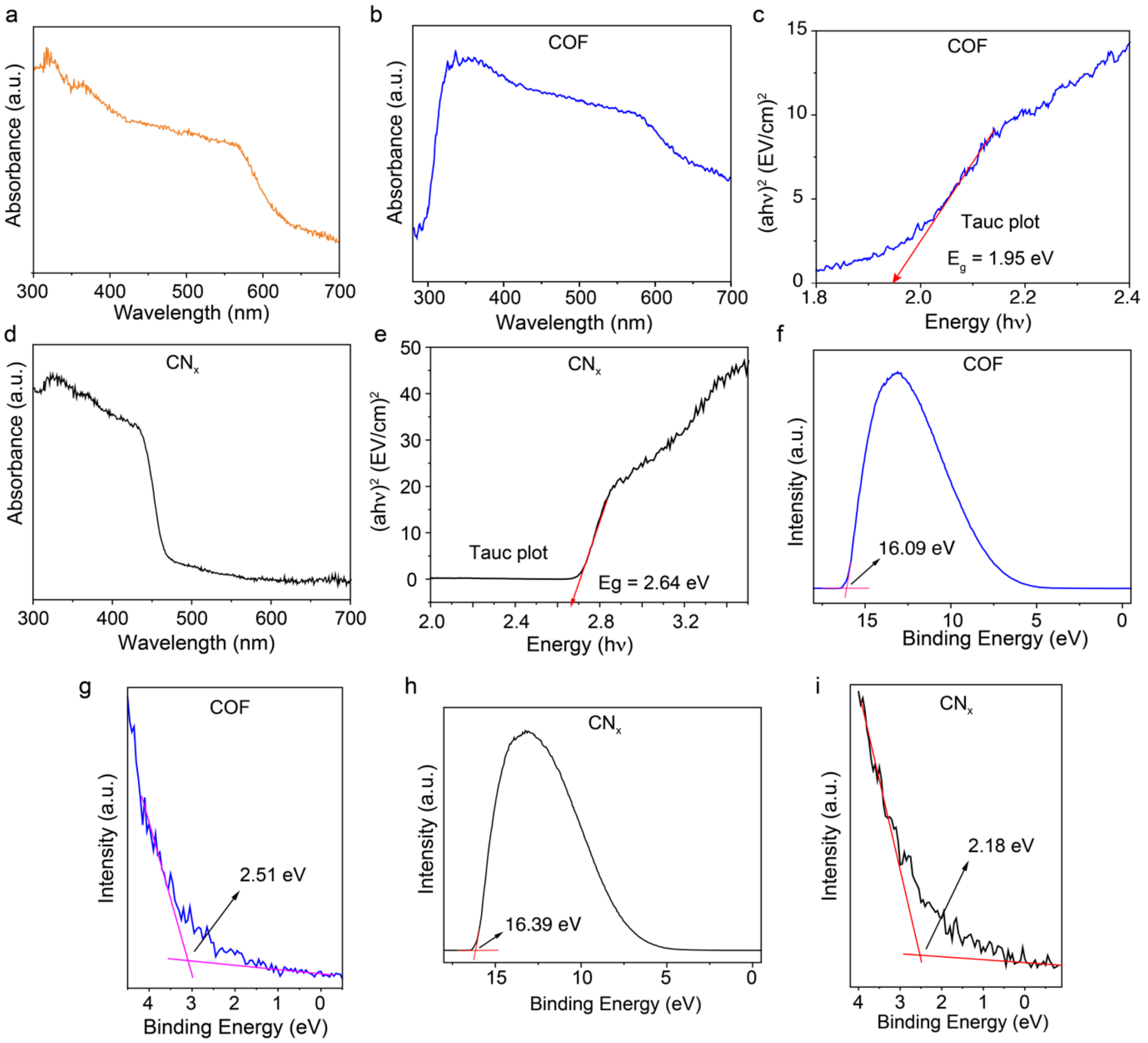

Figure S15. UV–vis and ultraviolet photoelectron spectroscopy (UPS). a) Diffuse reflectance UV–vis spectrum of COF|ITO|CN_x. b) Diffuse reflectance UV–vis of COF. b) Tauc plot derived from the UV–vis spectrum of COF. Plots provides the bandgap of 1.95 eV. c) Diffuse reflectance UV–vis of CN_x. d) Tauc plot derived from the UV–vis spectrum of CN_x. Plots provides the bandgap of 2.64 eV. e–i) UPS spectra (He−Iα=21.22 eV) corresponding to valence band (VB) and cutoff regions of the pristine COF and CN_x.

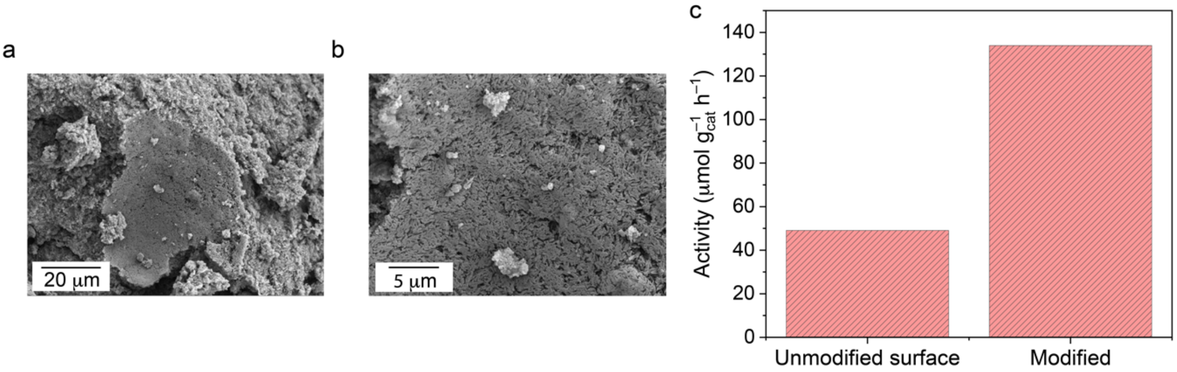

Figure S16. Surface modulation of COF|ITO|CN_x composite and activity comparison. a,b) SEM image of unmodified surface highlighting layered structure and absence of large macropores of μm size. **c)** Comparison of hydrogen evolution activity between unmodified and modified surfaces of COF|ITO|CN_x|NiME (Reaction conditions: COF|ITO|CN_x|NiME (2 mg mL^{-1}), 0.2 M EG in 1 mL water, 600 rpm stirring and irradiation (21 h, AM 1.5 G, 100 mW cm^{-2}, 25 °C). The modified surface possesses macropores that offers faster mass transfer and better access of the reductant to the photocatalyst (closer to the cocatalyst center).

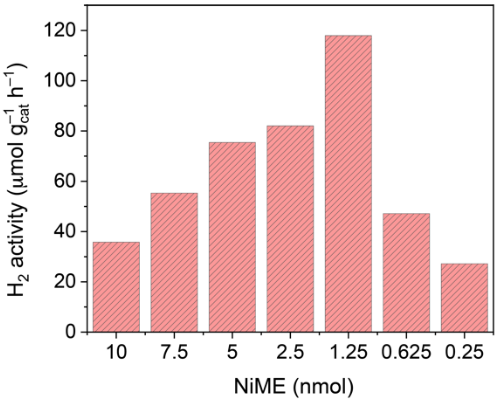

Figure S17. Hydrogen activity with varying NiME concentration using COF|ITO|CN_x photocatalyst (Reaction conditions: COF|ITO|CN_x (2 mg), 0.1 M EG in 1 mL water, 600 rpm stirring and irradiation (21 h, AM 1.5 G, 100 mW cm^{-2}, 25 °C). Various amount of NiME solution (in water) was added to the reaction medium and sonicated for 20 minutes before purging with N_2 having 2% methane. At higher cocatalyst concentration the activity drops significantly due to the significant parasitic light absorption of NiME thereby hindering the photoactivity of the composite. At lower cocatalyst concentration, the performance decreases as the active catalytic centers are significantly low in number.

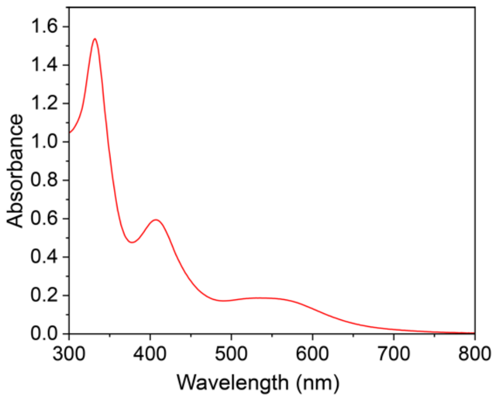

Figure S18. UV–vis spectrum of NiME.

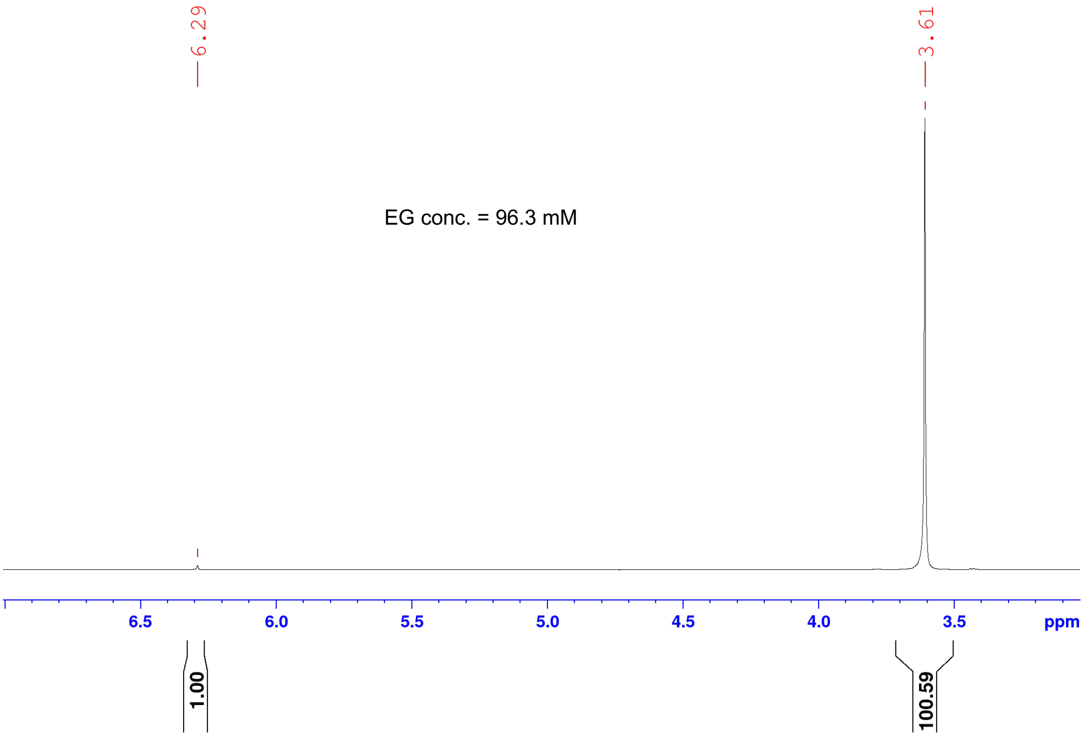

Figure S19. ^{1}H NMR spectrum (D_2O, 400 MHz) for the measurement of EG concentration in the reaction medium after COF|ITO|CN_x treatment. 100 mM aqueous EG solution was treated with 2 mg of COF|ITO|CN_x composite and then sonicated for 15 mins at room temperature and purged with 2% methane in N_2 to mimic the prior to solar reforming condition. The composite shows EG adsorption capacity of 1.85 mmol g^{-1}. Maleic acid (1.53 mM) has been used as internal standard.

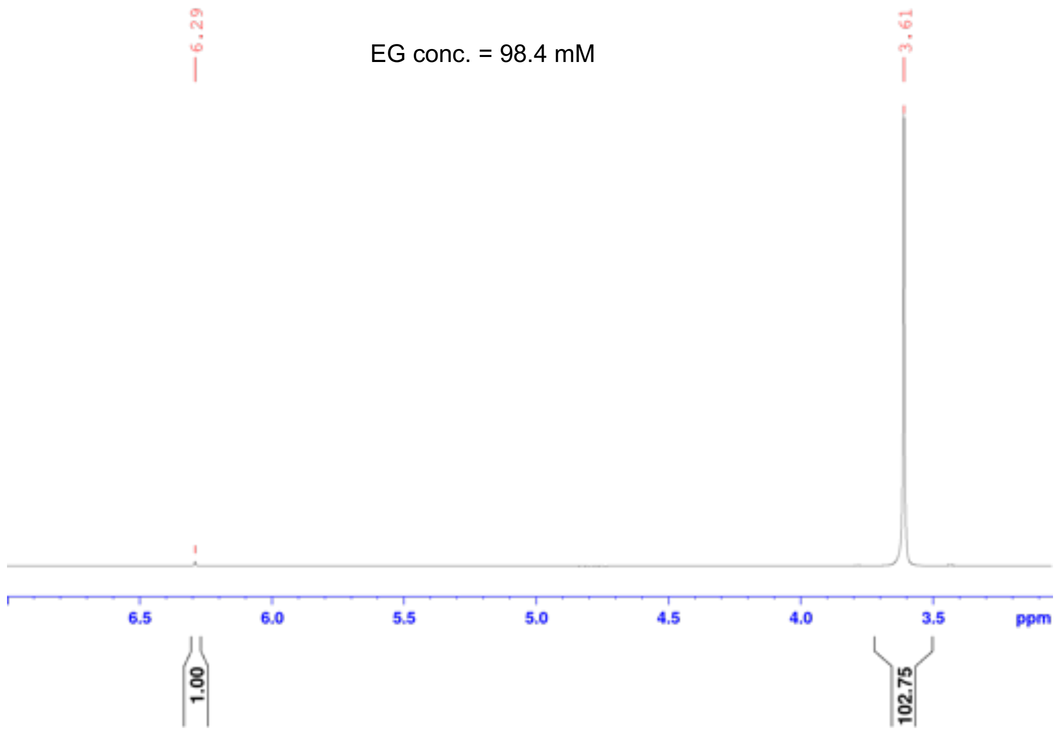

Figure S20. ^{1}H NMR spectrum (D_2O, 400 MHz) for the measurement of EG concentration in the reaction medium after COF|ITO|CN$_x$|NiME (standard composite used for solar reforming having 12.1 nmol NiME per mg COF|ITO|CN$_x$) treatment. 100 mM aqueous EG solution was treated with 2 mg of COF|ITO|CN$_x$|NiME composite and then sonicated for 15 mins at room temperature and purged with 2% methane in N_2 to mimic the prior to solar reforming condition. The composite shows EG adsorption capacity of 0.8 mmol g^{-1}. Maleic acid (1.53 mM) has been used as internal standard.

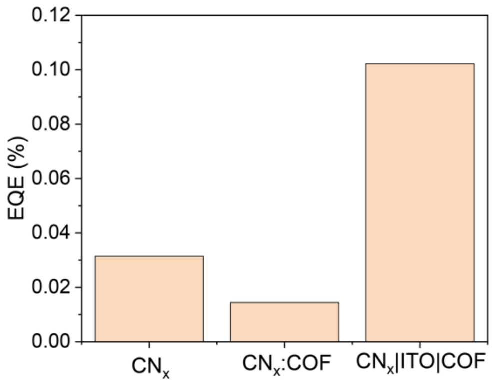

Figure S21. External quantum efficiency (EQE) measurements. The EQE value increased almost 3 times when COF|ITO|CN$_x$ has been used instead of CN$_x$. This finding justifies COF's role both as light absorber and photocatalyst. Reaction conditions: photocatalyst (4 mg mL^{-1}), NiME = 1.25 nmol mL^{-1}, H_2O as solvent, 0.1 M EG, 500 rpm stirring and irradiation for 24 h with a monochromatic light at a wavelength of 400 nm having full width at half-maximum (FWHM) of 15 nm. Light intensity is of approximately 6.3 mW cm^{-2}. EQE was calculated using the equation mentioned in method section.

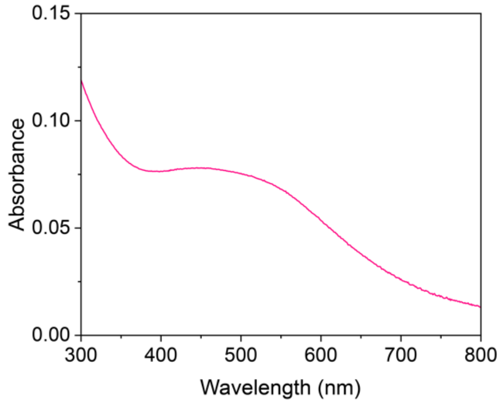

Figure S22. UV–vis spectrum of NiME after irradiation of 72 hrs. The bare NiME cocatalyst decomposes in water medium after 72 h of irradiation (1.5 G, 100 mW cm^{-2}, 25 °C).

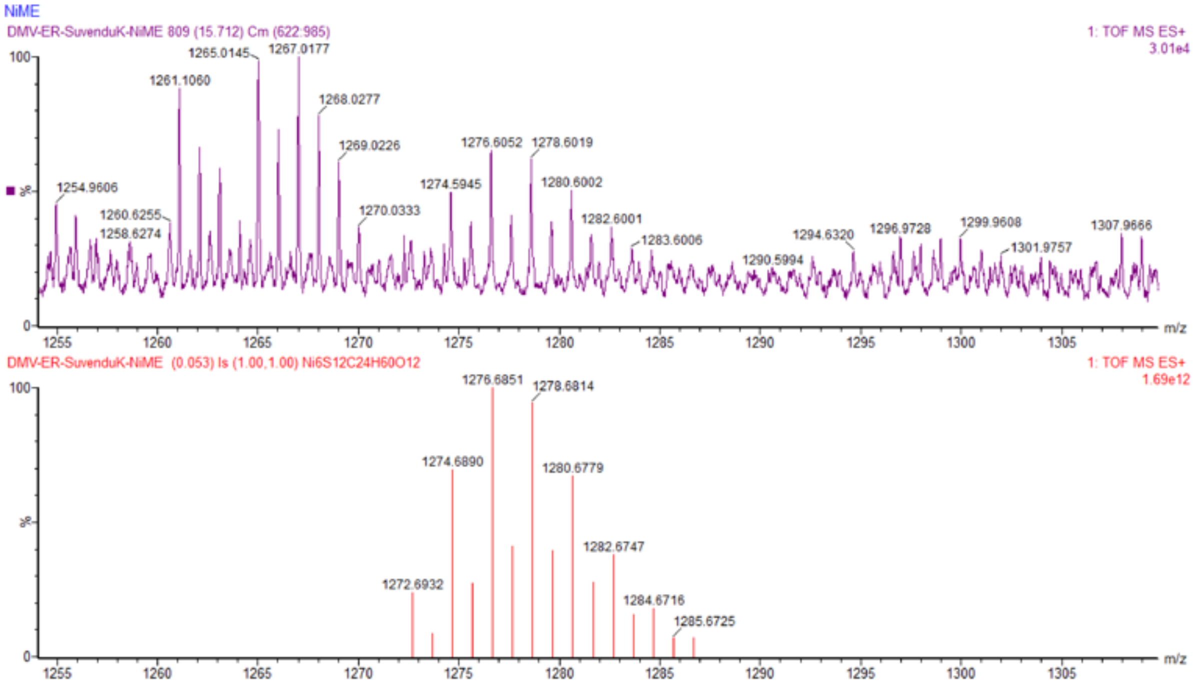

Figure S23. Mass spectrum of NiME complex confined inside the nanopores of the composite. COF|ITO|CN_x|NiME was exposed for 72 h of irradiation in water medium. The composite was then washed with acetone and ethanol; the filtrate was characterized through mass spectrometry. The spectrum confirms that the hexameric structure of the NiME complex remains intact inside the nanopores of the composite even after 3 days of photocatalysis.

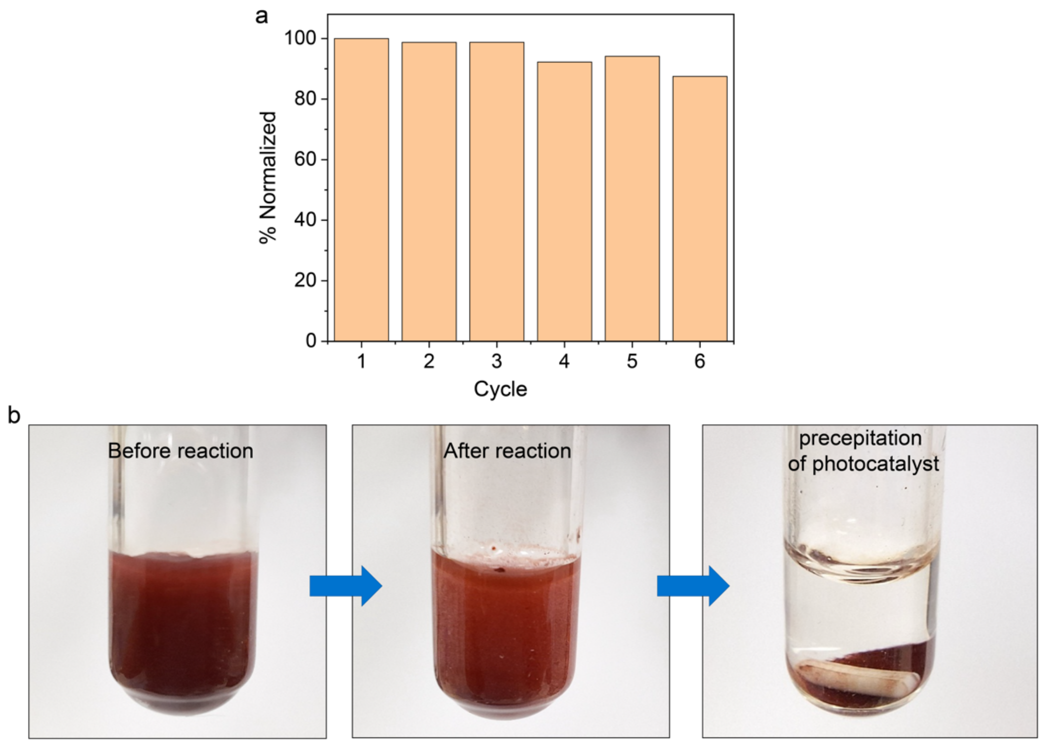

Figure S24. Recyclability test of COF|ITO|CN_x|NiME composite. a) The change in H_2 production activity throughout the cycles (normalised to the first cycle performance) as observed during solar reforming of EG. The little drop in performance is due to the human error during sample recovery and activation (reaction conditions: COF|ITO|CN_x|NiME (1 mg mL^{-1}), 0.1 M EG, 600 rpm stirring, and irradiation (AM 1.5 G, 100 mW cm^{-2}, 25 °C). b) Various steps of photocatalyst recycle. Catalyst is recovered by centrifuge followed by washing with water and acetone to remove deposited cocatalyst and any substrate absorbed. Then the composite is activated by heating at 120 °C under vacuum for 10 h. NiME cocatalyst is reloaded before every set of reaction.

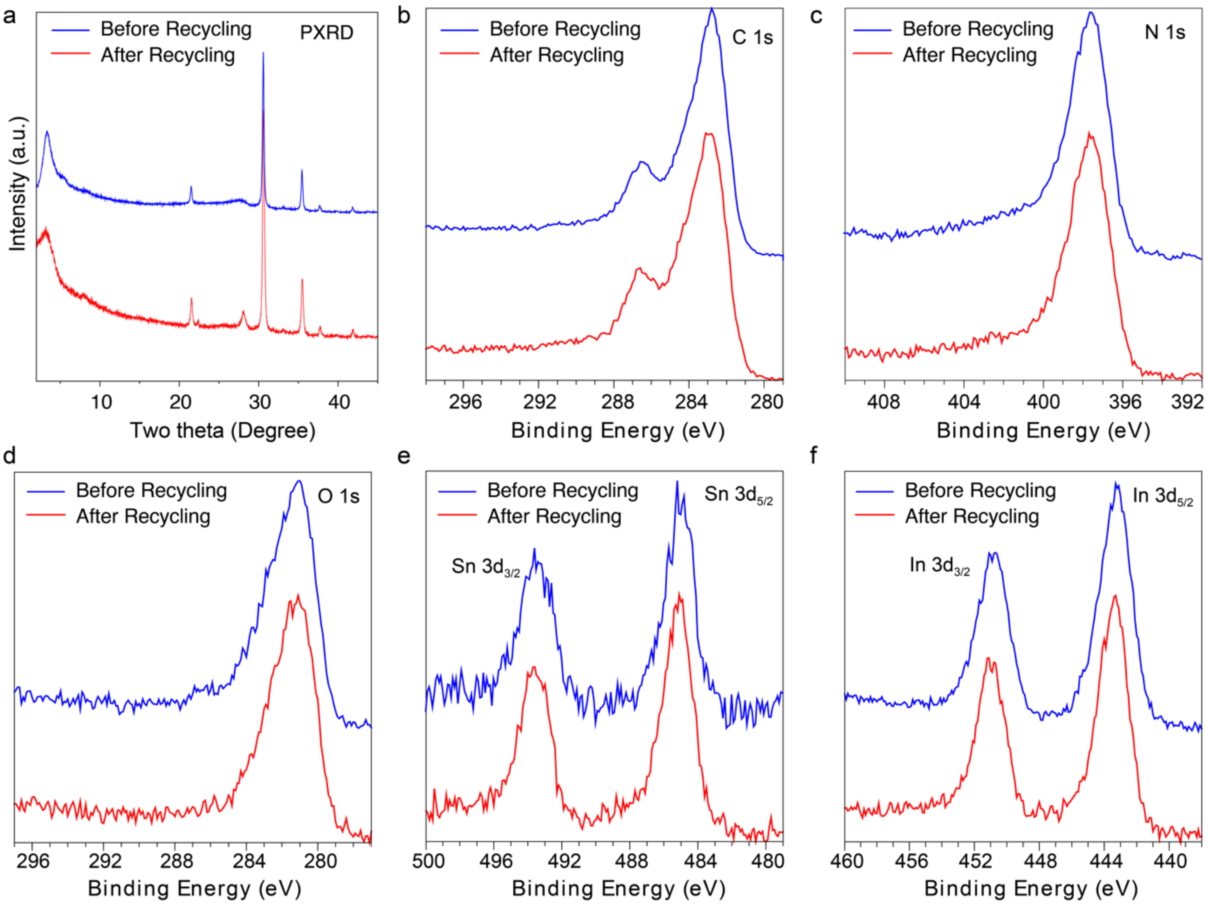

Figure S25. Characterization of the COF|ITO|CN_x composite after 6 cycle of solar reforming. a) Comparison of PXRD after the recyclability test with standard COF|ITO|CN_x composite assures all the characterizing peaks remain intact. The peak broadening of COF could be due to the decrease in COF's crystallite size during recycling several times. b–f) Comparison of XPS before and after the catalysis. The spectra highlight that the chemical bonding does not alter after the catalysis. Interestingly, the ITO nanoparticles remain impregnated within the matrix as there is no shift in Sn $3d_{3/2}$, Sn $3d_{5/2}$, In $3d_{3/2}$ and In $3d_{5/2}$ peak positions.

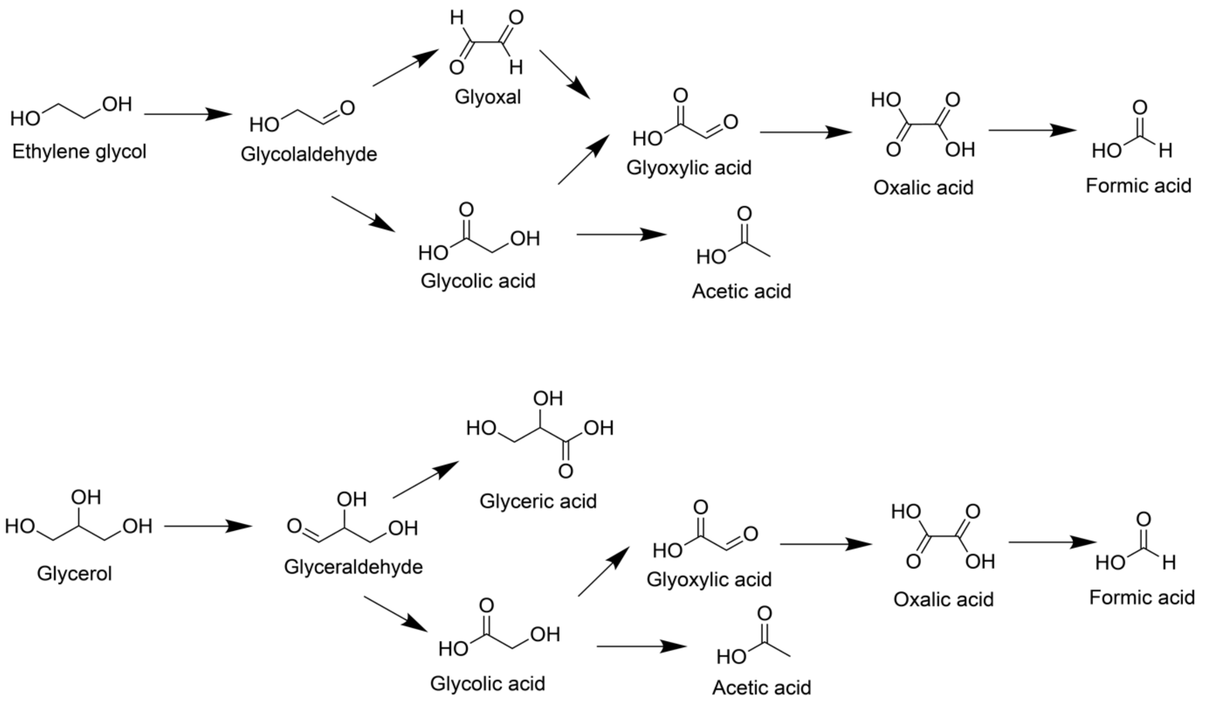

Figure S26. Product formation during ethylene glycol and glycerol oxidation. Formic acid/formate is one of the end-products during the oxidation. Further oxidation leads to the generation of CO_2.

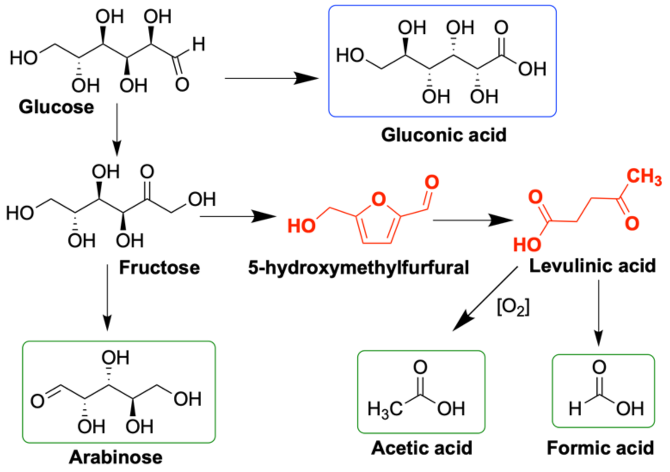

Figure S27. Product formation during sugar (glucose) oxidation. 5-Hydroxymethylfurfural and levulinic acids are key intermediates during the oxidation of glucose to formic acid and acetic acid. The details of each step during this conversion have not been shown here. Possibility of other products like glycolic acid, pyruvic acid, acrylic acid has also been ignored.

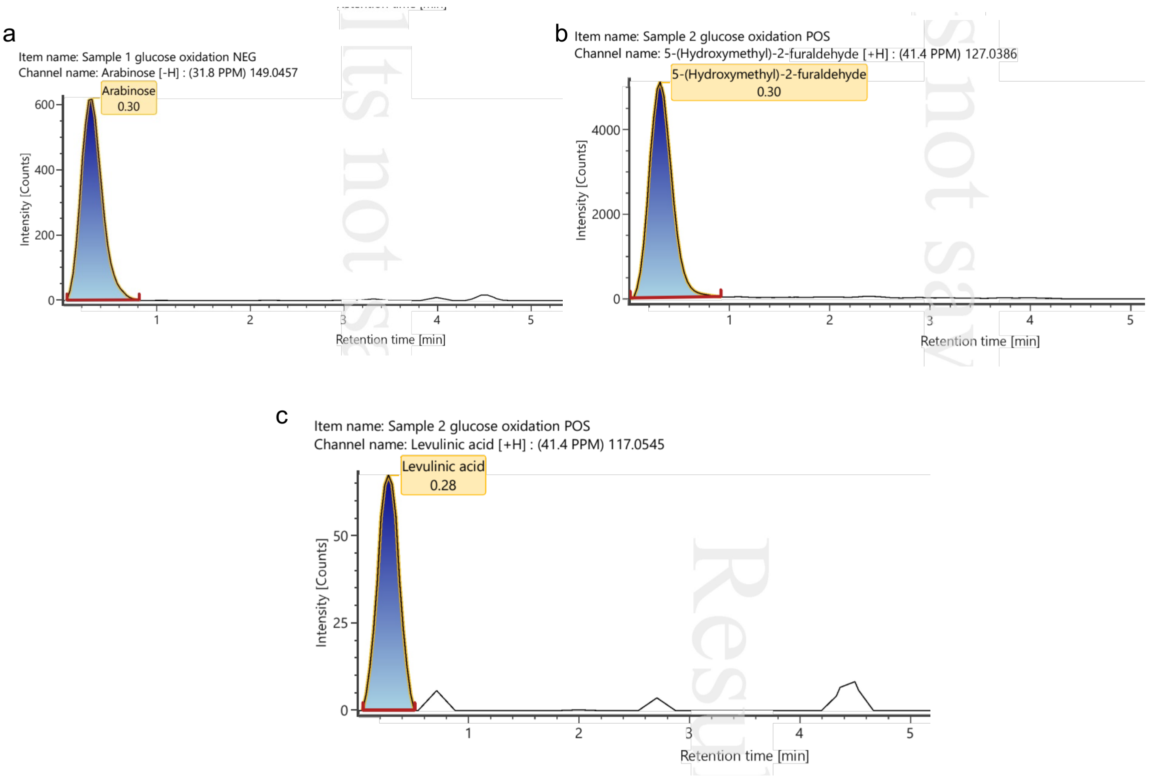

Figure S28. Liquid chromatography–mass spectrometry (LC–MS) analysis during sugar (glucose) oxidation. The analysis of the reaction mixture after 4 h of reaction confirms the formation of key intermediates that leads to the generation of formate as the final product.

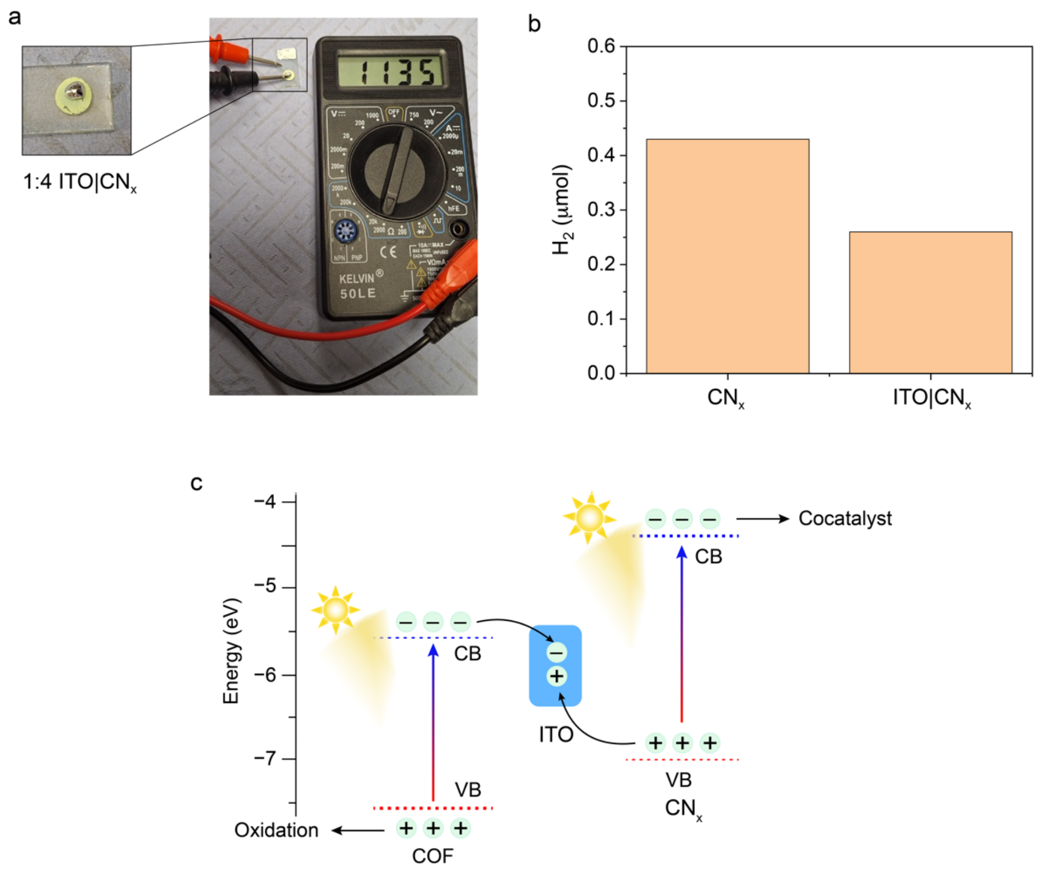

Figure S29. Mechanism of solar reforming process. a) Liquid-metal contact test using a eutectic Ga–In (75.5:24.5) droplet on CN_x film casted on glass supported ITO. The experiment confirms that ITO enhances the intrinsic conductivity. b) Controlled half reaction (H_2 evolution) to understand the role of ITO. Condition: CN_x=2 mg mL^{-1}, ITO|CN_x=2.5 mg mL^{-1} (ITO:CN_x=1:4), triethanol amine=0.05 mM, 600 rpm stirring, and irradiation (AM 1.5 G, 100 mW cm^{-2}, 25 °C, 9.5 h). c) Plausible electron transfer pathway during solar reforming with COF|ITO|CN_x composite, where ITO is a degenerate semiconductor, possessing metal-like behavior.

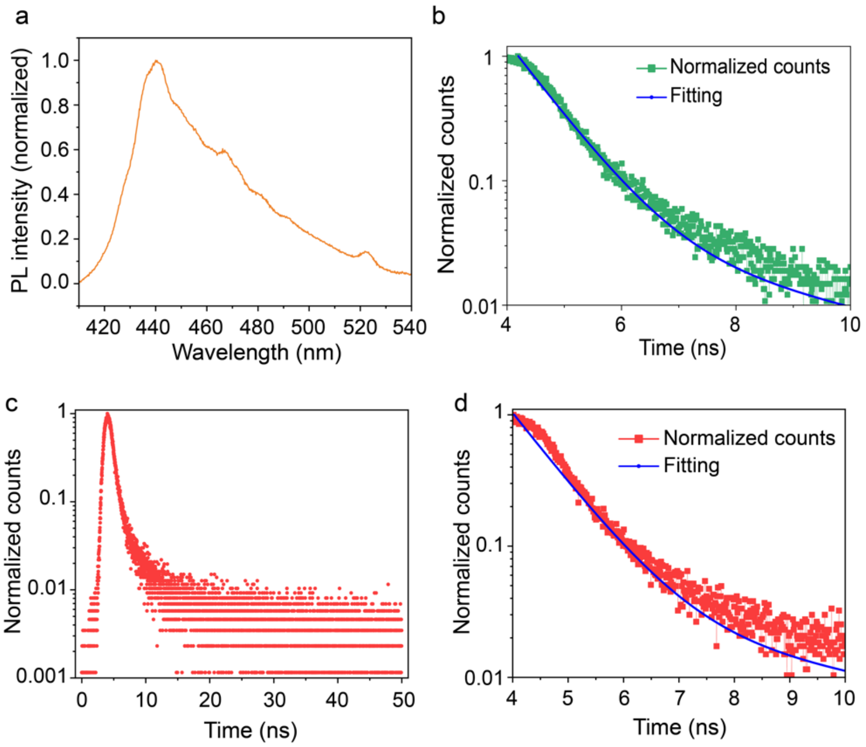

Figure S30. Photoluminescence and excited-State lifetime analysis. a) Photoluminescence spectrum of COF|ITO|CN_x. b) Fitting curve of the time-correlated single photon counting of COF|ITO|CN_x|NiME composite. The life time was measured to be 4.3 ns. c) Excited state lifetime measurement through time-correlated single photon counting of COF|ITO|CN_x composite. d) Fitting curve of the time-correlated single photon counting of COF|ITO|CN_x composite. The life time was measured to be 5.51 ns for COF|ITO|CN_x.

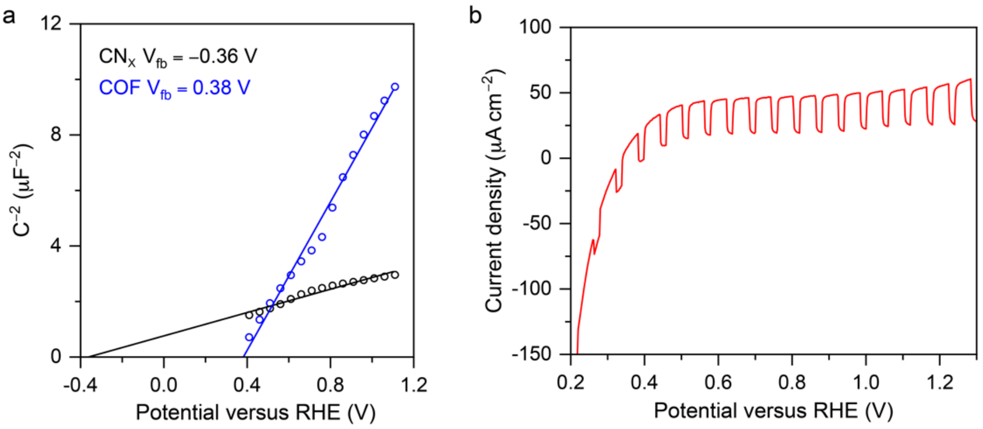

Figure S31. Electro and photoelectrochemical characterization. a) Mott–Schottky plots measured from 100 kHz to 1 Hz at pH 7 (0.5 M K_2SO_4). The flat-band potential obtained from the M–S analysis differs slightly from the Fermi level position determined by UPS analysis, likely due to the fact that M–S theory assumes ideal flat electrodes, whereas nanostructured COF and CN_x electrodes may possess partially depleted regions, resulting in behaviors that deviate from the ideal Schottky junction.[27] b) Linear sweep voltammetry (LSV) of the COF|ITO|CN_x photoelectrode. Composite showcases cathodic and anodic photocurrent under catalytic condition; electrolyte: 0.5 M K_2SO_4 containing 0.1 M EG, Ag/AgCl (sat. NaCl) reference electrode, Pt mesh counter electrode.

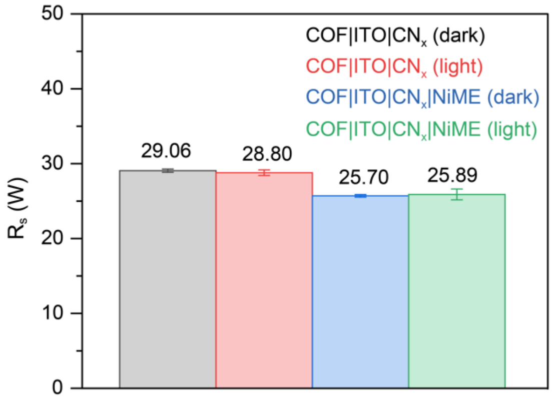

Figure S32. Measurement of photoresponses. Quantitative analysis on impedance response: R_s refer to series resistance. The plots prove that NiME acts as a cocatalyst as it can effectively mediate electrons from CN_x to reduce proton as evident from the decrease of R_s values. Error bars originated from fitting errors.

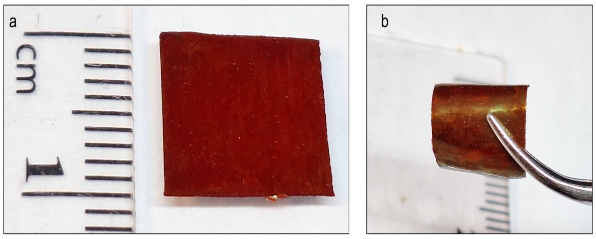

Figure S33. Graphics of a standalone photoleaf. a) Digital image of 1*1 cm^2 COF|ITO|CN_x photoleaf. b) Image of a self-standing photoleaf. These graphics prove that the COF|ITO|CN_x could be turned into any desirable size and shape based on the operational solar reforming condition.

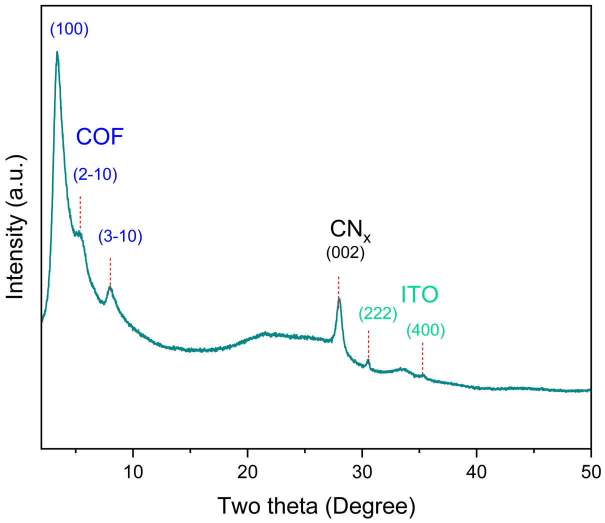

Figure S34. Powder X-ray diffraction pattern of a COF|ITO|CN_x photoleaf. The composite contains all the components and broad peak between 21 to 28 degree (two theta) confirms the intercalation and stacking of CN_x with the COF (Discussion S4, Supporting Information). As the amount of ITO was significantly lowered (half as compared to the COF|ITO|CN_x powder, Discussion S2, Supporting Information) for COF|ITO|CN_x photoleaf, the intensity of the diffraction peaks from ITO is very low and they are buried under the highly crystalline COF and CN_x peaks.

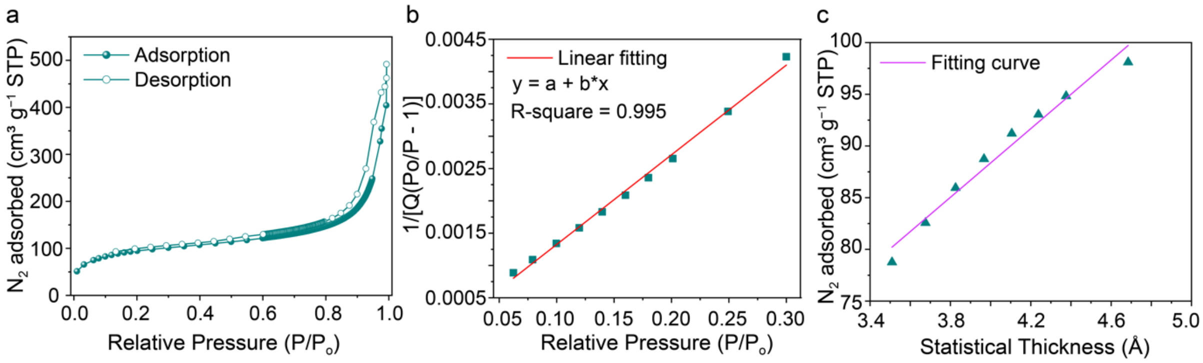

Figure S35. a) N_2 isotherm of COF|ITO|CN_x photoleaf measured at 77.5 K. b) Fitting curve for the BET surface area measurement at low relative pressure (0.05-0.3). c) t-Plot for COF|ITO|CN_x photoleaf measured at low thickness range (3-5 Å).

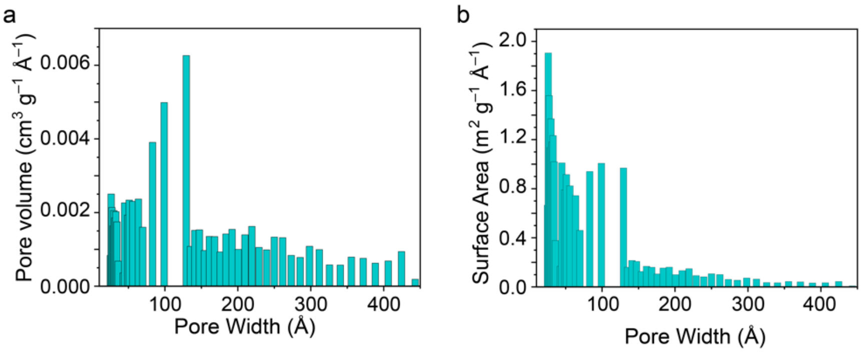

Figure S36. Pore size distribution of COF|ITO|CN_x photoleaf measured with non-linear density function theory (NLDFT). The main contribution to the high pore volume comes from the macropores. On the other hand, micropores and mesopores contributes towards the high BET surface area of the standalone photoleaf.

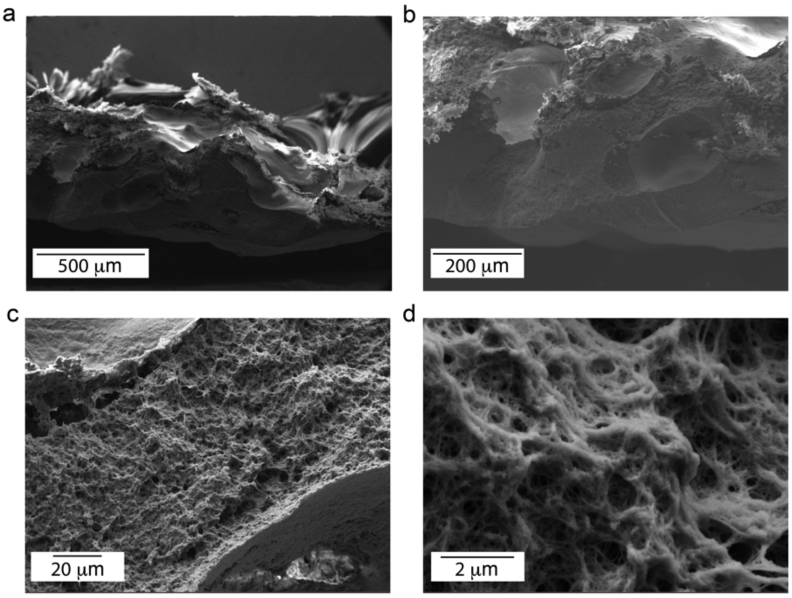

Figure S37. Scanning electron microscopy (SEM) images of the COF|ITO|CN_x photoleaf. a) Cross-section image of the photoleaf that reveals the thickness of ~ 200 µm. b–d) Uniform macroporous structure of the photoleaf.

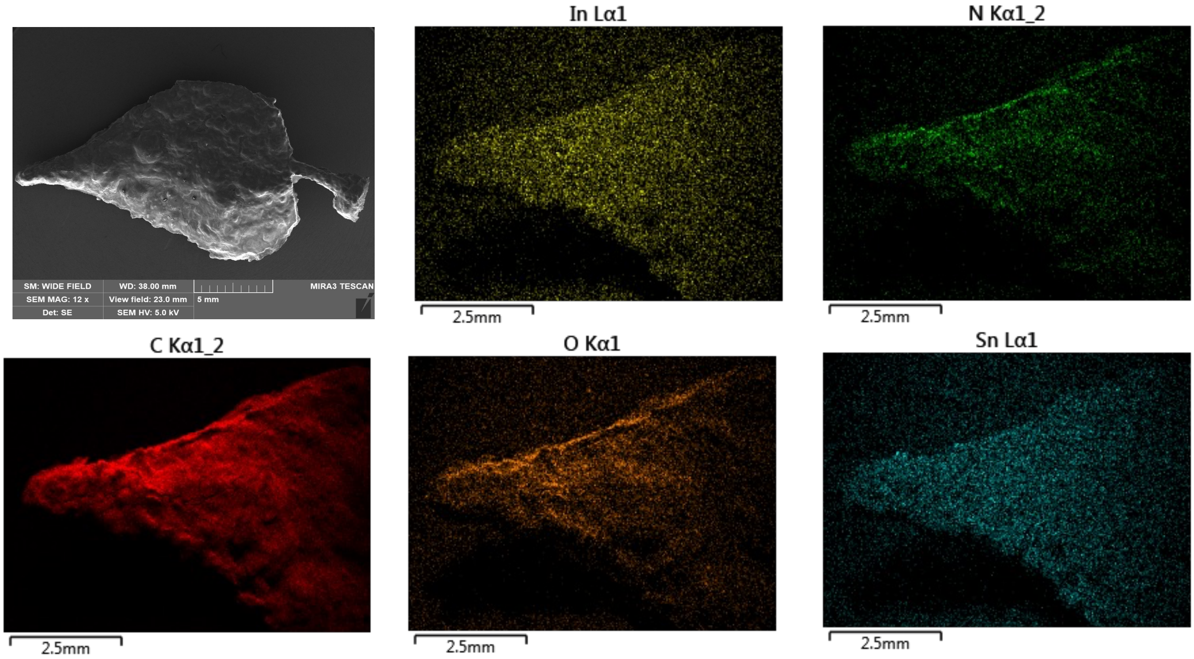

Figure S38. SEM–EDX elemental mapping image of COF|ITO|CN_x photoleaf. Elemental images highlight the composition and uniform distribution of C, N, O, In and Sn within the matrix.

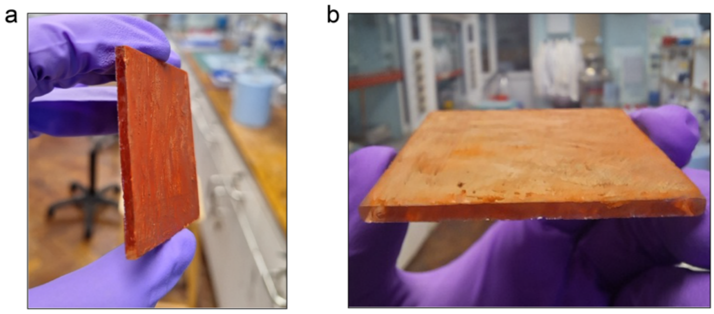

Figure S39. Digital image of COF|ITO|CN_x photopanel.

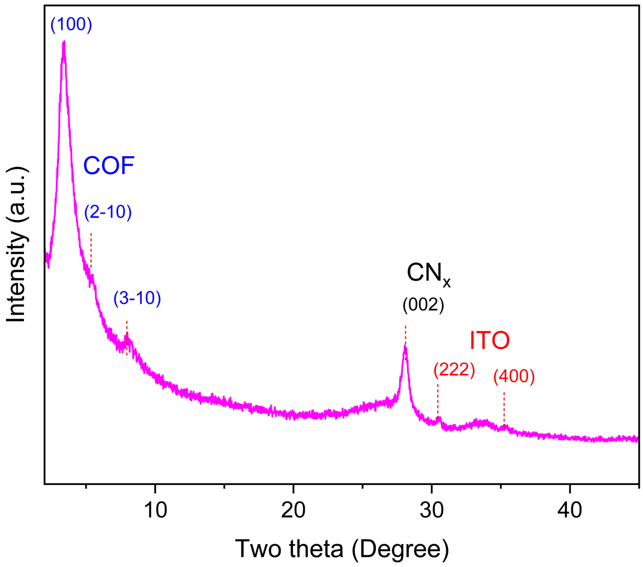

Figure S40. Powder X-ray diffraction pattern of a COF|ITO|CN_x panel. The composite contains all the components; COF, CN_x and ITO (Discussion S4, Supporting Information). As the amount of ITO was significantly lowered (1/8th of COF and CN_x amount, Discussion S2, Supporting Information) for panel, the intensity of the diffraction peaks from ITO is very low and they are buried under the highly crystalline COF and CN_x peaks.

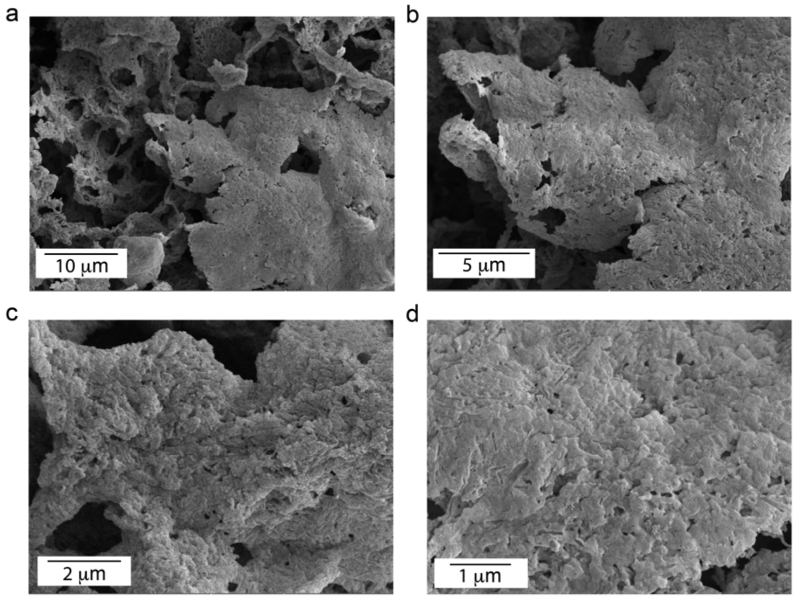

Figure S41. Scanning electron microscopy (SEM) images of the COF|ITO|CN_x panel. Here the composite has been extracted from the panel and images have been collected without any sonication or solution treatment. SEM images demonstrate the layered two-dimensional morphology of the composite. Considering the freeze-drying treatment on the panel, the macropores are generated. However, the zoomed image (d) shows the presence of uniform crystallites. Their assembly leads to construction of 2D sheets.

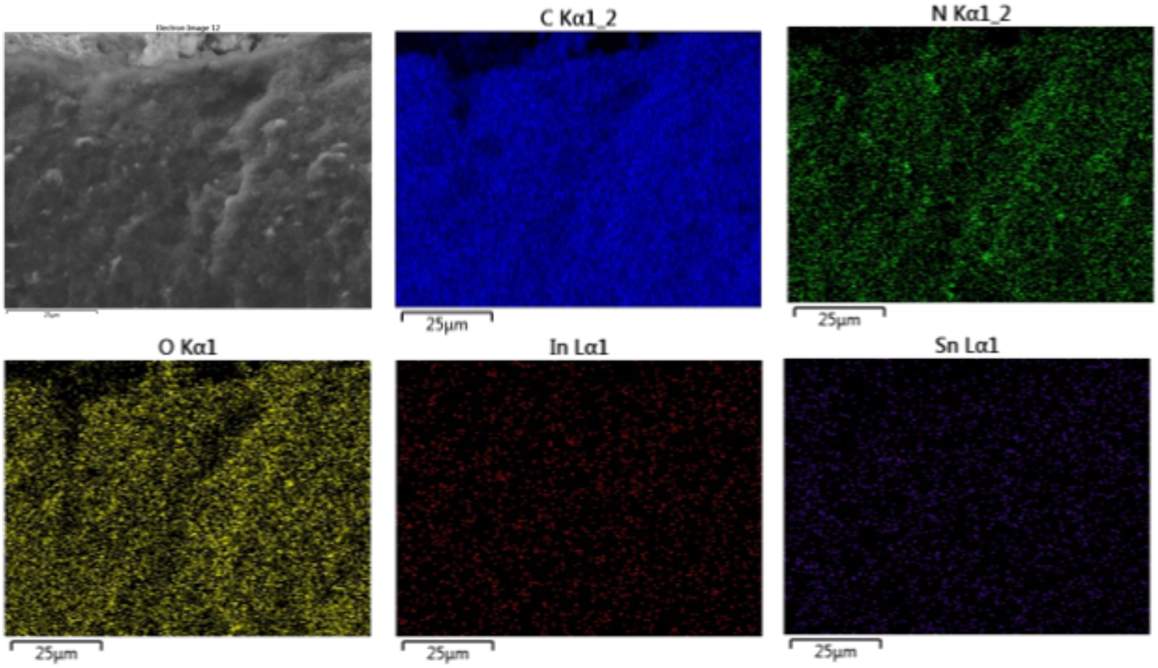

Figure S42. The top-down energy-dispersive X-ray (EDX) spectroscopy images of the COF|ITO|CN_x photopanel. The analysis confirms the presence of all the elements (carbon, nitrogen, oxygen, indium and tin) similar to the powder form of COF|ITO|CN_x composite.

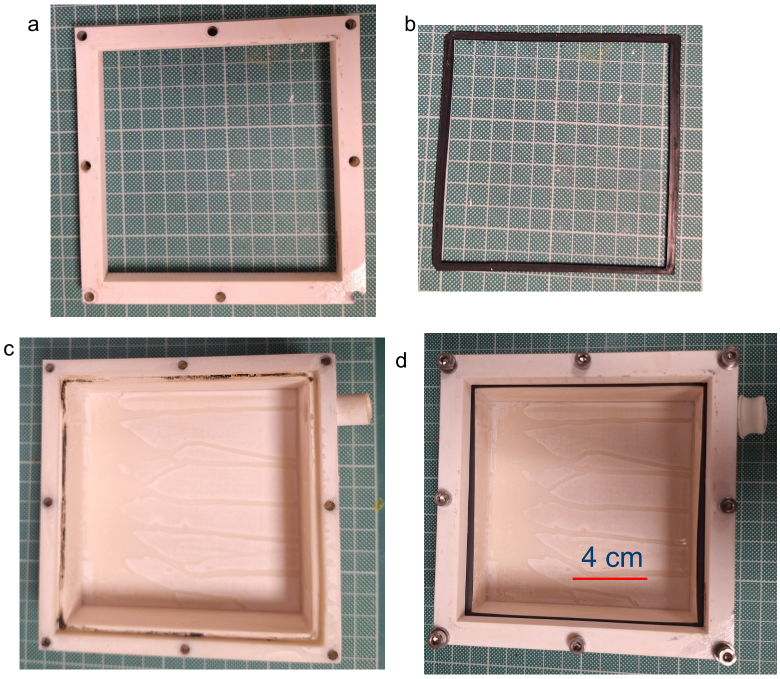

Figure S43. Digital images of a 3D-printed single-window photoreactor. a-c) Graphics of the individual parts of the reactor including frontal part (a), a gasket (b) and reservoir (c). d) A fully assembled photoreactor for front illumination.

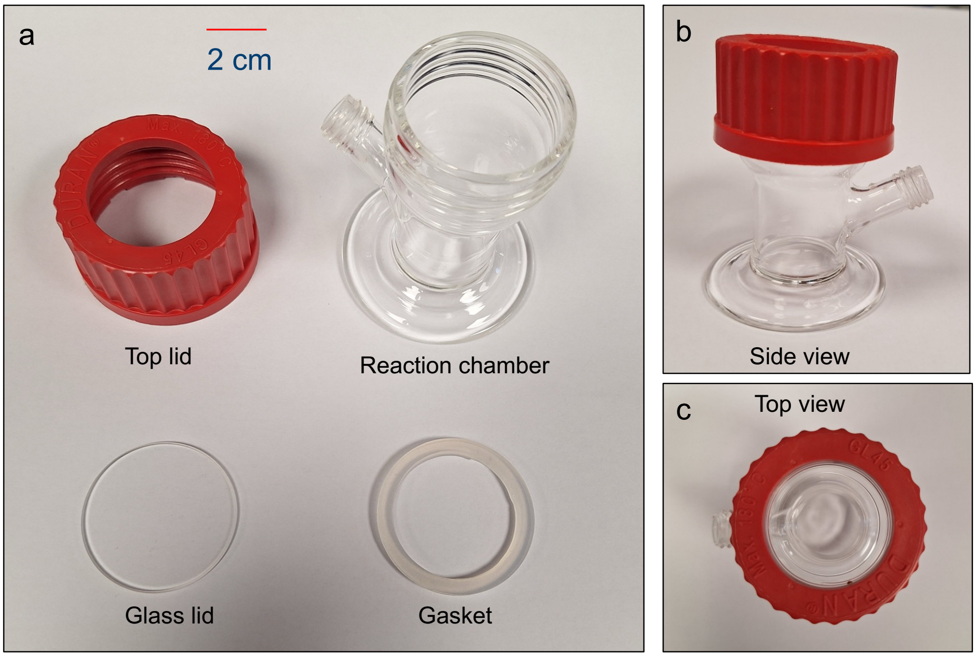

Figure S44. Assembly of a top-down photoreactor. a) Digital images of the individual parts of a top-down photoreactor. b) Side view of a fully assembled photoreactor. c) Top view of a fully assembled photoreactor.

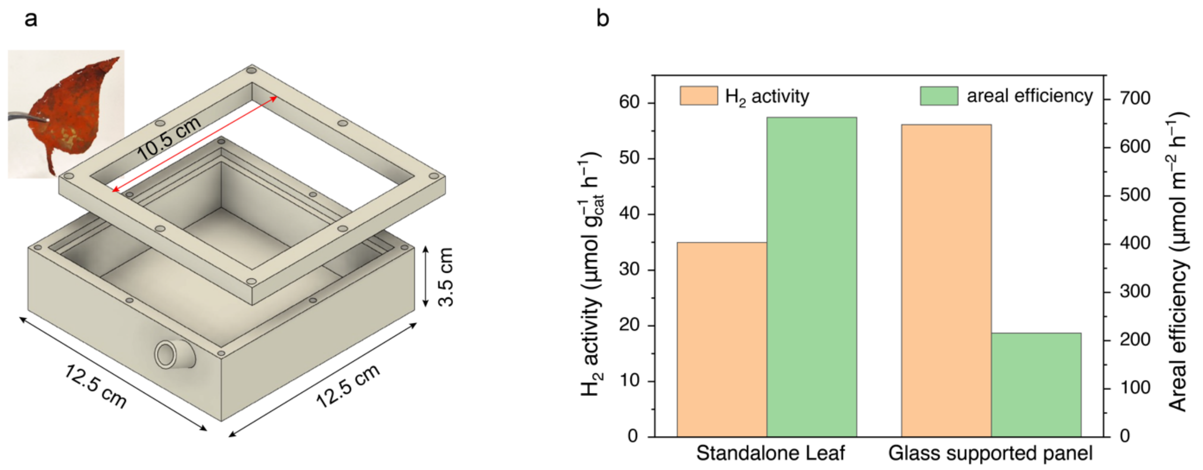

Figure S45. Comparison of solar reforming. a) Graphics of a single window reactor; inset: digital image of a stand-alone COF|ITO|CN_x|NiME photoleaf. b) Comparison of activities between a stand-alone photoleaf and glass supported photopanel; conditions: photocatalyst = COF|ITO|CN_x|NiME, H_2O as solvent, 0.5 M EG, irradiation (21 h, AM 1.5 G, 100 mW cm^{-2}, 25 °C).

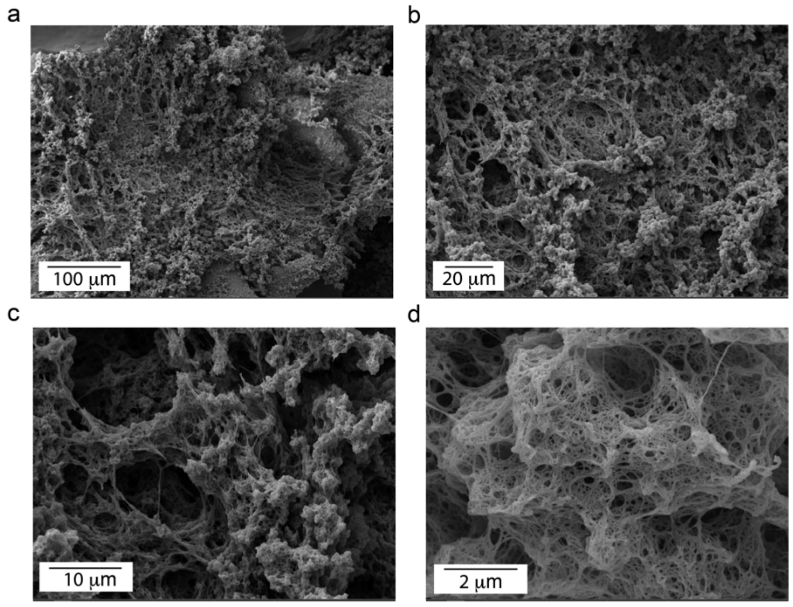

Figure S46. Scanning electron microscopy (SEM) images of the COF|ITO|CN_x|NiME photoleaf after solar reforming. The macroporous surface morphology of the photoleaf remains unaltered after solar reforming (see Figure S33b–d for the macroporous surfaces of the photoleaf before solar reforming).

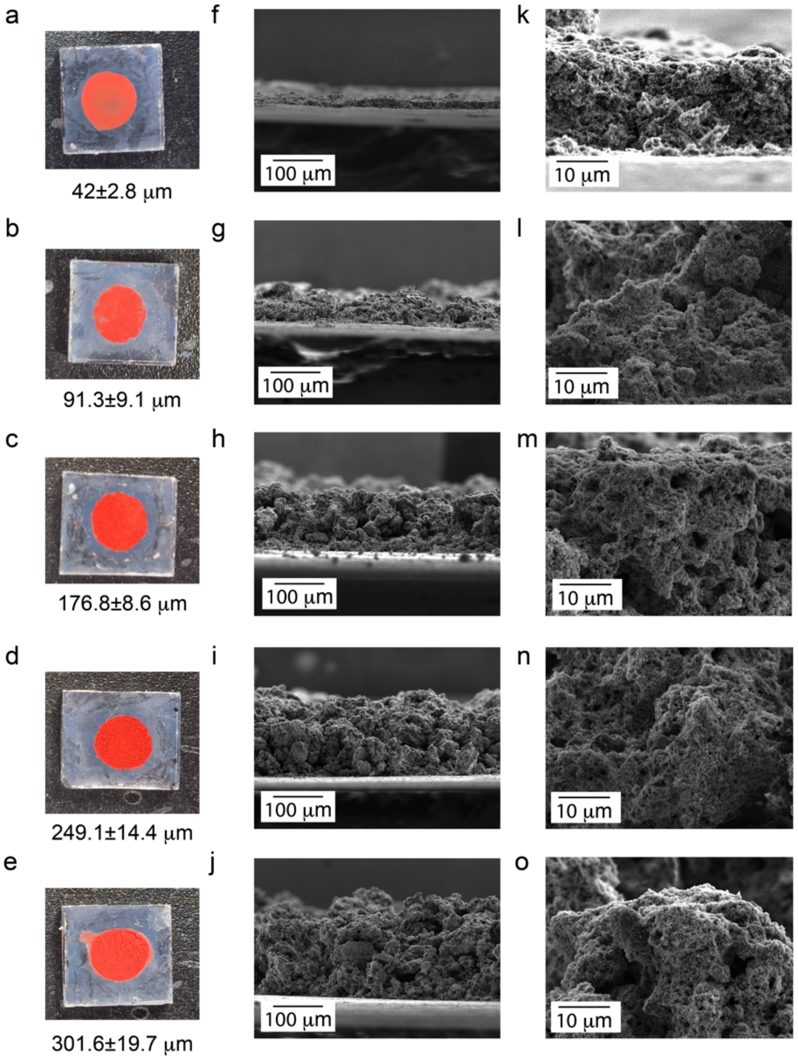

Figure S47. Thickness dependent solar reforming activity of COF|ITO|CN_x composite. a–e) Digital images of the COF|ITO|CN_x|NiME films with different thicknesses. Thickness was measured at five regions of each film, and the average value is shown at the bottom of each image. f–o) Side-view SEM images of the films, arranged next to the corresponding digital images. For example, f) and k) correspond to film a) with a thickness of 42±2.8 μm. COF|ITO|CN_x films were prepared by a controlled drop-casting method. A uniform ink was prepared by sonicating 14 mg of composite in 300 μL ethanol. Different film thicknesses were achieved by depositing varying numbers of 10 μL drops (0.16 mg per drop) onto a 0.2 cm^2 glass substrate. Between each drop, the sample was dried at 120 °C, and finally, the films were dried overnight at room temperature.

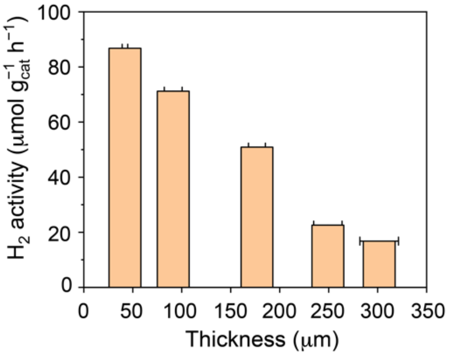

Figure S48. Thickness dependent H_2 evolution of COF|ITO|CN_x|NiME film. Condition: 500 mM EG, and top irradiation (AM 1.5 G, 100 mW cm^{-2}).

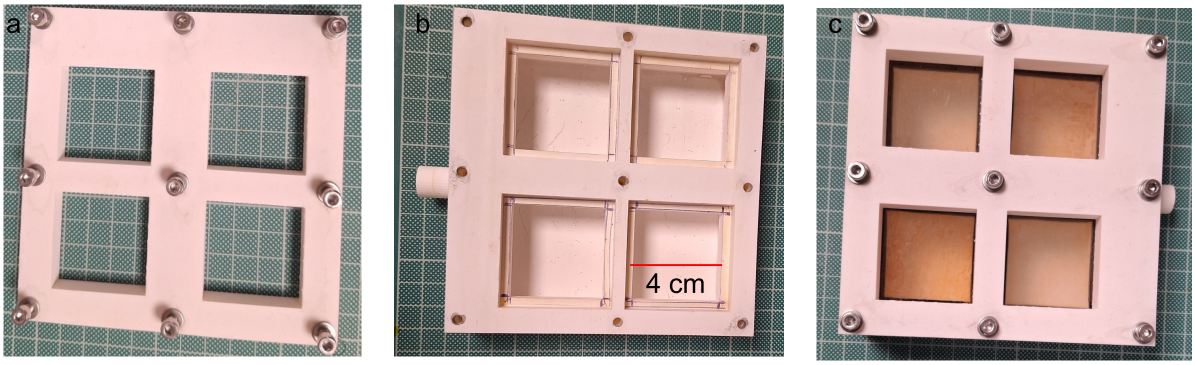

Figure S49. Assembly of a 3D-printed multi-window photoreactor for solar reforming. a) Digital image of the frontal part of the reactor. b) Digital image of the reservoir where catalysis occurs. c) Fully assembled reactor with mounted frosted glass.

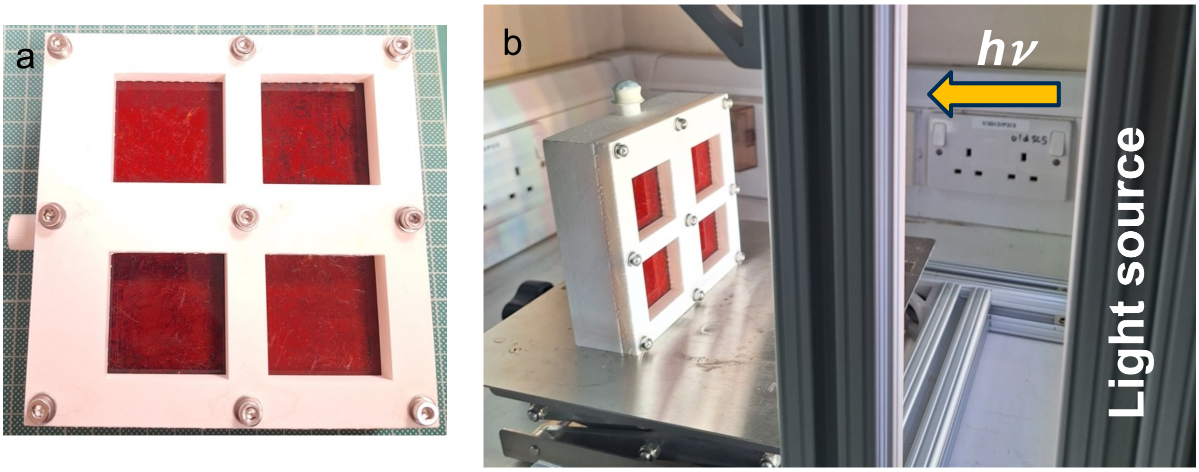

Figure S50. Operation of a multi-window photoreactor. a) Digital image of a fully assembled multi-panel reactor before operation. b) A fully assembled reactor under operation.

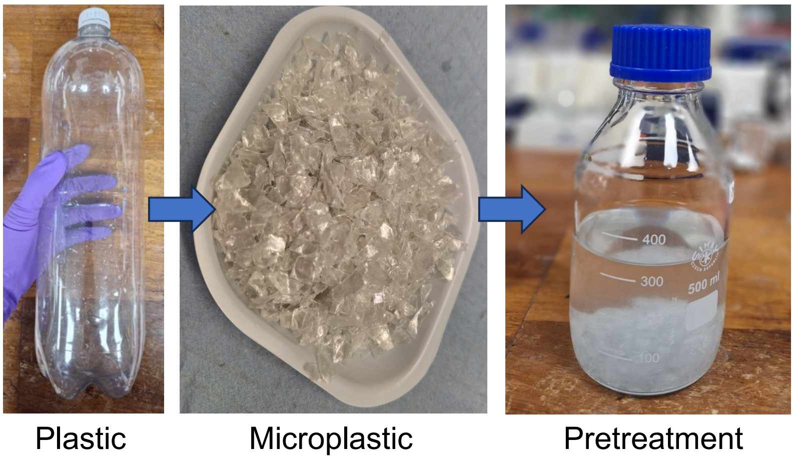

Figure S51. Pretreatment of PET plastic. A plastic bottle was first cut into small pieces followed by grinding to make the microplastics. Then, the grounded plastics was treated with 1 (M) KOH at 80 ºC for 7 days for degradation into ethylene glycol and terephthalate as main products (see Figure S52 below for NMR).

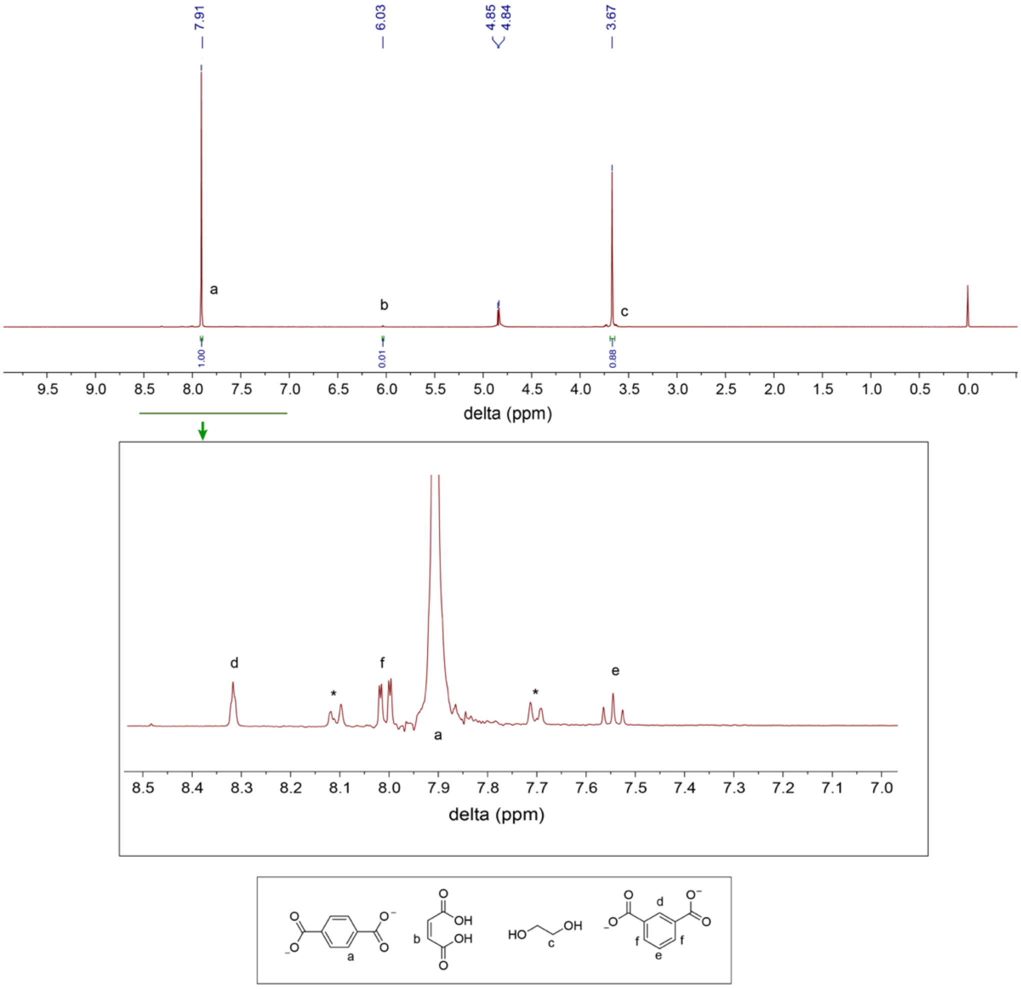

Figure S52. ^{1}H NMR spectrum (D_2O, 400 MHz) after plastic pretreatment. Microplastics were treated with 1 (M) KOH at 80 °C for 7 days with maleic acid as standard.

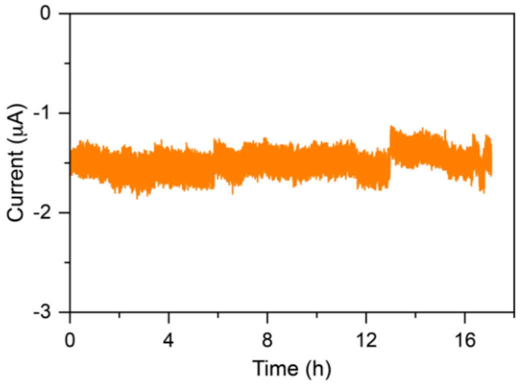

Figure S53. Chronoamperometry of a Pt electrode under alkaline conditions. Electrolyte: 1 M KOH containing EG (0.1 M). Measurement performed in a two-electrode configuration at –0.7 V applied bias, with two Pt electrodes serving as working and counter electrodes.

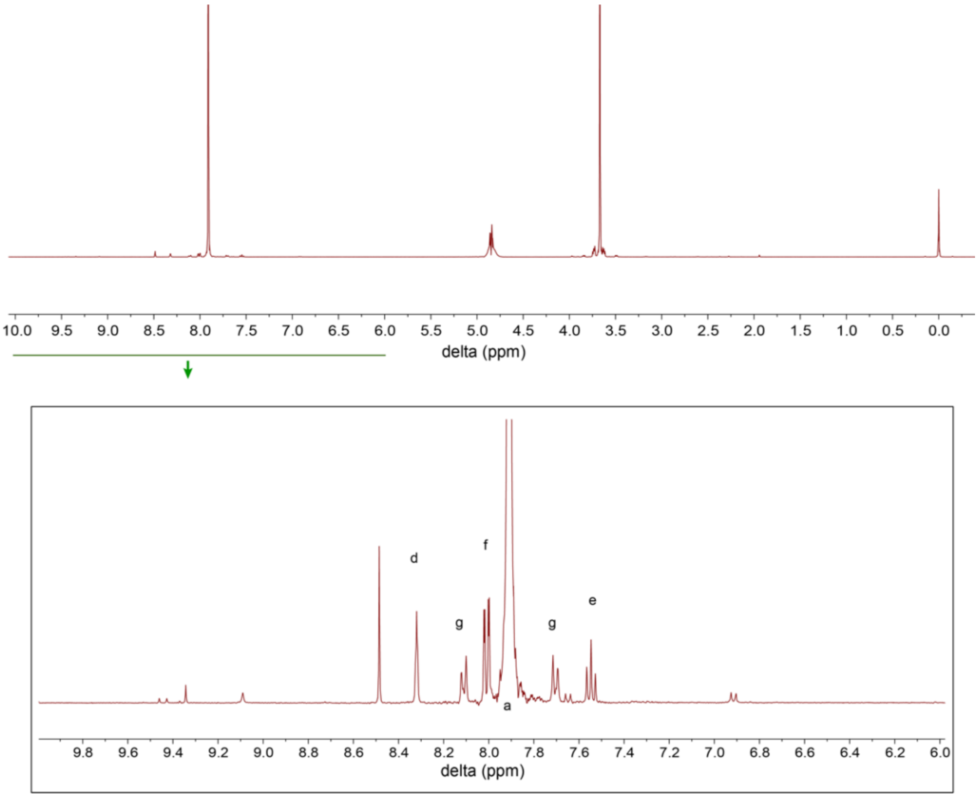

Figure S54. ^{1}H NMR spectrum (D_2O, 400 MHz) of the pretreatment solution of COF|ITO|CN_x|NiME photopanel under plastic solar reforming condition. The panel was treated with 1 (M) KOH having microplastics for 1 day under simulated sunlight (AM 1.5 G, 100 mW cm^{-2}, 55 °C).

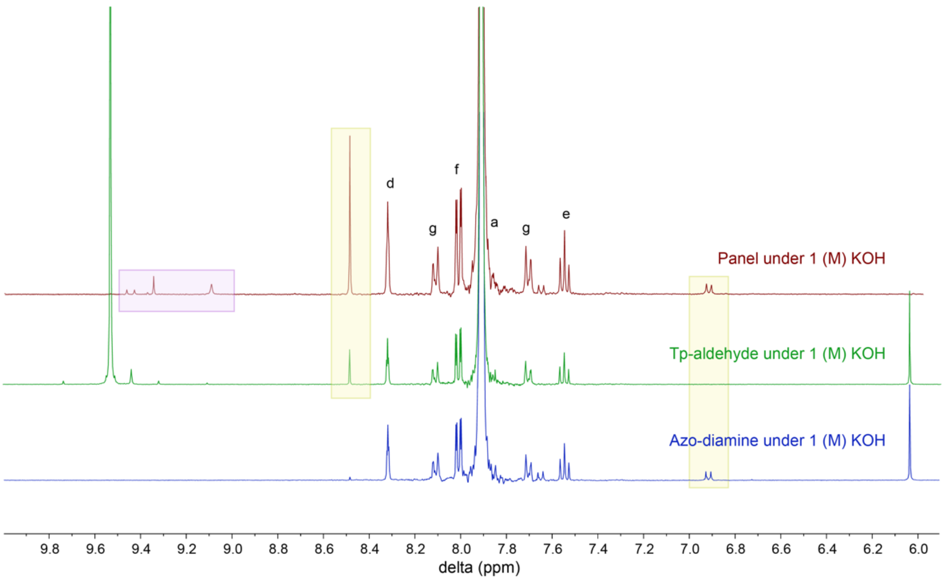

Figure S55. Comparison of ^{1}H NMR (in D_2O) of COF|ITO|CN_x|NiME panel with building units (aldehyde and amine). The NMR of aldehyde and diamine was measured in KOH solution (with D_2O) having microplastics and maleic acid as standard (peak at 6.04 ppm).

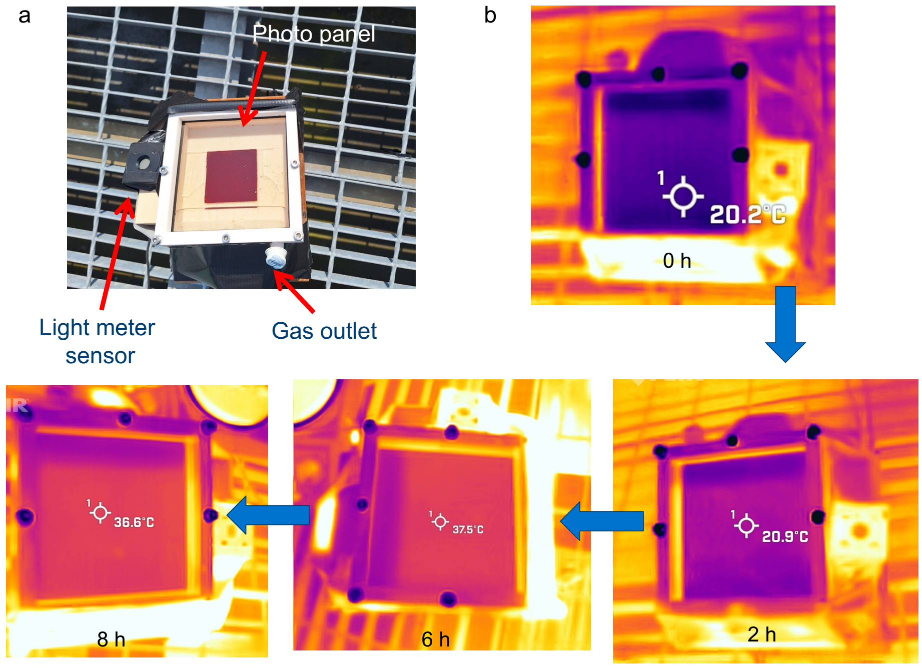

Figure S56. Reactor used and temperature measurement during solar reforming. a) Components of a 3D printed single window reactor. b) Temperature change during director sunlight driven solar reforming. Although photopanel has significant thermal and chemical stability, the temperature above 30 °C can result quicker degradation of the photopanel in 1 M KOH.

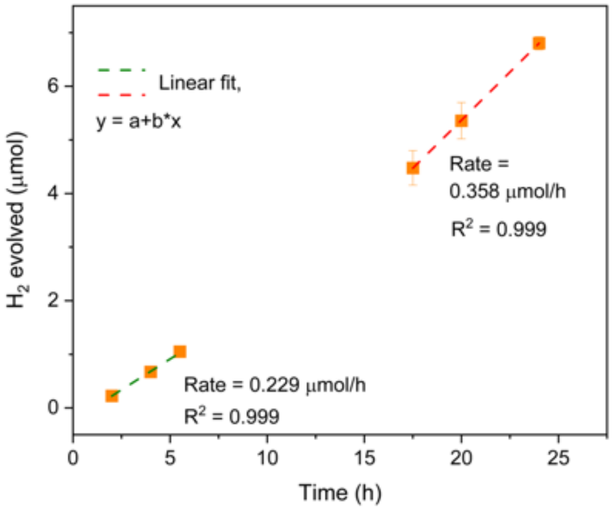

Figure S57. Rate of H_2 evolution during solar reforming at different time interval. The increase in rate of HER after 15 h is due to the diffusion of reactants through the porous matrix (reaction conditions: COF|ITO|CN_x|NiME (2 mg mL^{-1}), 0.5 M EG, 600 rpm stirring, and irradiation (AM 1.5 G, 100 mW cm^{-2}, 25 °C).

References

[1] S. Kandambeth, A. Mallick, B. Lukose, M. V. Mane, T. Heine, R. Banerjee, *J. Am. Chem. Soc.* **2012**, *134*, 19524.

[2] a) S. Mohata, P. Majumder, R. Banerjee, *Chem. Soc. Rev.* **2025**, *54*, 6062; b) H. Ran, Q. Xu, Y. Yang, H. Li, J. Fan, G. Liu, L. Zhang, J. Zou, H. Jin, S. Wang, *ACS Catal.* **2024**, *14*, 11675.

[3] A. Basak, S. Karak, R. Banerjee, *J. Am. Chem. Soc.* **2023**, *145*, 7592.

[4] Z.-B. Zhou, P.-J. Tian, J. Yao, Y. Lu, Q.-Y. Qi, X. Zhao, *Nat. Commun.* **2022**, *13*, 2180.

[5] A. Kumar Mahato, S. Bag, H. S. Sasmal, K. Dey, I. Giri, M. Linares-Moreau, C. Carbonell, P. Falcaro, E. B. Gowd, R. K. Vijayaraghavan, R. Banerjee, *J. Am. Chem. Soc.* **2021**, *143*, 20916.

[6] S. Karak, K. Dey, A. Torris, A. Halder, S. Bera, F. Kanheerampockil, R. Banerjee, *J. Am. Chem. Soc.* **2019**, *141*, 7572.

[7] S. Karak, S. Kandambeth, B. P. Biswal, H. S. Sasmal, S. Kumar, P. Pachfule, R. Banerjee, *J. Am. Chem. Soc.* **2017**, *139*, 1856.

[8] S. Karak, K. Dey, R. Banerjee, *Adv. Mater.* **2022**, *34*, 2202751.

[9] A. Khayum M, V. Vijayakumar, S. Karak, S. Kandambeth, M. Bhadra, K. Suresh, N. Acharambath, S. Kurungot, R. Banerjee, *ACS Appl. Mater. Interfaces* **2018**, *10*, 28139.

[10] A. K. Mohammed, S. Usgaonkar, F. Kanheerampockil, S. Karak, A. Halder, M. Tharkar, M. Addicoat, T. G. Ajithkumar, R. Banerjee, *J. Am. Chem. Soc.* **2020**, *142*, 8252.

[11] a) A. Oku, L.-C. Hu, E. Yamada, . *Appl. Polym. Sci.* **1997**, *63*, 595; b) L.-C. Hu, A. Oku, E. Yamada, K. Tomari, *Polym. J.* **1997**, *29*, 708.

[12] H. S. Sasmal, S. Bag, B. Chandra, P. Majumder, H. Kuiry, S. Karak, S. Sen Gupta, R. Banerjee, *J. Am. Chem. Soc.* **2021**, *143*, 8426.

[13] P. Majumder, A. Basak, H. Kuiry, H. S. Sasmal, S. Karak, P. Saha, B. Chandra, S. Sen Gupta, R. Banerjee, *J. Am. Chem. Soc.* **2023**, *145*, 18855.

[14] C. H. Sharp, B. C. Bukowski, H. Li, E. M. Johnson, S. Ilic, A. J. Morris, D. Gersappe, R. Q. Snurr, J. R. Morris, *Chem. Soc. Rev.* **2021**, *50*, 11530.

[15] D. Dai, P. Wang, X. Bao, Y. Xu, Z. Wang, Y. Guo, Z. Wang, Z. Zheng, Y. Liu, H. Cheng, B. Huang, *Chem. Eng. J.* **2022**, *433*, 134476.

[16] S. Banerjee, S. Mandal, A. K. Barua, N. Mukherjee, *Catal. Commun.* **2016**, *87*, 86.

[17] C. Pulignani, C. A. Mesa, S. A. J. Hillman, T. Uekert, S. Giménez, J. R. Durrant, E. Reisner, *Angew. Chem. Int. Ed.* **2022**, *61*, e202211587.

[18] L. Hanssen, S. Kaplan, *Problems posed by scattering transmissive materials for accurate transmittance and reflectance measurements*, **1998,** *3425*, SPIE.

[19] a) T. Kawai, T. Sakata, *Chem. Lett.* **2006**, *10*, 81; b) T. Uekert, H. Kasap, E. Reisner, *J. Am. Chem. Soc.* **2019**, *141*, 15201.

[20] T. Uekert, M. A. Bajada, T. Schubert, C. M. Pichler, E. Reisner, *ChemSusChem* **2021**, *14*, 4190.

[21] a) N. Danilovic, R. Subbaraman, D. Strmcnik, K.-C. Chang, A. P. Paulikas, V. R. Stamenkovic, N. M. Markovic, *Angew. Chem. Int. Ed.* **2012**, *51*, 12495; b) Z. Zhou, L. Wei, Y. Wang, H. E. Karahan, Z. Chen, Y. Lei, X. Chen, S. Zhai, X. Liao, Y. Chen, *J. Mater. Chem A* **2017**, *5*, 20390.

[22] A. Halder, S. Karak, M. Addicoat, S. Bera, A. Chakraborty, S. H. Kunjattu, P. Pachfule, T. Heine, R. Banerjee, *Angew. Chem. Int. Ed.* **2018**, *57*, 5797.

[23] M. Schröder, K. Kailasam, J. Borgmeyer, M. Neumann, A. Thomas, R. Schomäcker, M. Schwarze, *Energy Technol.* **2015**, *3*, 1014.

[24] V. R. Battula, G. Mark, A. Tashakory, S. Mondal, M. Volokh, M. Shalom, *ACS Catal.* **2024**, 14, 11666.

[25] H. Xiao, F.-f. Jian, K. J. Zhang, *ull. Korean Chem. Soc.* **2009**, *30*, 846.
[26] B. Lukose, A. Kuc, T. Heine, *Chem. Eur. J.* **2011**, *17*, 2388.
[27] K. Sivula, *ACS Energy Lett*. **2021**, *6*, 2549.

End of Supporting Information
